# Analysis of *PPARγ* Signaling Activity in Psoriasis

**DOI:** 10.3390/ijms22168603

**Published:** 2021-08-10

**Authors:** Vladimir Sobolev, Anastasia Nesterova, Anna Soboleva, Alexandre Mezentsev, Evgenia Dvoriankova, Anastas Piruzyan, Elena Denisova, Olga Melnichenko, Irina Korsunskaya

**Affiliations:** 1 I. Mechnikov Research Institute for Vaccines and Sera RAMS, Russian Federation, Malyy Kazennyy Lane, 5, 105064 Moscow, Russia; mesentsev@yahoo.com; 2 Centre of Theoretical Problems of Physico-Chemical Pharmacology, Russian Academy of Sciences, Russian Federation, Srednyaya Kalitnikovskaya Street, 30, 109029 Moscow, Russia; annasobo@mail.ru (A.S.); dvoriankova@mail.ru (E.D.); pirstas2000@hotmail.com (A.P.); evdenissova@rambler.ru (E.D.); marykor@bk.ru (I.K.); 3 Life Science Research and Development Department, Elsevier Inc., Rockville, MD 20850, USA; nesterova.anastasia@gmail.com; 4 Scientific Research Institute of Human Morphology, 3 Tsurupa Street, 117418 Moscow, Russia; 5 Moscow Scientific and Practical Center of Dermatovenereology and Cosmetology, Russian Federation, 17, Leninskiy Avenue, 119071 Moscow, Russia; dr.melnichenko@gmail.com

**Keywords:** psoriasis, peroxisome proliferator-activated receptor gamma (*PPARγ*), real-time PCR, ELISA, immunohistochemistry, signaling pathway

## Abstract

In our previous work, we built the model of *PPARγ* dependent pathways involved in the development of the psoriatic lesions. Peroxisome proliferator-activated receptor gamma (*PPARγ*) is a nuclear receptor and transcription factor which regulates the expression of many proinflammatory genes. We tested the hypothesis that low levels of *PPARγ* expression promote the development of psoriatic lesions triggering the *IL17*-related signaling cascade. Skin samples of normally looking and lesional skin donated by psoriasis patients and psoriatic CD3^+^ Tcells samples (*n* = 23) and samples of healthy CD3^+^ T cells donated by volunteers (*n* = 10) were analyzed by real-time PCR, ELISA and immunohistochemistry analysis. We found that the expression of *PPARγ* is downregulated in human psoriatic skin and laser treatment restores the expression. The expression of *IL17*, *STAT3*, *FOXP3*, and *RORC* in psoriatic skin before and after laser treatment were correlated with *PPARγ* expression according to the reconstructed model of *PPARγ* pathway in psoriasis.In conclusion, we report that *PPARγ* weakens the expression of genes that contribute in the development of psoriatic lesion. Our data show that transcriptional regulation of *PPARγ* expression by *FOSL1* and by *STAT3/FOSL1* feedback loop may be central in the psoriatic skin and T-cells.

## 1. Introduction

Peroxisome proliferator-activated receptors (*PPARs*) form a group of nucleus receptors that play an important role in the mammalian physiological system and function as a transcription factor [1]. There are three known *PPAR* isoforms, *PPARα*, *PPARβ/δ*, and *PPARγ*, which have significant sequence and structure homology, but exhibit different tissue distribution, selectivity, and sensitivity to ligands, which leads to the regulation of different gene sets by different receptors [2,3].

After binding to the ligand, *PPARs* form a heterodimer with the liver X receptor (LXR), then heterodimerize with the retinoid X receptor (RXR) and bind to the peroxisome proliferator response elements (PPRE) in the promoter regions of target genes [4,5].

PPARγ is the most studied *PPAR* subtype, which is expressed predominantly in the heart, adipose tissue, colon, kidneys, spleen, intestine, skeletal muscle, liver, macrophages, and skin. In the skin, *PPARγ* controls the genetic regulation of gene network expression involved in cell proliferation, differentiation, and inflammatory response [6].

There is an increased expression of *PPARγ* in skin adipocytes, where it plays a critical role in their differentiation [7,8]. *PPARγ* also has an important functional role in the regulation of skin barrier permeability as an inhibitor of keratinocyte cell proliferation and a promoter of terminal differentiation of the epidermis. In addition, being an important regulator of lipid metabolism, it stimulates the production of cholesterol and ceramides in keratinocytes [1,9].

As far as psoriasis is an inflammatory skin disease characterized by epidermal hyperproliferation and abnormal keratinocyte differentiation, proteins involved in *PPARγ* signaling can be considered as potential targets for treatment. Specific *PPARγ* ligands (such as BRL49653/rosiglitazone or pioglitazone) have been shown to inhibit the production of many inflammatory mediators and cytokines in various cell types, including monocytes, lymphocytes, and epithelial cells [10,11]. Studies in a mouse model of hyperproliferative skin disease have shown that local administration of *PPARγ* ligands thiazolidinediones family (ciglitazone and troglitazone) reduces epidermal hyperplasia [12].

Therefore, *PPARγ* can impede the progress of psoriasis, downregulating the expression of proinflammatory genes in a ligand-dependent manner, counteracting the activity of transcription factors.

Previously, we reconstructed several pathway models of molecular mechanisms of psoriasis. Models describe the transition to TH17 cell signaling during the differentiation of psoriatic T cells. In summary, genetic mutations in interleukin receptor (IL23R) may cause shift to the TH17 cells production which results in elevated levels of *IL17* and *IL22* expression, which, in turn, activates keratinocytes to release different cytokines and chemokines for attracting neutrophils and other inflammatory cells in the psoriatic lesion [13,14]. In the last work we build the model that describes a hypothesis that low activity of *PPARγ* signaling may promote psoriasis. We applied network analysis to build the model and we used public microarrays data to find statistically significant molecular cascades, cell processes, molecular regulators and expression targets of *PPARγ* [15] (see Supplementary Materials).

In this work, to test the hypothesis of low activity of *PPARγ* signaling in psoriasis, we measured gene expression of *PPARγ* and several key members of the reconstructed model in skin samples and in CD3^+^ T cells from patients with psoriasis. Additionally, we tested the expression of *PPARγ* signaling in human psoriatic skin before and after laser treatment.

## 2. Materials and Methods

### 2.1. Patients and Samples

We analyzed biopsies and peripheral blood samples from patients who were treated in the V G Korolenko Hospital, Moscow Scientific and Practical Centre of Dermatovenerology and Cosmetology. Total were analyzed from 23 patients with plaque-type psoriasis and 10 healthy controls. The age of patients varied from 25 to 56 years (Table 1). Patients were diagnosed with *Psoriasis vulgaris*. The diagnoses were confirmed by the pathomorphological examination of skin biopsies.

Local anesthesia and dermatological punch (4 mm) were used for the collection of skin samples. Healthy skin samples were taken at a distance of 3 cm from a psoriatic lesion. The research was approved by the Local Ethical Committee at the Center for Theoretical Problems of Physical-Chemical Pharmacology, Russian Academy of Science, and complies with the principles of the Helsinki Declaration. The laser treatment was provided 2–3 times a week. Skin samples were collected before the treatment and one day after the 7th laser seance.

### 2.2. Cells Isolation

For peripheral blood mononuclear cells (PBMC) isolation from the whole blood density gradient centrifugation was performed. Ficoll isolation method promoted cell extraction. 7 mL of Ficoll solution (density 1.077 g/cm^3^, “DIA-M”) was placed into a 15 mL Eppendorf conical tube and then carefully overlaid with 7 mL of the whole blood. After that the tube was centrifuged for 25 min at 1200× *g* at 4 °C. The interphase containing the cellular layer was collected from the tube and placed into a new 15-mL tube for further washing procedure. 15 mL of DPBS buffer (10× without Ca and Mg, with 0.5%Tween 20, pH 7.4) were added to the cell pellet and then centrifuged for 15 min at 400× *g* at 20 °C. The supernatant was carefully removed and the wash was repeated once with the only difference of the DPBS buffer volume (10 mL). After the last centrifugation and 500 μL of culture media (RPMI) addition, cell count and viability assessment were performed.

Isolation of total CD3^+^ T cells were obtained from PBMCs of patients and controls using a total CD3^+^ T cell isolation kit (Miltenyl Biotec, Bergisch Gladbach, Germany).

**Figure 1:**
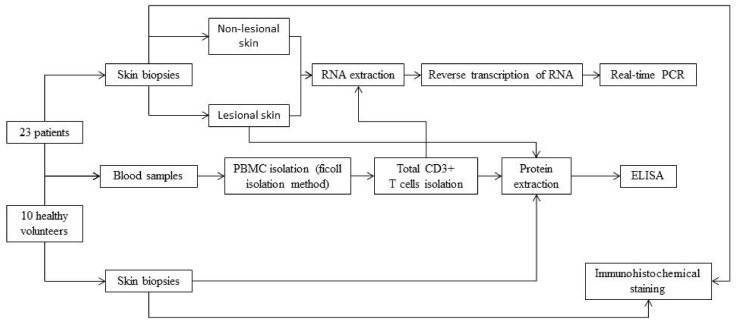
Scheme of experiment procedure.

For better presentation we summarized all methods in one scheme (Figure 1).

### 2.3. PCR

Qiagen spin column and standard RNeasy Mini Kit^®^ for the skin were used for the RNA isolation. Additional treatment of samples with the DNAase (Qiagen^®^, Germantown, MD, USA) was used to remove DNA traces. RNA concentration was measured with NanoDrop 1000 (Thermo Scientific^®^, Waltham, MA, USA).

Reverse transcription was done in 200 μL volume; the mixture included the buffer, dNTP, 100 units of reverse transcriptase (M_MLV, Promega^®^, Madison, WI, USA), 20 units of RNAses inhibitor (RNasin, Promega^®^, Madison, WI, USA), 500 ng of oligo(dT) primers (DNA-Synthes^®^, Moscow, Russia), and RNA sample (no more than 100 ng/μL). The mixture was incubated at 37 °C for 1 h.

Real-time PCR was performed in 96-well optical plates using fluorescent dyes SYBR Green (Eurogen^®^) and custom primers (DNA-Synthesis^®^). Primer sequences: *PPAR*-γ F: 5′-TCTGGCCCACCAACTTTGGG-3′ R: 5′-CTTCACAAGCATGAACTCCA-3′; *STAT3* F: 5′-ACCAGCAGTATAGCCGCTTC-3′ R: 5′-GCCACAATCCGGGCAATCT-3′; *IL17*A F: 5′- ACAACCGATCCACCTCACCTT-3′ R: 5′- CTTTGCCTCCCAGATCACAGA-3′; *RORC* F: 5′- GTAGAACAGCTGCAGTACAATC-3′ R: 5′-CTTCCAGGTCACTTGGAC-3′; *FOXP3* F: 5’-TCCCAGAGTTCCTCCACAAC-3′ R: 5′-ATTGAGTGTCCGCTGCTTCT-3′. PCR amplifier (Bio-Rad, CFX96™) was used for the amplification with the following program: (1) denaturation at 95 °C for 4 min, (2) denaturation at 94 °C for 15 s, (3–4) annealing and elongation at 60 °C for 30 s, (5) steps 2–4 were repeated 40 times. Levels of the GAPDH gene were used as a control for the expression of targeted genes. Amplification of the GAPDH gene and the studied genes was performed in different test tubes.

To calculate the results, we used numbers from real-time PCR reactions with primer efficiency at least 95%, 0.99 correlation coefficient and the curve (slope) −3.4 ± 0.2. PCR results were analyzed using the 2^−ΔΔCT^ method to compare the levels of expressions detected in affected and unaffected samples [16]. Each ΔCt was calculated as ΔCt = Ct (tested gene)−Ct (GAPDH). ΔΔCt was calculated as ΔΔCt = ΔCt (psoriatic skin sample)—ΔCt (health skin sample). The experiments were repeated three times for each sample. Intergroup differences were calculated using the Mann-Whitney U-test.

### 2.4. ELISA

Human *PPAR*-γ (Peroxisome Proliferator Activated Receptor Gamma) (MBS2503174), Signal Transducer and Activator of Transcription 3 (*STAT3*) (MBS2024094) and Interleukin 17 (*IL17*) (MBS2019491) ELISA Kits (MyBioSource, Inc., San Diego, CA, USA) was applied to detect the *PPARγ* levels in lesional and healthy skin according to the manufacturer’s protocol. Briefly, standards and tested samples prepared in assay buffer were loaded on 96-well plate and incubated with immobilized specific antibody for 1 h at 37 °C. After washing with provided solution, the specific antibody conjugated with HRP-streptavidin was added and incubation continued for another 30 min. Then, the presence of antigen was visualized with chromogenic substrate (TMB) and assayed using a microplate reader (RT-2100C, Rayto) at wavelength 450 nm. The antigen was quantified with a standard curve generated with standards of known concentration. The standard curve was constructed by plotting the mean absorbance obtained from each standard against its concentration. The calculation was done using a professional software “Curve Expert 1.4”.

### 2.5. Immunohistochemistry Analysis

Preparation of a paraffin block. To prepare skin micro-sections, a tissue samples up to 5 mm in size was fixed on a substrate to prevent wrinkling. The tissue was fixed in 10% neutral buffered formalin for 24 h at room temperature. Then formalin was washed out of the sample in running water for 6–7 h. Then the tissue was dehydrated in ethyl alcohols of ascending density: 80%-24 h, 96%-24 h, 100%-4 h. To prepare the paraffin block, the sample was kept in a 50/50 ethanol/toluene solution for 40 min at room temperature. Then the skin was kept in 100% toluene for 1 h. The tissue was also kept for 1 day in a 50/50 paraffin/toluene solution at 56 °C for successful penetration of paraffin into the sample. After that, the sample was placed in melted paraffin and kept for 2 days. A paraffin block with the skin sample enclosed in it was prepared with the use of a mold.

Staining of paraffin sections. Paraffin microsections of the human skin samples were obtained with the use of MC-2 sledge microtome. Microsections were placed on positively charged superfrost plus slides. The antigens were visualized by the NOVOLINK imaging system based on the unique compact polymer RE7290-K, designed to visualize mouse immunoglobulins M, G and rabbit immunoglobulins G of primary antibodies. For immunohistochemical staining the sections were dewaxed: toluene for 3 min, 96% ethanol for 3 min, 80% ethanol for 3 min, H_2_O for 5 min. Triton X-100 was used to perform antigen unmasking procedure.

### 2.6. Data Analysis

Literature biomedical network Resnet-2020 and software Pathway Studio were used for enrichment analysis, network analysis and pathway models reconstruction (www.pathwaystudio.com). Resnet—2020 includes interactions between proteins, drugs, diseases, mutations, cells and other biomedical entities and is based on results of text-mining of 3.5 Mln full texts papers and 24 Mln abstracts.

## 3. Results

### 3.1. *PPARγ* Expression Is Slightly Downregulated in Psoriatic Skin and CD3^+^ T Cells

For each of 23 patients, we compared the expression levels of *PPARγ* in the psoriatic skin samples and unlesional skin collected at the distance of 3 cm from the nearest psoriatic plaque. This was necessary to minimize the influence of disease-irrelevant factors on the molecular profile of selected genes [17].

**Figure 2:**
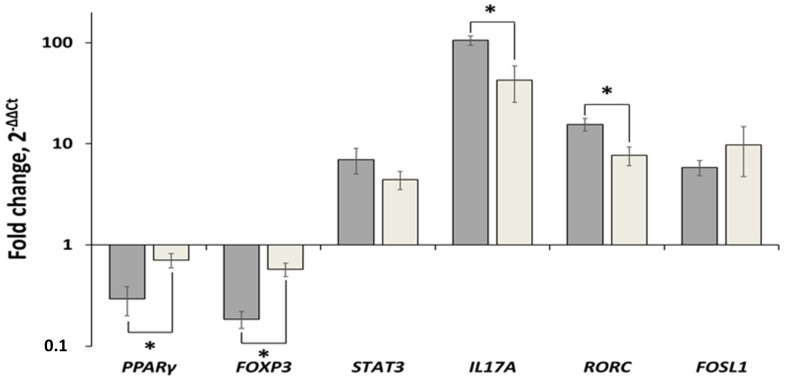
Comparative analysis of changes in the expression levels of *PPARγ*, *STAT3*, *IL17A*, *RORC*, *FOXP3* and *FOSL1* in lesional skin and CD3^+^ T cells of psoriasis patients. Dark grey bars—lesional vs. uninvolved skin; Light grey bars—CD3^+^ cells of psoriasis patients vs. same cells of healthy volunteers. The level of gene expression in control group was set to 1. Statistically significant changes in gene expression (*p* < 0.05) are marked with asterisk sign (*).

The results of real-time PCR showed that *PPARγ* was downregulated in lesional skin compared to uninvolved skin. The expression level of *PPARγ* in lesional skin was slightly reduced in 1.41 ± 0.27 times (Figure 2). We also found a significant increase in the expression levels of the following genes—*IL17* (42.39 ± 16.68), *STAT3* (4.42 ± 0.90), *RORC* (7.68 ± 1.62, and *FOSL1* (9.72 ± 4.98). In contrast, the expression level of *FOXP3* was decreased in 1.72 ± 0.14 times.

In the CD3^+^ T-cells of psoriasis patients, the expression level of *PPARγ* was reduced in 3.4 ± 0.4 times and *FOXP3*—in 5.4 ± 0.16 times compared to healthy volunteers (Figure 2). Moreover, the following genes were upregulated in CD3^+^ T cells of psoriasis patients—*IL17* (105.2 ± 11.01), *STAT3* (6.98 ± 1.96), *RORC* gene in 15.52 ± 2.18, and *FOSL1* (5.79 ± 0.99).

Since we proposed that the pathogenicity of downregulated *PPARγ*-downstream signalling is different in various types of cells, where this pathway was active, we compared the expression levels of *PPARγ* and the related genes in the CD3^+^ T cells obtained from the blood of psoriasis patients and lesional skin with similar parameters in CD3^+^ T cells of healthy volunteers and uninvolved skin, respectively. We found that the expression levels of *PPARγ* and *FOXP3* were decreased in psoriatic CD3^+^ T cells compared to lesional skin of the same individuals. The observed changes were statistically significant (*p* = 0.016 and >0.001, respectively). In contrast, the expression levels of four other genes were increased. The changes in the expression levels of *IL17A* and *RORC* were statistically significant (*p* = 0.004 and 0.033, respectively). In the same time, the changes in the expression levels of *STAT3* and *FOSL1* were not significantly different (*p* = 0.410 and 0.278, respectively).

**Figure 3:**
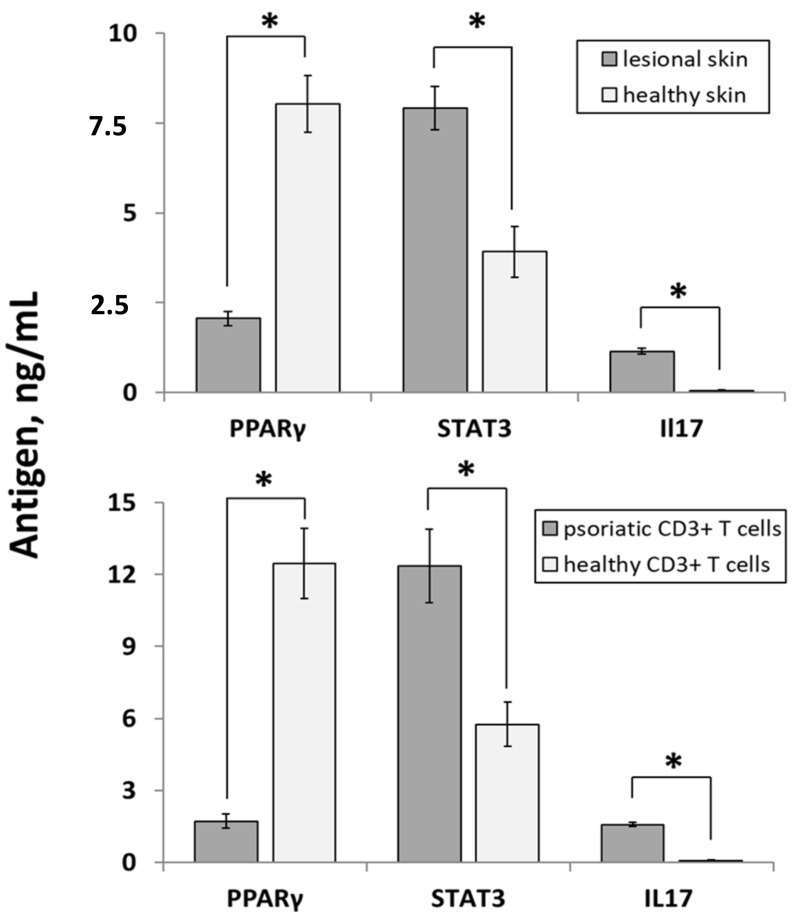
The expression levels of selected proteins in the samples obtained from psoriasis patients (*n* = 23) and healthy volunteers (*n* = 10) assessed by ELISA. The upper panel—lesional vs. healthy skin. The lower panel—samples of CD3^+^ T cells of psoriasis patients and healthy volunteers. Statistically significant changes in gene expression (*p* < 0.05) are marked with asterisk sign (*).

Using an independent method of analysis, we confirmed the differential expression of *PPARγ*, *STAT3* and *IL17* on protein level. The results of ELISA experiments performed on the same group of skin samples (Figure 3, upper panel) discovered significantly higher expression levels of *STAT3* and *IL17* in lesional skin. The expression levels of *STAT3* were 7.91 ± 0.61 and 3.92 ± 0.70 ng/mL (*p* < 0.001) whereas the expression levels of *IL17* were 1.15 ± 0.06 and 0.09 ± 0.01 ng/mL (*p* < 0.001), respectively. In contrast, the expression of *PPARγ* was reduced in lesional skin, compared to healthy skin 2.06 ± 0.20 and 8.02 ± 0.79 ng/mL, *p* < 0.001).

Expectedly, a similar expression pattern was discovered in CD3^+^ T-cells (Figure 3, lower panel). The expression levels of *STAT3* were 12.35 ± 1.53 and 5.75 ± 0.95 ng/mL (*p* < 0.001) whereas the expression levels of *IL17* were 1.57 ± 0.09 and 0.07 ± 0.02 ng/mL (*p* < 0.001) in psoriatic and healthy CD3^+^ T-cells, respectively. In the same time, the expression of *PPARγ* was reduced in psoriatic CD3^+^ T-cells, compared to same cells of healthy individuals (1.71 ± 0.29 and 12.46 ± 1.47 ng/mL, *p* < 0.001).

To reveal the differences in gene and protein expression between genders we compared the data obtained from male and female patients (Table 2), female patients with and without menopause (Table 3) as well as male and female healthy volunteers (Table 4). The following analysis did not reveal significant gender-specific changes with two exceptions. Firstly, female patients that did not experience menopause had a significantly higher expression of *FOSL1* in CD3^+^ T-cells (Table 3). Secondarily, healthy female volunteers seemed to have a higher expression of *IL17*A compared to their male counterparts (Table 4).

However, we had several reasons to doubt the significance of these findings. Primarily, the differences reported in Table 3 and Table 4 were not confirmed independently. In the first case, the significance of qPCR data was not confirmed by ELISA (Table 3). In the second case, the significance of the findings discovered by ELISA was not confirmed by qPCR (Table 4). Moreover, there we noticed a high data variability within the groups. As we believed, the patients’ comorbidities and unreported health issues of volunteers might influence the gene and protein expression. We also have to acknowledge that levels of female sex hormones significantly vary on different stages of the menstrual cycle whereas we drew the blood a day prior discharging the patients and disregarded this matter when we tested healthy volunteers. In addition, the significance of the changes in the expression of *IL17*A could be questioned because of a relatively small sample size (Table 4). Thus, we suggest that there is no association between gene and protein expression and the participants’ gender. As we believe, the obtained results do not support the hypothesis that gender could be a risk determinant of psoriasis.

Immunohistochemical skin section profile show that the accumulation of *IL17* is increased in the skin with the development of psoriatic plaque, as compared with the visually unaffected and healthy skin. Also, immunostaining of antibodies against *IL17* showed the staining of the keratinocyte cytoplasm mostly in the suprabasal epidermal layer. In the hyperplastic epidermis, the accumulation of *IL17* is more intense and heterogeneous. At the same time, *IL17* accumulates to a lesser extent in the visually unaffected skin and is only slightly detected in the healthy skin.

**Figure 4:**
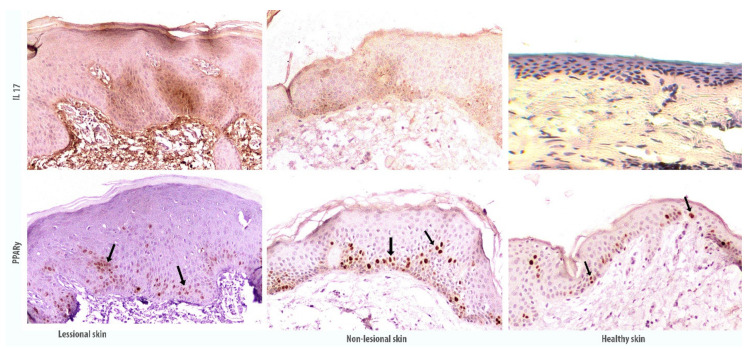
Immunohistochemical staining of antigens in the affected and non-affected psoriatic skin in comparison with the skin of healthy donors. The image was magnified 100×. Black arrows indicate *PPARy* accumulation in the suprabasal layer of epidermal keratinocytes.

In the sections presented, a more intense *PPARy* immunostaining is observed in differentiated suprabasal keratinocytes of the unaffected skin, and less in the tissues of the psoriatic plaque, despite keratinocyte proliferation and hyperplasia development (Figure 4).

### 3.2. Low Laser Treatment Stabilises *PPARγ* Related Signaling in Psoriatic Skin

**Figure 5:**
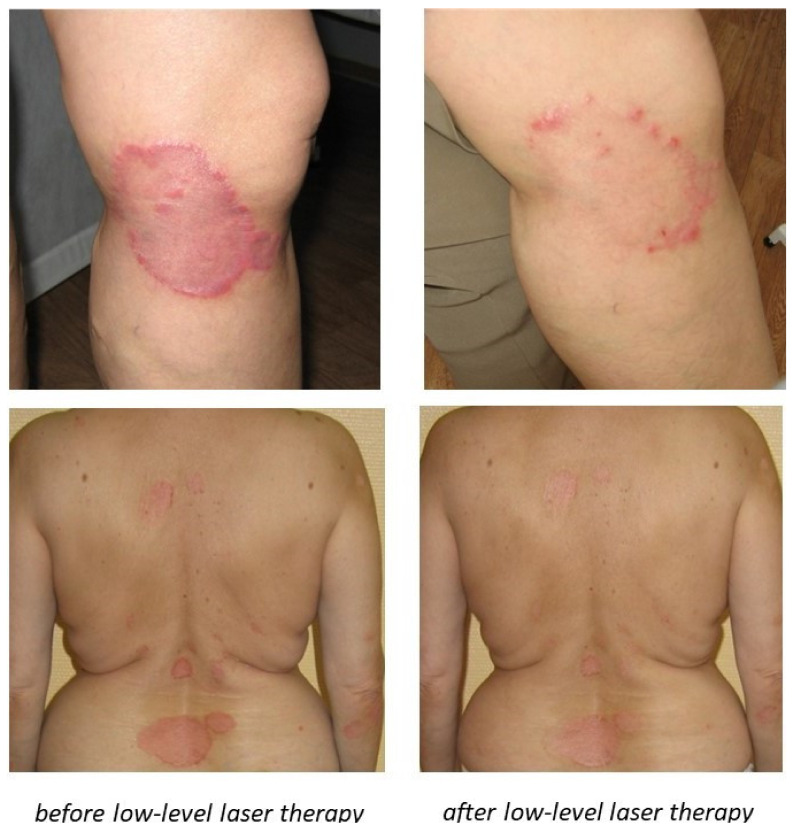
Visual positive effect after low-level laser therapy. Reduction of psoriatic skin inflammation was achieved.

For the next step of validation, we studied the expression of *PPARγ*, *STAT3*, *IL17A*, *RORC*, *FOXP3*, and *FOSL1* in human psoriatic skin samples and visually healthy skin samples before and after laser treatment. Patients received low-intensity laser treatment with 1.27 microns wavelength (infrared short waves). Similar to previously published results by different groups of medical researchers, the low-laser treatment had a positive effect on the health of observed patients and reduction of psoriatic skin inflammation was achieved (Figure 5).

**Figure 6:**
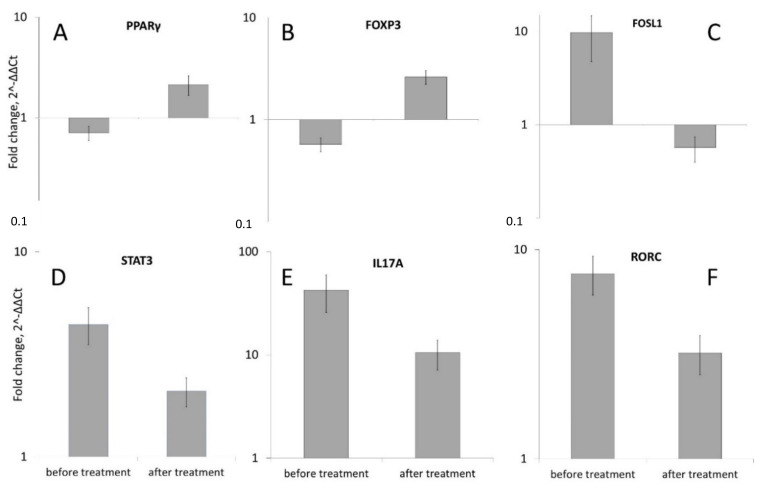
Comparison of *PPARγ* (**A**), *FOXP3* (**B**), *FOSL1* (**C**), *STAT3* (**D**), *IL17A* (**E**) and *RORC* (**F**) genes expression in the skin of 23 patients with psoriasis before and after low-level laser therapy. The levels of mRNA concentration for genes in psoriatic skin samples was calculated in relation to the level of the same genes in unaffected skin samples (which was taken as conditional 1, *p* < 0.05). See supplemental materials for detailed statistics (“PPARG expression file”).

We detected a reliable reduction in the expression of studied *PPARγ*, *STAT3*, *IL17A*, *RORC*, *FOXP3* and *FOSL1* genes after low level (1.27 microns) laser treatment. The level of *STAT3* expression was decreased in 2.08 ± 0.33 times (Figure 6D), *IL17A* in 10.48 ± 3.36 times (Figure 6E), *RORC* in 3.20 ± 0.68 times (Figure 6F) and *FOSL1* in 0.57 ± 0.17 (Figure 6C). The level of the expression of *PPARγ* was increased 2.13 ± 0.47 times (Figure 6A). The level of *FOXP3* was also increased in 2.62 ± 0.39 times (Figure 6B).

Therefore, low laser treatment caused significant growth of the *PPARγ* and *FOXP3* expression while reducing the expression of *STAT3*, *IL17A*, and *RORC*.

## 4. Discussion

Previously, we built the model of *PPARγ* dependent pathways involved in the development of the psoriatic lesions. The model includes significant molecular cascades such as *IL17* signaling, Toll like receptor and PI3K-AKT pathways from literature network analysis and public microarrays data. In this work we tested the model by measurement mRNA and protein levels of key molecular players in human psoriatic skin and T-cells.

**Figure 7:**
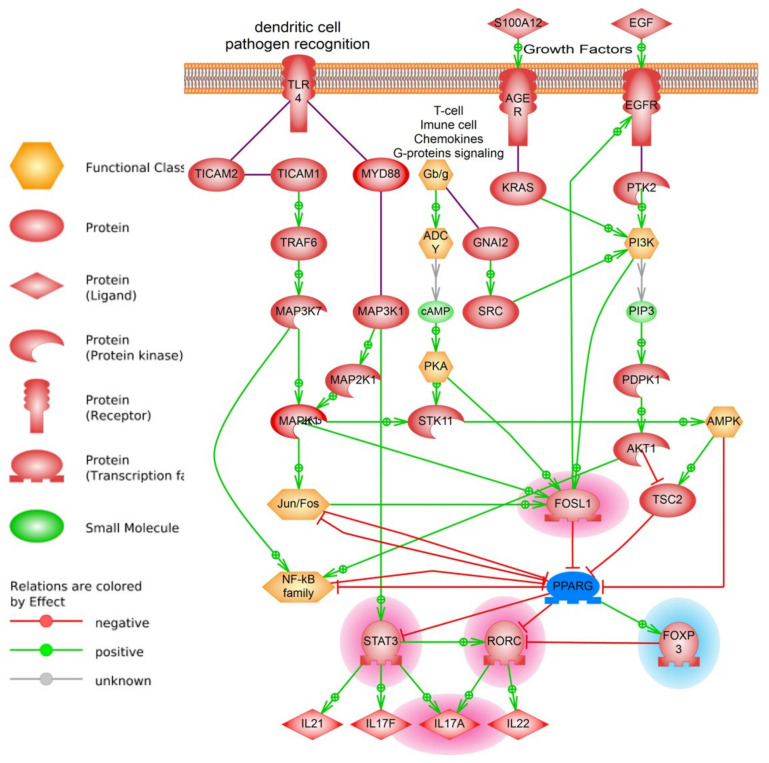
Model of *PPARγ* pathway in psoriasis (simplified version). Changes in gene expression are highlighted according to results of analysis in red (over-expressed genes) and in blue (down-expressed genes). In psoriatic lesion, cytokines, growth factors, pathogens, apoptotic debris as well as dendritic cells activates TLRs, AGER and EGFR among other receptors on the surface of keratinocytes and T-cells. Activates receptors transfer the signal to their canonical cascades such as G-couple proteins, PI3K, PKA, MAPK1, or calcium (not shown). As a result, several direct inhibitors of *PPARG* protein such as TSC2 or inhibitors of *PPARG* expression (*FOSL1*, Jun/Fos) became over activated. When *PPARG* is inhibited on both RNA expression and protein levels, this causes higher than normal expression of interleukins (such as *IL17A*) via *STAT3* and *RORC* transcription factors. See supplemental materials for references and links to publications that support protein-protein interactions in the model.

Several key players according to previously reconstructed models of the *PPARγ* signaling were selected for experimental validation of the hypothesis that low levels of *PPARγ* may contribute to the development of psoriatic lesions. There were *IL17A* gene (interleukin 17A), *STAT3* gene (signal transducer and activator of transcription 3), *RORC* gene (retinoid-related orphan receptor-gamma), *FOXP3* gene (forkhead box P3) and *FOSL1* (FOS-like antigen 1) gene (Figure 7).

We detected the repression of *PPARγ* activity in human psoriatic skin and blood immune cells (CD3^+^ T cells) from 23 patients with real-time PCR method, ELISA and immunohistochemistry analysis. Our results are similar to data from microarray on 58 patients where average *PPARγ* gene expression also was slightly downregulated in psoriatic lesions [18]. Recently, low *PPARγ* expression in CD4^+^ T cells from 12 psoriasis patients than in healthy controls was reported [19]. Other authors however described the higher level of the *PPARγ* expression in human psoriatic skin compared to healthy skin. But the level of *PPARγ* mRNA was close to the detection limit in their research [20].

**Figure 8:**
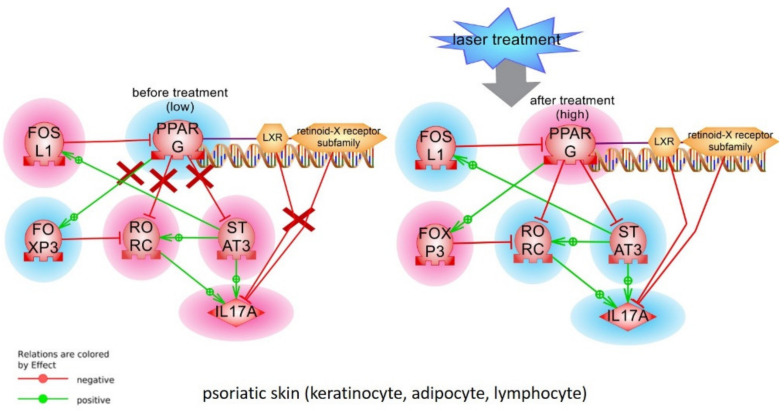
Alignment of tested changes in *PPARγ* signaling before and after laser treatment with the model. Changes in gene expression after treatment are marked with highlights (red—elevated; blue—lowered). Red X symbolise non-functional protein-protein interactions. LXR-RXR complex activity was not tested.

In the model we tested in this work *IL17A*, *STATS3*, and *RORC* are statistically significant negative targets of *PPARγ*. We expected that activity of these targets should be higher in psoriatic lesion and slightly decrease after laser treatment. Our experimental results support this idea and they are aligned well with detected low activity of *PPARγ* in psoriatic skin and CD3^+^ T cells, since *PPARγ* may act as a suppressor of the *IL17* gene transcription by inhibiting his direct transcription factors *RORC* and *STAT3* (Figure 8). In psoriatic cell *STAT3* becomes more active than in healthy cell and, by providing feedback loop via *FOSL1*, further strengthens the downregulation of *PPARγ* expression (Figure 8).

While the prominent role of *RORC* in psoriasis as the major controller of Th17 cell differentiation is well described, however, the evidence of *RORC* expression in psoriasis is controversial. In mice T-cells and dendritic cells had increased *STAT3/RORC* expression [18] and patients with psoriasis had elevated level of *RORC* (RORG-t isoform) [20]. In published microarray data, the level of expression of *RORC* was downregulated in most of 58 patients [15,21].

Contrariwise, *FOSL1* may be important for stabilization of psoriatic inflammation. *FOSL1* was reported to have high level of expression in human psoriasis tissues [22,23] and be able to inhibit *PPARγ* directly [24].

Laser treatment diminishes the *STAT3*->*FOSL1*->*PPARγ* feedback loop and restores *PPARγ* activity and slightly reduce *IL17* production (Figure 8). The molecular mechanism of laser treatment is not well understood. Low-intensity laser waves are absorbed by oxygen, CO_2_, water molecules switching them into an activated state. Proteins with activated molecules participate in interactions more intensively. There was shown that low laser treatment stimulates Ca^2+^-related signaling pathways including general membrane reparation and cell proliferation. There are expectations that low-level laser treatment will result in the replacement of “old” cells with new ones thus reducing the inflammation in the psoriatic lesion [25]. Interesting that ozonated autohemotherapy (OAHT) treatment also elevated *PPARγ* expression in CD4^+^ T cells of patients with psoriasis and decreased patients’ PASI scores [19].

Functions of *IL17* and transcription factors we tested in this work are well studied in psoriasis (see more details in [22,26,27], and in our previous publications [28,29,30]). Other aspects of *PPARγ* related signalling pathways were also studied. For example, interactions between different *PPARs* isoforms are important for their functions. *PPARδ* directly inhibits *PPARγ*, and many pro-inflammatory factors, fatty acid signaling, and “regenerative skin phenotype” pathways (IFNG, TNFA) linked with *PPARδ* stimulation [31]. miRNAs may play important regulator role in *PPARγ* signaling as well [32].

Single nucleotide polymorphisms in *PPARγ* gene are commonly associated with insulin resistance and diabetes. There are no significant associations between mutations in *PPARG* with psoriasis (based on search in OMIM, ClinVAr and Resnet-2020 databases). However, the association between rs1801282 in *PPARγ* and psoriasis, and low level of *PPARγ* expression were reported in Egyptian patients with obesity and metabolic syndrome. Authors concluded that reduced *PPARγ* activity could be the factor responsible for translating the metabolic state among psoriatic patients [33,34].

**Figure 9:**
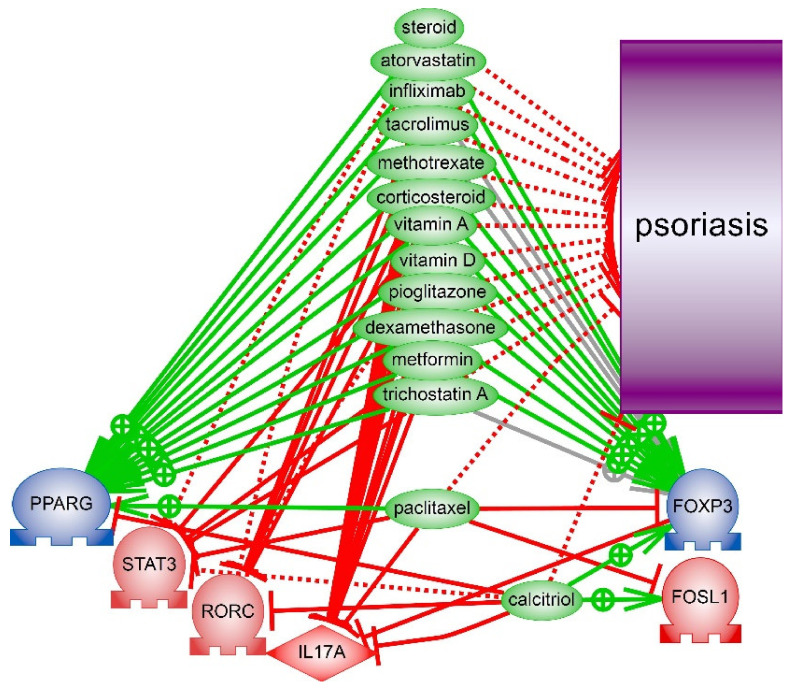
Anti-psoriatic drugs that may stabilize *PPARγ* signaling and supress *IL17* expression. Calcitriol and paclitaxel however have been reported to inhibit *PPARγ* or *FOXP3*. See details of interactions and references in files with models in Supplemental materials.

Can players of *PPARG* signalling be considered as drug targets for psoriasis treatment? We used enrichment analysis with literature biomedical network (Resnet—2020) helps to identify the significant differences in known drugs mechanisms associated with tested model of *PPARγ* signaling. We searched for drugs which were verified in clinical trials or reported in publications as drugs against psoriasis and simultaneously were reported as inhibitors of *IL17*, *STAT3*, *FOSL1*, *RORC* but not *PPARγ* or *FOXP3*. Several substances like corticosteroids and tacrolimus were identified by given criteria. We have found that two other drugs (calcitriol and paclitaxel) that indeed reduce inflammation in psoriasis, however, may not be very effective in psoriasis treatment because they inhibit *PPARγ* or *FOXP3* (Figure 9). Anti-diabetes drugs such as biguanides (metformin) and thiazolidinediones (rosiglitazone and pioglitazone) were studied as additional treatment options for psoriasis. Moreover, it is known that thiazolidinediones act as direct ligand activators of *PPARγ* and it normalizes the histological features of psoriatic skin in vitro [35].

## 5. Conclusions

We detected the high level of *RORC* and *STAT3* mRNA in the psoriatic skin of patients which were reduced after laser treatment. Protein level of *STAT3* also were upregulated in psoriatic skin and CD3^+^ T cells. Also, we report the downregulation of *FOXP3* mRNA expression which is a direct inhibitor of *RORC* and positive target of *PPARγ*. Though, low expression of *PPARγ* as well as high level of *RORC* expression is supported by down-regulated *FOXP3* expression and validates reconstructed model. Experimental data we obtained support our model and the hypothesis that in psoriasis low level of *PPARγ* activity stimulates *IL17* synthesis because *STAT3* and *RORC* became less suppressed.

Our research has several limitations. The number of tested samples was relatively small. Not all proteins from the model were tested on protein levels. Also, additional analysis of receptors activation and intermediate cellular cascades may help to evaluate upstream triggers and regulators of *PPARγ*. Finally, the interaction of transcription factors and regulation of gene expression are more complex than tested model. Additional scaffolds proteins such as LXR-RXR complex, histones and chromatin remodelling complexes are involved in gene expression.

In summary, we report that *PPARγ* weakens the expression of genes that contribute in the development of psoriatic lesion. It is not clear what upstream pathway is the most important for *PPARγ/IL17* regulation in psoriasis. Our data show that transcriptional regulation of *PPARγ* expression by *FOSL1* and by *STAT3*/*FOSL1* feedback loop may be central in the psoriatic skin and T-cells.

## Figures and Tables

**Table 1:** Clinical parameters of patients with psoriasis (*Psoriasis vulgaris*).

Sex, *n* (%)	Age	PASI	Disease History in Years
Patients			
M/F (*n* = 23)	43.5 ± 8.8	22 ± 6.2	17.4 ± 5.7
M, 10 (43.5%)	42.9 ± 9.9	18.4 ± 5.5	15.9 ± 4.7
F, 13 (56.5%)	44.9 ± 9.2	24.8 ± 4.2	17.6 ± 6.7
Healthy volunteers			
M/F (*n* = 10)	40.9 ± 9	*n*/a	*n*/a
M, 4 (40%)	40.2 ± 7.3	*n*/a	*n*/a
F, 6 (60%)	41.3 ± 10.6	*n*/a	*n*/a

**Table 2:** Comparative analysis of gene and protein expression in male (*n* = 10) and female (*n* = 13) patients.

Group	*FOSL1*	*PPARγ*	*FOXP3*	*STAT3*	*IL17A*	*RORC*
(a) qPCR of skin samples, folds;						
males	7.36 ± 3.50	0.66 ± 0.06	0.61 ± 0.07	4.52 ± 0.66	31.27 ± 7.42	6.87 ± 1.26
females	12.29 ± 3.73	0.74 ± 0.10	0.56 ± 0.06	4.39 ± 0.69	47.93 ± 14.02	7.78 ± 1.02
*p*	0.36	0.52	0.59	0.90	0.35	0.58
(b) qPCR of CD3^+^ cells, folds;						
males	5.80 ± 1.69	0.27 ± 0.15	0.22 ± 0.06	9.83 ± 3.94	123.99 ± 20.38	13.84 ± 3.65
females	5.79 ± 1.24	0.31 ± 0.13	0.16 ± 0.04	4.79 ± 1.62	90.82 ± 10.69	16.82 ± 2.73
*p*	1.00	0.86	0.38	0.21	0.14	0.51
(c) ELISA of skin samples, ng/mL;						
males	N.D.	1.94 ± 0.30	N.D.	7.20 ± 1.01	1.23 ± 0.15	N.D.
females	N.D.	2.16 ± 0.29	N.D.	8.46 ± 0.78	1.08 ± 0.12	N.D.
*p*		0.61		0.33	0.45	
(d) ELISA of CD3^+^, ng/mL;						
males	N.D.	2.05 ± 0.55	N.D.	14.33 ± 2.49	1.50 ± 0.13	N.D.
females	N.D.	1.46 ± 0.32	N.D.	10.82 ± 1.97	1.63 ± 0.13	N.D.
*p*		0.33		0.27	0.49	

**Table 3:** Comparative analysis of gene and protein expression in female patients have (*n* = 6) and do not have (*n* = 7) menopause.

Group	*FOSL1*	*PPARγ*	*FOXP3*	*STAT3*	*IL17A*	*RORC*
(a) qPCR of skin samples, folds;						
no menopause	7.15 ± 1.39	0.76 ± 0.12	0.57 ± 0.10	4.79 ± 1.10	43.86 ± 10.54	8.71 ± 1.37
menopause	16.69 ± 7.52	0.73 ± 0.16	0.52 ± 0.07	3.83 ± 0.71	59.23 ± 28.35	7.84 ± 1.93
*p*	0.21	0.88	0.70	0.41	0.60	0.71
(b) qPCR of CD3^+^ cells, folds;						
no menopause	8.04 ± 1.87	0.24 ± 0.14	0.14 ± 0.04	7.07 ± 2.77	82.59 ± 14.88	16.98 ± 4.68
menopause	3.17 ± 0.75	0.39 ± 0.24	0.18 ± 0.08	2.12 ± 0.60	100.43 ± 15.80	16.64 ± 2.85
*p*	**0.047**	0.59	0.61	0.13	0.43	0.95
(c) ELISA of skin samples, ng/mL;						
no menopause	N.D.	2.45 ± 0.46	N.D.	7.69 ± 0.49	0.98 ± 0.16	N.D.
menopause	N.D.	1.81 ± 0.30	N.D.	9.36 ± 1.58	1.21 ± 0.18	N.D.
*p*		0.29		0.30	0.36	
(d) ELISA of CD3^+^, ng/mL;						
no menopause	N.D.	1.80 ± 0.42	N.D.	11.41 ± 2.87	1.55 ± 0.21	N.D.
menopause	N.D.	1.05 ± 0.48	N.D.	10.13 ± 2.90	1.72 ± 0.15	N.D.
*p*		0.26		0.76	0.55	

**Table 4:** Comparative analysis of gene and protein expression in healthy male (*n* = 4) and female (*n* = 6) volunteers.

Group	*FOSL1*	*PPARγ*	*FOXP3*	*STAT3*	*IL17A*	*RORC*
(a) qPCR of CD3^+^ cells, folds;						
males	1.23 ± 0.52	1.34 ± 0.65	1.26 ± 0.29	1.02 ± 0.55	0.91 ± 0.38	0.47 ± 0.14
females	0.85 ± 0.18	0.77 ± 0.13	0.83 ± 0.16	0.99 ± 0.24	1.06 ± 0.23	1.35 ± 0.46
*p*	0.44	0.41	0.20	0.96	0.73	0.17
(b) ELISA of skin samples, ng/mL;						
males	N.D.	9.16 ± 1.64	N.D.	3.95 ± 0.73	0.04 ± 0.01	N.D.
females	N.D.	7.27 ± 0.77	N.D.	3.89 ± 1.15	0.07 ± 0.01	N.D.
*p*		0.27		0.97	**0.049**	
(c) ELISA of CD3^+^, ng/mL;						
males	N.D.	13.93 ± 1.37	N.D.	6.45 ± 2.09	0.05 ± 0.02	N.D.
females	N.D.	11.47 ± 2.35	N.D.	5.28 ± 0.89	0.09 ± 0.03	N.D.
*p*		0.46		0.57	0.29	

## Data Availability

Not applicable.

## Supplementary Materials

The following are available online at https://www.mdpi.com/article/10.3390/ijms22168603/s1.
