# Supplementary material for: Analysis of PPARγ Signaling Activity in Psoriasis
Source: Int J Mol Sci. 2021 Aug 10;22(16):8603. doi: 10.3390/ijms22168603 (PMC8395241; doi:10.3390/ijms22168603)
Supplement: Supplementary file 1 [file ijms-22-08603-s001.zip › Supplemental materials_Analysis of PPARg signaling activity in psoriasis/Pathway models/2683_PS_QuickStartGuide_2020.pdf]

Pathway Studio ® Mammal Plus

Entity Types

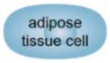	**Cell** – Mammal cell types and cell lines
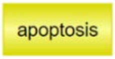	**Cell Process*** – biological processes, most coincide with Gene Ontology
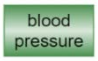	**Clinical Parameter** – measured parameters of the human body used in clinical practice
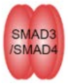	**Complex*** – several polypeptides that form a complex via physical interactions
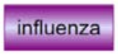	**Disease** – health conditions and disease terms from MeSH and Emtree
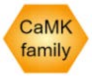	**Functional Class*** – most functional classes coincide with Gene Ontology
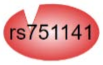	**Genetic Variant** – Text mining and imported from ClinVar (both gene level and phenotype association)
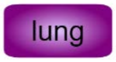	**Organ –** mammal organ types
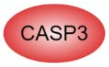	**Protein** – defined by Entrez Gene - represents both genes and the gene products, including proteins and miRNAs
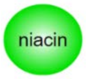	**Small Molecule** – naturally occurring metabolites and small molecules found in cells as well as drugs (including some biologically active polypeptides such as monoclonal antibodies)
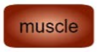	**Tissue** - mammal tissue types
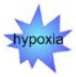	**Treatment** – non-chemical treatments and environmental conditions, such as cold shock

(complex entities)*

Relationship Types

GENE EXPRESSION

**Expression**	Regulator changes protein abundance by affecting levels of transcript or protein stability.
**miRNAEffect**	The inhibitory effect of a miRNA on its mRNA target
**PromoterBinding**	A regulator that binds to the promoter of a gene

REGULATION *(less specific than other relation types)*

**Regulation**	Changes the activity of the target by an unknown mechanism (may be direct or indirect). This is a less specific relation type than others provided.

CELL SURFACE

**Cell Expression**	Expression of Proteins within or on the surface of a Cell<br>Filtering Field* Name: **Mechanism**<br>Sub-Categories: Surface

DISEASE / CELL PROCESS

**Biomarkers**	Identification of proteins / complexes / functional classes / metabolites that are prognostic or diagnostic biomarkers for a disease (between disease-protein / complex / functional class / naturally occurring small molecules)<br>Filtering Field* Name: **Biomarker Type**<br>Sub-Categories: Diagnostic, Prognostic
**Clinical Trials**	Disease/cell process relationship representing clinical trials conducted for a drug against a disease (from ClinicalTrials.gov) (between Disease / Cell Process – Small Molecule) (no sub-types)
**Functional Association**	Different types of functional associations between a disease and a cellular process or another disease (between Disease – Cell Process) (no sub-types)
**Genetic Change**	Genetic changes in a gene in a disease state such as gene deletions, amplifications, mutations or epigenetic changes (between disease-protein /complex / functional class)<br>Filtering Field* Name: **Change Type**<br>Sub-Categories: Alternative splicing, Gene Deletion, Mutation, Gene Amplification, Epigenic Methylation, Phosphorylation
**Quantitative Change**	Changes in abundance / activity / expression of a gene / protein / small molecule in a disease state (between disease-protein / complex / functional class /small molecules)<br>Filtering Field* Name: **Quantitative Type**<br>Sub-Categories: Expression, Abundance, Activity, Secretion
**State Change**	Changes in a protein's post-translational modification status or alternative splicing events associated with a disease (between disease-protein / complex functional class)<br>Filtering Field* Name: **Change Type**<br>Sub-Categories: Alternate Splicing, Phosphorylation, Epigenic methylation, Gene amplification, Gene deletion, Mutation

PROTEOMICS/PHYSICAL INTERACTIONS
(excluding promoter binding and miRNA regulation)

**Binding**	Direct physical interaction between two molecules
**DirectRegulation**	Influences target activity by direct physical interaction<br>(excluding promoter binding interactions)
**ProtModification**	A regulator that changes the modification of the target molecule, usually by a direct interaction<br>Filtering Field* Name: **Mechanism**<br>Sub-Categories: Acetylation, Cleavage, Deacetylation, Degradation, Demethylation, Dephosphorylation, Desumoylation, Export, Deubiquitination, Direct interaction, Methylation, Phosphorylation, Posttranscriptional Inhibition, Proteolysis, Sumoylation, Surface, Ubiquitination

Finding subtypes of relations by using the filtering fields*

Example: Genetic Change: change type = mutation

Select "Add Condition" to apply filters to entities or relations in the Advanced Network Builder filter

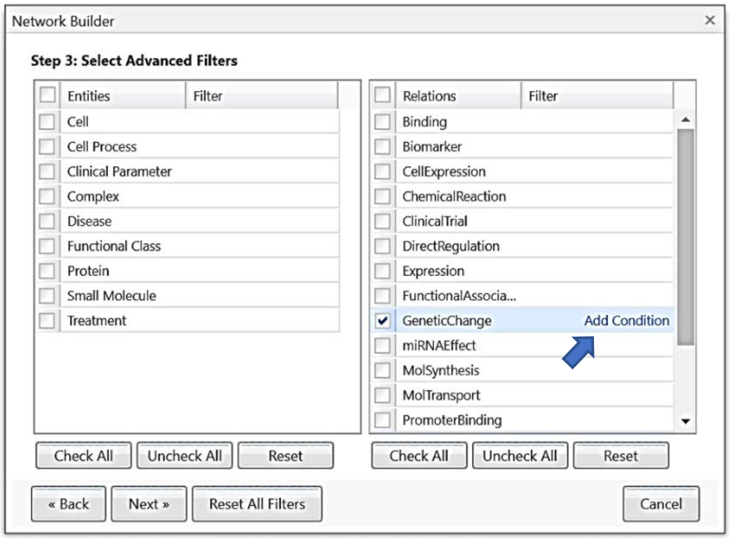

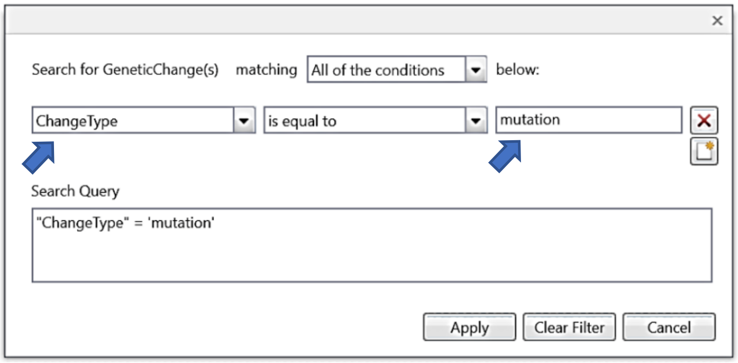

Relation directionality and effect

All relations have arrows to indicate directionality except Binding, CellExpression and Functional Association, which have no directionality.

Effect can be observed by the type of arrow head, and as shown here can be colored by effect (Style>Color Relations>By Type)

Positive effect

Negative effect

No effect identified

No directionality (Functional Association, Binding and CellExpression only)

Relations colored by type

Protein Classes

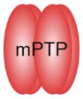

Complexes are also "protein" entities but represent a group of proteins functioning together. In the Pathway Studio database they function as a complex entity type so are considered separately.

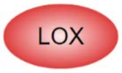

Protein (no class assigned)

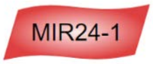

Non-protein coding RNA (miRNA)

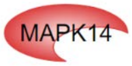

Kinase

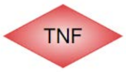

Ligand

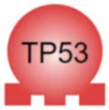

Transcription factor

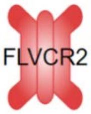

Transporter

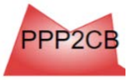

Phosphatase

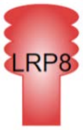

Receptor

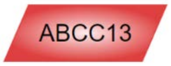

Chromosomal aberration gene, chromosomal fragile site, DNA segment gene, or pseudogene

Building Pathway Options

Relations between Selected and Unselected – finds direct relationship between selected entities and the rest of the entities on the network diagram.

Tools within the Network Builder:

Shortest Path – finds relationships between two selected entities on the network diagram, adding intermediate entities as needed to form the connection.

Expand Pathway – finds entities directly connected to the entity or Entities selected on the network diagram from the database.

Common Targets – finds target(s) that are regulated by at least two or more of the selected entities on the network diagram.

Common Regulators – finds regulator(s) that regulate two or more of the selected entities on the network.

Common Binding Partners – finds entities that bind two or more of the selected entities in a network.

PATHWAY STUDIO is a registered trademark of Elsevier Inc. Copyright© 2020 Elsevier B.V. All rights reserved.
