# Supplementary material for: Analysis of PPARγ Signaling Activity in Psoriasis
Source: Int J Mol Sci. 2021 Aug 10;22(16):8603. doi: 10.3390/ijms22168603 (PMC8395241; doi:10.3390/ijms22168603)
Supplement: Supplementary file 1 [file ijms-22-08603-s001.zip › Supplemental materials_Analysis of PPARg signaling activity in psoriasis/Pathway models/Models images and html files/Anti-psoriatic drugs influence PPARG signaling/10002.html]

RORC


# Protein RORC

|  |  |
| --- | --- |
| URN | urn:agi-llid:6097 |
| Total Entities | 0 |
| Connectivity | 7054 |
| Name | RORC |
| Description | RAR related orphan receptor C |
| Notes | The protein encoded by this gene is a DNA-binding transcription factor and is a member of the NR1 subfamily of nuclear hormone receptors. The specific functions of this protein are not known; however, studies of a similar gene in mice have shown that this gene may be essential for lymphoid organogenesis and may play an important regulatory role in thymopoiesis. In addition, studies in mice suggest that the protein encoded by this gene may inhibit the expression of Fas ligand and IL2. Two transcript variants encoding different isoforms have been found for this gene. [provided by RefSeq, Jul 2008] |
| Primary Cell Localization | Nucleus |
| Class | Transcription factor |

---

|  |  |
| --- | --- |
| Pathway | T-Cell Positive Selection and Neglect Induced Death |
|  | Kynurenine/AHR Signaling in Treg Cell Activation |
|  | AHR Signaling in Th17 Cells Function |
|  | Th17-Cell Differentiation |
|  | AHR Signaling in Tr1 Cells Function |
|  | AHR in Intestinal Cell Antimicrobial Barrier Maintenance |
|  | Lymphocyte Mediated Myocardial Injury in Myocarditis |
|  | Proteins Involved in Myocarditis |
|  | T-Cells Differentiation Block in Psoriasis |
|  | Proteins with Altered Expression in Psoriasis |
|  | Th17-Cell Function in Systemic Lupus Erythematosus |
|  | Th17-Cell Activation in Crohn's Disease |
|  | Th17-Cell Differentiation in Asthma |
|  | Th17-Cell and Th1 Immune Responsein Psoriatic Arthritis |
|  | Th17-Cell Numbers Reduction in HIV |
|  | IL27R -> STAT Signaling |
|  | TNF Receptors -> Expression Targets in Lymphoid System and Blood |
|  | Interleukin Receptors -> Expression Targets in Lymphoid System and Blood |
|  | Chemokine Receptor Family -> Expression Targets in Lymphoid System |
|  | PPAR Psoriasis |
|  | prarg neg,ukn expres targets, ps-positive |
|  | PPARG negative targets |
|  | pparg expr target\_regulators |
|  | Neighbors of disease exacerbation |
|  | Neighbors of cancer progression |
|  | Neighbors of disease severity |
|  | Neighbors of mortality |
|  | Neighbors of insulin sensitivity |
|  | Neighbors of tumor response |
|  | Neighbors of body weight gain |
|  | Neighbors of glucose tolerance |
|  | Neighbors of blood glucose |
|  | Neighbors of T-cell count |
|  | Neighbors of disease exacerbation |
|  | Neighbors of cancer progression |
|  | Neighbors of disease severity |
|  | Neighbors of mortality |
|  | Neighbors of insulin sensitivity |
|  | Neighbors of tumor response |
|  | Neighbors of body weight gain |
|  | Neighbors of glucose tolerance |
|  | Neighbors of blood glucose |
|  | Neighbors of T-cell count |
|  | Neighbors of cell development |
|  | Neighbors of immune response |
|  | Neighbors of cell population |
|  | Neighbors of T-cell development |
|  | Neighbors of inflammatory response |
|  | Neighbors of cell function |
|  | Neighbors of cell formation |
|  | Neighbors of adaptive immune response |
|  | Neighbors of osteoclast development |
|  | Neighbors of epithelial to mesenchymal transition |
|  | Neighbors of immunity |
|  | Neighbors of chemotaxis |
|  | Neighbors of cell survival |
|  | Neighbors of T-cell proliferation |
|  | Neighbors of T-cell activation |
|  | Neighbors of tumor growth |
|  | Neighbors of cell count |
|  | Neighbors of adipogenesis |
|  | Neighbors of cell damage |
|  | Neighbors of aging |
|  | Neighbors of adipocyte differentiation |
|  | Neighbors of regeneration |
|  | Neighbors of cell migration |
|  | Neighbors of cancer growth |
|  | Neighbors of T-cell function |
|  | Neighbors of immune system function |
|  | Neighbors of transcription activation |
|  | Neighbors of T-cell response |
|  | Neighbors of tumor immunity |
|  | Neighbors of cell transdifferentiation |
|  | Neighbors of cell growth |
|  | Neighbors of ROS generation |
|  | Neighbors of cancer cell growth |
|  | Neighbors of sensitization |
|  | Neighbors of cellular immune response |
|  | Neighbors of cell differentiation |
|  | Neighbors of life span |
|  | Neighbors of cell homeostasis |
|  | Neighbors of psoriasis |
|  | Neighbors of inflammatory disease |
|  | Neighbors of colitis |
|  | Neighbors of arthritis |
|  | Neighbors of inflammatory bowel disease |
|  | Neighbors of metastasis |
|  | Neighbors of multiple sclerosis |
|  | Neighbors of chronic inflammation |
|  | Neighbors of inflammation |
|  | Neighbors of tumor microenvironment |
|  | Neighbors of obesity |
|  | Neighbors of asthma |
|  | Neighbors of pneumonia |
|  | Neighbors of liver fibrosis |
|  | Neighbors of chronic obstructive pulmonary disease |
|  | Neighbors of leukocyte infiltration |
|  | Neighbors of reperfusion injury |
|  | Neighbors of airway inflammation |
|  | Neighbors of insulin resistance |
|  | Neighbors of infection |
|  | Neighbors of preeclampsia |
|  | Neighbors of death |
|  | Neighbors of encephalomyelitis |
|  | Neighbors of autoimmune disease |
|  | Neighbors of dermatitis |
|  | Neighbors of autoimmunity |
|  | Neighbors of experimental autoimmune encephalomyelitis |
|  | Neighbors of breast cancer |
|  | Neighbors of systemic lupus erythematosus |
|  | Neighbors of stroke |
|  | Neighbors of neoplasm |
|  | Neighbors of respiratory hypersensitivity |
|  | Neighbors of pancreatic cancer |
|  | Neighbors of macrophage |
|  | Neighbors of PBMC |
|  | Neighbors of neutrophil |
|  | Neighbors of immunocompetent cell |
|  | Neighbors of NF-kB family |
|  | Neighbors of IL1 family |
|  | Neighbors of PI3K |
|  | Neighbors of STAT family |
|  | Neighbors of IFNAR ligand |
|  | Neighbors of transforming growth factor |
|  | Neighbors of cytokine |
|  | Neighbors of interferon |
|  | Neighbors of interleukin |
|  | Neighbors of NF-AT family |
|  | Neighbors of IgG |
|  | Neighbors of SMAD subfamily |
|  | Neighbors of LXR |
|  | Neighbors of inflammatory cytokine |
|  | Neighbors of IL23 |
|  | Neighbors of estrogen receptor |
|  | Neighbors of T-cell receptor |
|  | Neighbors of histone deacetylase |
|  | Neighbors of IL1R |
|  | Neighbors of HIF-1 |
|  | Neighbors of IL12 |
|  | Neighbors of Notch |
|  | Neighbors of interleukin-35 |
|  | Neighbors of class nuclear receptor with C4 zinc fingers |
|  | Neighbors of non-coding RNA |
|  | Neighbors of histone H3 |
|  | Neighbors of STAT5 |
|  | Neighbors of retinoid-X receptor subfamily |
|  | Neighbors of immunoglobulin |
|  | Neighbors of AHR subfamily |
|  | Neighbors of RA receptor |
|  | Model of PPARG signaling in psoriasis |
|  | PPARG negative regulators and targets |
|  | Model of PPARG related pathways in psoriasis (short version) |
|  | New Pathway (5) |
|  | 1\_Differentiation of psoriatic T cells |
|  | Model of PPARG signaling in psoriais (tested) |
|  | before laser treatment |
|  | Differentiation of psoriatic T cells |
|  | Anti-psoriatic drugs influence PPARG signaling |
|  | PPARG signaling after laser treatment |

---

|  |  |
| --- | --- |
| MedScan ID | 6097 |

---

|  |  |
| --- | --- |
| LocusLink ID | 6097 |
|  | 19885 |
|  | 102556141 |

---

|  |  |
| --- | --- |
| Alias | RORC |
|  | IMD42 locus |
|  | OTTMUSP00000025974 |
|  | RZRG |
|  | MGC129539 |
|  | thymus orphan receptor |
|  | OTTMUSP00000025973 |
|  | RORG |
|  | thymus orphan receptors |
|  | RAR-related orphan receptor gamma |
|  | nuclear receptor subfamily 1 group F member 3 |
|  | RAR-related orphan receptor C |
|  | nuclear receptor ROR-gamma |
|  | OTTHUMP00000015253 |
|  | nuclear receptor ROR-gamma-like |
|  | retinoid-related orphan receptor gamma |
|  | RP11-98D18.11-001 |
|  | NR1F3 |
|  | retinoic acid-binding receptor gamma |
|  | RAR-related orphan nuclear receptor variant 2 |
|  | RAR-related orphan receptor C, isoform a |
|  | RORgamma |
|  | RZR-gamma |
|  | nuclear receptor NR1F3 |
|  | RORgammat |
|  | Nuclear receptor RZR-gamma |
|  | LOC102556141 |
|  | RP11-98D18.11 |
|  | MGC189525 |
|  | transcription factor NR1F3 |
|  | TOR |
|  | IMD42 |
|  | Thor |
|  | retinoid-related orphan receptor-gamma |

---

|  |  |
| --- | --- |
| GO ID | 0003700 |
|  | 0000981 |
|  | 0001227 |
|  | 0000978 |
|  | 0004879 |
|  | 0008142 |
|  | 0003707 |
|  | 0001223 |
|  | 0098531 |
|  | 0008270 |
|  | 0072539 |
|  | 0060612 |
|  | 0036315 |
|  | 0032922 |
|  | 0019221 |
|  | 0030522 |
|  | 0000122 |
|  | 0042753 |
|  | 0045893 |
|  | 0045598 |
|  | 0010906 |
|  | 0019218 |
|  | 0060850 |
|  | 0043401 |
|  | 0006367 |
|  | 0006805 |
|  | 0016604 |
|  | 0005654 |
|  | 0005634 |
|  | 0003677 |
|  | 0046872 |
|  | 0043565 |
|  | 0048541 |
|  | 0033077 |
|  | 0042093 |
|  | 0046632 |
|  | 0030154 |
|  | 0032620 |
|  | 0072615 |
|  | 0048535 |
|  | 0048537 |
|  | 0007275 |
|  | 0070244 |
|  | 0006468 |
|  | 0045586 |
|  | 0006355 |
|  | 0048511 |
|  | 0009897 |
|  | 0006351 |
|  | 0001078 |
|  | 0043231 |
|  | 0005730 |
|  | 0010467 |

---

|  |  |
| --- | --- |
| KEGG ID | hsa:6097 |
|  | mmu:19885 |

---

|  |  |
| --- | --- |
| Organism | Homo sapiens {Organism urn:agi-taxid:9606} |
|  | Mus musculus {Organism urn:agi-taxid:10090} |
|  | Mus musculus |
|  | Homo sapiens |
|  | Rattus norvegicus |

---

|  |  |
| --- | --- |
| Mouse chromosome position | 3 |

---

|  |  |
| --- | --- |
| OMIM ID | 602943 |
|  | 616622 |

---

|  |  |
| --- | --- |
| Rat chromosome position | 2 |

---

|  |  |
| --- | --- |
| Hugo ID | 10260 |
|  | HGNC:10260 |

---

|  |  |
| --- | --- |
| Human chromosome position | 1q21.3 |
|  | 1q21 |

---

|  |  |
| --- | --- |
| Swiss-Prot Accession | F1D8P6 |
|  | P51449 |
|  | Q6I9R9 |
|  | P51449.2 |
|  | A0A0G2JGZ6 |
|  | P51450 |
|  | A0A0R4J096 |
|  | P51450.1 |
|  | Q5SZR9 |
|  | Q8N5V7 |
|  | Q8NCY8 |
|  | E9Q8I1 |
|  | Q3U513 |
|  | Q61027 |
|  | Q91YT5 |
|  | Q9QXD9 |
|  | Q9R177 |

---

|  |  |
| --- | --- |
| PIR ID | JC2494 |
|  | JC5375 |

---

|  |  |
| --- | --- |
| GenBank ID | NC\_000001 |
|  | XM\_006711484 |
|  | XP\_006711547 |
|  | NM\_005060 |
|  | NP\_005051 |
|  | NM\_001001523 |
|  | NP\_001001523 |
|  | NG\_029118 |
|  | AL589765 |
|  | CH471121 |
|  | EAW53405 |
|  | EAW53406 |
|  | KT583857 |
|  | AB307696 |
|  | BAH02287 |
|  | AF075096 |
|  | AK097994 |
|  | BAG53561 |
|  | AK128522 |
|  | AK223137 |
|  | BAD96857 |
|  | AK298460 |
|  | BAG60673 |
|  | AK315662 |
|  | BAG38028 |
|  | AL834219 |
|  | CAD38900 |
|  | AW004872 |
|  | BC031554 |
|  | AAH31554 |
|  | BC110571 |
|  | AAI10572 |
|  | BI520481 |
|  | CB161467 |
|  | CR457436 |
|  | CAG33717 |
|  | DB148892 |
|  | HQ692834 |
|  | ADZ17345 |
|  | KY670636 |
|  | AUT36352 |
|  | U16997 |
|  | AAA64751 |
|  | P51449 |
|  | NC\_000069 |
|  | XM\_006501164 |
|  | XP\_006501227 |
|  | XM\_006501163 |
|  | XP\_006501226 |
|  | NM\_011281 |
|  | NP\_035411 |
|  | NR\_121656 |
|  | NM\_001293734 |
|  | NP\_001280663 |
|  | XM\_006501162 |
|  | XP\_006501225 |
|  | AC164562 |
|  | AH006790 |
|  | AAC53501 |
|  | AJ131723 |
|  | CAA10488 |
|  | CH466620 |
|  | EDL38724 |
|  | EDL38725 |
|  | AF163668 |
|  | AAD46913 |
|  | AJ132394 |
|  | CAA10661 |
|  | AK137866 |
|  | BAE23510 |
|  | AK153941 |
|  | BAE32267 |
|  | AK160122 |
|  | BAE35645 |
|  | BC014804 |
|  | AAH14804 |
|  | BF019665 |
|  | CJ098183 |
|  | U39071 |
|  | AAB02582 |
|  | U43508 |
|  | AAB40709 |
|  | P51450 |
|  | XR\_426792 |
|  | NC\_018912 |
|  | AMYH02001889 |
|  | AM393818 |
|  | CAL38693 |
|  | AM393876 |
|  | CAL38751 |
|  | DQ890805 |
|  | ABM81731 |
|  | DQ893960 |
|  | ABM84886 |
|  | AC\_000025 |
|  | AAHY01029210 |
|  | AAHY01029211 |
|  | AAHY01029212 |
|  | AAHY01029213 |
|  | AF019655 |
|  | AF019656 |
|  | AF019657 |
|  | AF019658 |
|  | AF019659 |
|  | AF019660 |
|  | XM\_005245424 |
|  | XP\_005245481 |
|  | AC\_000133 |
|  | ABBA01049405 |
|  | XM\_006501161 |
|  | XP\_006501224 |
|  | XR\_375502 |

---

|  |  |
| --- | --- |
| Swiss-Prot ID | RORG\_HUMAN |
|  | RORG\_MOUSE |

---

|  |  |
| --- | --- |
| Cell Localization | Nucleus |

---

|  |  |
| --- | --- |
| Ensembl ID | ENSG00000143365 |
|  | ENSP00000349164.6 |
|  | ENST00000356728.10 |
|  | ENSP00000327025.6 |
|  | ENST00000318247.7 |
|  | ENSMUSG00000028150 |
|  | ENSMUSP00000143763.1 |
|  | ENSMUST00000197040.4 |
|  | ENSMUSP00000029795.3 |
|  | ENSMUST00000029795.9 |
|  | ENST00000318247.6 |
|  | ENSP00000349164 |
|  | ENST00000356728 |
|  | ENSP00000327025 |
|  | ENST00000318247 |
|  | ENSMUSP00000143763 |
|  | ENSMUST00000197040 |
|  | ENSMUSP00000029795 |
|  | ENSMUST00000029795 |

---

|  |  |
| --- | --- |
| MGI ID | MGI:104856 |

---

|  |  |
| --- | --- |
| RGD ID | 7523591 |

---

|  |  |
| --- | --- |
| Unigene ID | Hs.607993 |
|  | Mm.4372 |
|  | Hs.256022 |

---

|  |  |
| --- | --- |
| Homologene ID | 21051 |

---

|  |  |
| --- | --- |
| Shape | Stick-vertex |

---
