# Supplementary material for: Analysis of PPARγ Signaling Activity in Psoriasis
Source: Int J Mol Sci. 2021 Aug 10;22(16):8603. doi: 10.3390/ijms22168603 (PMC8395241; doi:10.3390/ijms22168603)
Supplement: Supplementary file 1 [file ijms-22-08603-s001.zip › Supplemental materials_Analysis of PPARg signaling activity in psoriasis/Pathway models/Models images and html files/Anti-psoriatic drugs influence PPARG signaling/1001638915.html]

pioglitazone ---| IL17A


# Expression pioglitazone ---| IL17A

|  |  |
| --- | --- |
| URN | urn:agi-expression:in-out:urn:agi-cas:105355-27-9:out:urn:agi-llid:3605:eff=negative |
| Connectivity | 2 |
| References | 8 |
| Effect | negative |

---
