# Supplementary material for: Analysis of PPARγ Signaling Activity in Psoriasis
Source: Int J Mol Sci. 2021 Aug 10;22(16):8603. doi: 10.3390/ijms22168603 (PMC8395241; doi:10.3390/ijms22168603)
Supplement: Supplementary file 1 [file ijms-22-08603-s001.zip › Supplemental materials_Analysis of PPARg signaling activity in psoriasis/Pathway models/Models images and html files/Anti-psoriatic drugs influence PPARG signaling/100854.html]

infliximab


# Small Molecule infliximab

|  |  |
| --- | --- |
| URN | urn:agi-cas:170277-31-3 |
| Total Entities | 0 |
| Connectivity | 1941 |
| Name | infliximab |

---

|  |  |
| --- | --- |
| Pathway | Dendritic Cell Dysfunction in Crohn's disease |
|  | Anti-psoriatic drugs influence PPARG signaling |

---

|  |  |
| --- | --- |
| MedScan ID | 1285073 |

---

|  |  |
| --- | --- |
| Alias | Zessly |
|  | Revellex |
|  | PF 6438179 |
|  | infliximab-abda |
|  | GP 1111 |
|  | Flixabi |
|  | TA-650 |
|  | Avakine |
|  | Remsima |
|  | 170277-31-3 |
|  | HSDB 7850 |
|  | CT-P13 |
|  | infliximab-qbtx |
|  | CenTNF |
|  | Ixifi |
|  | PF 06438179 |
|  | Remicade |
|  | Renflexis |
|  | Inflectra |
|  | infliximab-dyyb |
|  | infliximab |

---

|  |  |
| --- | --- |
| CAS ID | 170277-31-3 |

---

|  |  |
| --- | --- |
| PharmaPendium ID | Infliximab |

---
