# Supplementary material for: Analysis of PPARγ Signaling Activity in Psoriasis
Source: Int J Mol Sci. 2021 Aug 10;22(16):8603. doi: 10.3390/ijms22168603 (PMC8395241; doi:10.3390/ijms22168603)
Supplement: Supplementary file 1 [file ijms-22-08603-s001.zip › Supplemental materials_Analysis of PPARg signaling activity in psoriasis/Pathway models/Models images and html files/Anti-psoriatic drugs influence PPARG signaling/10997.html]

STAT3


# Protein STAT3

|  |  |
| --- | --- |
| URN | urn:agi-llid:6774 |
| Total Entities | 0 |
| Connectivity | 11402 |
| Name | STAT3 |
| Description | signal transducer and activator of transcription 3 (acute-phase response factor) |
| Notes | The protein encoded by this gene is a member of the STAT protein family. In response to cytokines and growth factors, STAT family members are phosphorylated by the receptor associated kinases, and then form homo- or heterodimers that translocate to the cell nucleus where they act as transcription activators. This protein is activated through phosphorylation in response to various cytokines and growth factors including IFNs, EGF, IL5, IL6, HGF, LIF and BMP2. This protein mediates the expression of a variety of genes in response to cell stimuli, and thus plays a key role in many cellular processes such as cell growth and apoptosis. The small GTPase Rac1 has been shown to bind and regulate the activity of this protein. PIAS3 protein is a specific inhibitor of this protein. Mutations in this gene are associated with infantile-onset multisystem autoimmune disease and hyper-immunoglobulin E syndrome. Alternative splicing results in multiple transcript variants encoding distinct isoforms. [provided by RefSeq, Sep 2015] |
| Primary Cell Localization | Nucleus |
| Class | Transcription factor |
| ObjectType | Protein |

---

|  |  |
| --- | --- |
| Pathway | Proteins Involved in Stem Cell Exhaustion in Aging |
|  | Apoptosis of Cochlear Hair Cells (Noise-Induced, Mouse Model) |
|  | mTOR Signaling |
|  | Telomer Attrition Triggers Genomic Instability |
|  | Leptin and CCK8 Activate Nodose Ganglia Neuron |
|  | P-cell Activation |
|  | I-cell: CCK-8 Secretion and Regulation of Eating Behavior |
|  | N-cell: Neurotensin Production |
|  | Serotonin Receptors Signaling |
|  | PRL and CSH Action in Mammary Gland |
|  | Thrombopoietin Receptors Signaling in Platelet Maturation |
|  | T-Cell Maturation (Hypothesis) |
|  | AHR Signaling in Th17 Cells Function |
|  | Th17-Cell Differentiation |
|  | AHR Signaling in Tr1 Cells Function |
|  | CXC Chemokine Receptor Signaling |
|  | CC Chemokine Receptor Signaling |
|  | Plasmin Effects in Inflammation |
|  | Proplatelet Maturation |
|  | AHR in Intestinal Cell Antimicrobial Barrier Maintenance |
|  | Basophil Activation |
|  | Eosinophil Survival by Cytokine Signaling |
|  | Mast-Cell Activation via IgE Signaling |
|  | Mast-Cell Activation without Degranulation through CXCR4 Signaling |
|  | Mast-Cell Activation without Degranulation |
|  | Macrophage M1 Lineage |
|  | CD16/CD14 Proinflammatory Monocyte Activation |
|  | anti-Inflammatory Function of Macrophage M2 Lineage |
|  | Neutrophil Recruitment and Priming |
|  | Negative Acute Phase Proteins Synthesis |
|  | Positive Acute Phase Proteins Synthesis |
|  | Vascular Endothelial Cell Activation by Cytokines |
|  | Vascular Endothelial Cell Activation by Blood Coagulation Factors |
|  | Adipokines Production by Adipocyte |
|  | Nociception Expression Targets Signaling |
|  | CCR1 Expression Targets |
|  | ADRA1A -> IL6 Production |
|  | PTGER2/3 -> Inflammation-Related Expression Targets |
|  | IL6 Expression Targets -> Nociception |
|  | Leptin -> CD25/IL6/IL10 Production |
|  | CHRNA7 -> IL8 Production |
|  | Proteins Involved in Atherosclerosis |
|  | Proteins Involved in Dilated Cardiomyopathy |
|  | Cardiomyocyte Hypertrophy |
|  | Hypertrophic Cardiomyopathy |
|  | Proteins Involved in Arterial Hypertension |
|  | TRPM4/6/7/8 Signaling in Arterial Hypertension (Hypothesis) |
|  | Proteins Involved in Pulmonary Hypertension |
|  | Endothelial Cell Dysfunction in Pulmonary Hypertension |
|  | Proteins Involved in Myocardial Ischemia |
|  | Preconditioning Ischemia |
|  | Lymphocyte Mediated Myocardial Injury in Myocarditis |
|  | Proteins Involved in Myocarditis |
|  | SOCS in Negative Feedback Regulation in Myocarditis |
|  | Hair Follicle Keratinocyte Apoptosis |
|  | Proteins Involved in Atopic Dermatitis |
|  | Melanoma |
|  | Apoptosis Block in Melanoma Cell |
|  | Proteins Involved in Melanoma |
|  | Proteins Involved in Psoriasis |
|  | T-Cells Differentiation Block in Psoriasis |
|  | IFN-gamma/TNF-alpha Mediated Cell Proliferation in Psoriasis |
|  | IL22 Induces Keratinocyte Proliferation in Psoriasis |
|  | Proteins with Altered Expression in Psoriasis |
|  | Th17-Cell Function in Systemic Lupus Erythematosus |
|  | Vitiligo |
|  | T-Cell Cytotoxic Response against Melanocytes in Vitiligo |
|  | Toll-like Receptors in beta-Cell |
|  | Donohue Syndrome Progression (Hypothesis) |
|  | Tubular Cell Dysfunction in Progressive Diabetic Nephropathy |
|  | Endothelial Cell Dysfunction in Progressive Diabetic Nephropathy |
|  | Podocyte Dysfunction in Diabetic Nephropathy |
|  | Proteins Involved in Diabetic Nephropathy |
|  | Proliferative Diabetic Retinopathy |
|  | Adipokines and Cytokines in Insulin Resistance in Skeletal Muscles |
|  | Proteins Involved in Insulin Resistance |
|  | Insulin Resistance in Myocytes Induced by Obesity |
|  | Adiponectin Synthesis Declines in Insulin Resistance |
|  | Insulin Resistance in Hepatocytes |
|  | Hepatic Stellate Cells in non-Alcoholic Fatty Liver Disease |
|  | Proteins Involved in non-Alcoholic Fatty Liver Disease |
|  | Oxidation of Fatty Acid in non-Alcoholic Fatty Liver Disease |
|  | Leptin in Insulin Synthesis and Secretion |
|  | FOXO1 and SREBP-1C Role in beta-Cell Suppression (Rodent Model) |
|  | Hashimoto's Thyroiditis |
|  | RET1 Mutation in Hyperparathyroidism |
|  | Thyroid Hormones in Adipose Tissue Metabolism |
|  | Thyroid Dysfunction |
|  | Graves Ophthalmopathy |
|  | KISS1 Production Impairment in Hypogonadotropic hypogonadism |
|  | Thyroid Stimulating Hormone (TSH) Resistance in Congenital Hypothyroidism |
|  | Overt Hypothyroidism, Primary |
|  | Thyrotropin Releasing Hormone (TRH) Hypothalamic Release in non-Autoimmune Hypothyroidism |
|  | Central Hypothyroidism |
|  | Tertiary Hypothyroidism |
|  | SOCS3 and PTPN1 in Hypothalamic Neuron Insensitivity to Insulin and Leptin |
|  | Adipokines Production by Adipocyte Impaired in Obesity |
|  | Proteins Involved in Obesity |
|  | CD4+T-Cell Response in Celiac Disease |
|  | Proteins Involved in Colorectal Neoplasms |
|  | Cetuximab Resistance in Colorectal Cancer |
|  | Metastatic Colorectal Cancer |
|  | EGFR Nuclear Signaling in Colorectal Cancer |
|  | Crohn's Disease |
|  | Paneth-Cell Function in Crohn's Disease |
|  | Th17-Cell Activation in Crohn's Disease |
|  | Proteins Involved in Inflammatory Bowel Diseases |
|  | Peutz-Jeghers Syndrome |
|  | Nicotine anti-Inflammatory Effect in Ulcerative Colitis |
|  | Proteins Involved in Ulcerative Colitis |
|  | Apoptosis Block in Acute Myeloid Leukemia Cell |
|  | RARA Signaling in Acute Myeloid Leukemia (M3) |
|  | Acute Myeloid Leukemia |
|  | B-Cell Acute Lymphoblastic Leukemia |
|  | Chronic Myeloid Leukemia |
|  | Diffuse Large-B-Cell Lymphoma |
|  | Diffuse Large-B-Cell Lymphoma ABC Subtype |
|  | Proteins Involved in Diffuse Large-B-Cell Lymphoma |
|  | Follicular Lymphoma |
|  | Proteins Involved in Hodgkin Lymphoma |
|  | Proteins Involved in Mantle Cell Lymphoma |
|  | Mantle Cell Lymphoma |
|  | IL6/IGF1/VEGFA Signaling in Multiple Myeloma |
|  | Multiple Myeloma |
|  | Proteins Involved in Multiple Myeloma |
|  | T-Cell Acute Lymphoblastic Leukemia |
|  | Proteins Involved in Hepatocellular Carcinoma |
|  | Hepatocellular Carcinoma |
|  | Growth Factor Signaling in Hepatocellular Carcinoma |
|  | Proteins Involved in Pancreatic Cancer |
|  | Pancreatic Ductal Carcinoma |
|  | Growth Factor Signaling in Pancreatic Neoplasms |
|  | TRPM7/8 in Pancreatic Adenocarcinoma |
|  | Eosinophil Survival in Asthma |
|  | Il17 Signaling Related Neutrophilia in Asthma |
|  | TSLP Signaling in Bronchial Epithelial Cell in Asthma |
|  | Airway Smooth Muscle Cell High Rate Proliferation |
|  | IgE Induces Airway Smooth Muscle Cell Proliferation |
|  | Goblet-Cell Related Mucus Secretion in Asthma |
|  | Basophil Activation in Asthma |
|  | Mast-Cell Activation in Asthma |
|  | Th17-Cell Differentiation in Asthma |
|  | Mucin Production in Goblet Airway Epithelial Cells |
|  | Osteoclast Activation in Gout |
|  | Neutrophil Recruitment in Sinovium in Gout |
|  | Dendritic Cell Dysfunction in Psoriatic Arthritis |
|  | Th17-Cell and Th1 Immune Responsein Psoriatic Arthritis |
|  | Osteoclast Activation in Psoriatic Arthritis |
|  | Keratinocyte Activation in Psoriatic Arthritis |
|  | Proteins Involved in Psoriatic Arthritis |
|  | Synovial Fibroblast Proliferation in Rheumatoid Arthritis |
|  | Osteoclast Activation in Rheumatoid Arthritis |
|  | Autocrine Cytokine/Chemokine Loops in Systemic Scleroderma |
|  | Skin Fibrosis |
|  | Th17-Cell Numbers Reduction in HIV |
|  | Diabetes Induced Periodontitis |
|  | Cowden Syndrome |
|  | Gluten Impact on Neuronal System (Hypothesis) |
|  | beta-Casomorphins/Opioid Receptors Affect Brain Function (Hypothesis) |
|  | IL6 and Insulin Resistance in Muscular Dystrophies |
|  | IL6 Promotes Inflammation in Muscular Dystrophies |
|  | Bone Loss in Osteoporosis |
|  | Proteins with Altered Expression in Rheumatoid Arthritis |
|  | Proteins Involved in Epilepsy |
|  | BDNF Upregulation Triggered by Seizures |
|  | Glioblastoma, Primary |
|  | Proteins Involved in Glioblastoma |
|  | Glioblastoma, Secondary |
|  | Astrocytoma |
|  | Proteins Involved in Astrocytoma |
|  | Proteins Involved in Glioma |
|  | Glioma Stem Cell Program Activation |
|  | Neuroblastoma |
|  | ALK Associated Neuroblastoma |
|  | Proteins Involved in Parkinson's Disease |
|  | IL6/IL12 Signaling Activates Immune System in Multiple Sclerosis |
|  | Proteins Involved in Breast Cancer Related to ESR1 Signaling Pathway |
|  | Proteins Involved in Breast Cancer Related to ERBB2/VEGFR/Akt Signaling Pathway |
|  | ERBB/VEGFR/Akt Signaling in Breast Cancer |
|  | IGF1R/AKT Signaling in Breast Cancer |
|  | ESR1/ERBB Positive Luminal Breast Cancer |
|  | Proteins Involved in Breast Cancer Related to IGF1R/Akt Signaling Pathway |
|  | Breast Cancer |
|  | ESR1 Signaling in Breast Cancer |
|  | Endometrial Cancer |
|  | Endometrioid Endometrial Cancer |
|  | Clear Cell Endometrial Cancer and Papillary Serous Endometrial Cancer |
|  | Proteins Involved in Endometriosis |
|  | Leptin Role in Endometriosis |
|  | Proteins Involved in High-Grade Serous Ovarian Carcinoma |
|  | Endometrioid Ovarian Carcinoma |
|  | Proteins Involved in Endometrioid Ovarian Carcinoma |
|  | Clear Cell Ovarian Carcinoma |
|  | Proteins Involved in Mucinous Ovarian Carcinoma |
|  | High-Grade Serous Ovarian Carcinoma |
|  | Low-Grade Serous Ovarian Carcinoma |
|  | Proteins Involved in Low-Grade Serous Ovarian Carcinoma |
|  | Ovarian Cancer |
|  | Proteins Involved in Clear Cell Ovarian Carcinoma |
|  | Mucinous Ovarian Carcinoma |
|  | MIF Role in Cholesteatoma |
|  | IL6/JAK/STAT3 Signaling in Cholesteatoma |
|  | Cholesteatoma |
|  | Prostate Cancer |
|  | Proteins Involved in Prostate Cancer |
|  | TLR3 Role in Retinal Pigment Epithelial Cell |
|  | IL10/STAT3 Signaling in M2 Macrophage and Retinal Angiogenesis |
|  | Neurotrophic Factor Deprivation in Retinal Ganglion Cell Death |
|  | Proteins Involved in Noise-Induced Hearing Loss |
|  | Cochlear Inflammation (Noise-Induced) |
|  | Alveolar Macrophage Negative Regulation Declined in COPD |
|  | Mucin Hyperproduction in Goblet and Mucous Cells |
|  | Polycystins Mutation Effects in Cystic Kidney Disease |
|  | Proteins Involved in Polycystic Kidney Disease |
|  | Proteins Involved in Glomerulonephritis |
|  | Endothelial Cell Dysfunction in Glomerulonephritis |
|  | Endothelial Cell Dysfunction in Pyelonephritis |
|  | ANGPT2 -> STAT Expression Targets |
|  | ANGPT1 -> STAT Expression Targets |
|  | AREG -> STAT Expression Targets |
|  | Noradrenaline/Gs Expression Targets |
|  | Noreadrenaline/Gq Expression Targets |
|  | Epinephrine/Gs Expression Targets |
|  | Serotonin/Gq Expression Targets |
|  | Epinephrine/Gq Expression Targets |
|  | BTC -> STAT Expression Targets |
|  | IFNG -> ARRB1/STAT1 Signaling |
|  | CD2 Expression Targets |
|  | CD22 -> STAT Expression Targets |
|  | CD8 -> NFATC Expression Targets |
|  | CD80 -> ATF/CREB/CREBBP Expression Targets |
|  | CD86 -> ATF/CREB/CREBBP Expression Targets |
|  | CLCF1 Expression Targets |
|  | CXCL12 Expression Targets |
|  | CCL3 Expression Targets |
|  | CCL3L1 Expression Target |
|  | CCL16 Expression Targets |
|  | CCL5 Expression Targets |
|  | CCL7 Expression Target |
|  | CCL7 Expression Targets |
|  | CCL8 Expression Targets |
|  | CCL15 Expression Targets |
|  | CCL2 Expression Targets |
|  | IL8 Expression Targets |
|  | CSF3 Expression Targets |
|  | CSF2 -> STAT Expression Targets |
|  | CNTF Expression Targets |
|  | Clozapine Induced Granulocytopenia |
|  | EGF -> STAT Expression Targets |
|  | EGFR/ERBB -> STAT Signaling |
|  | EREG -> STAT Expression Targets |
|  | Erythropoietin -> STAT Expression Targets |
|  | PECAM1 Expression Targets |
|  | FGF2 -> STAT Expression Targets |
|  | FGF10 -> STAT Expression Targets |
|  | FGF18 -> STAT Expression Targets |
|  | FGF1 -> STAT Expression Targets |
|  | FGF9 -> STAT Expression Targets |
|  | FGF8 -> STAT Expression Targets |
|  | FGFR1 -> STAT Signaling |
|  | FGFR3 -> STAT Signaling |
|  | GH2/GHR Expression Target |
|  | GH1/GHR -> STAT Expression Targets |
|  | CSH1/GHR Expression Targets |
|  | CSH1/PRLR Expression Targets |
|  | GH1/PRLR Expression Targets |
|  | HBEGF -> STAT Expression Targets |
|  | HGF -> STAT Expression Targets |
|  | HGFR -> STAT Signaling |
|  | TAM Receptor Signaling Activation in Cancer |
|  | Receptors and Adaptor Proteins Activated in Cancer |
|  | Proteins with Altered Expression in Cancer-Associated Sustaining of Proliferative Signaling |
|  | Genes with Mutations in Cancer-Associated Sustaining of Proliferative Signaling |
|  | STAT3 and NFkB Activate Inflammation-Induced Tumorigenesis |
|  | IL23A and IL17A Provoke Cancer-Associated Inflammation |
|  | STAT3 Inhibits Maturation of Dendritic Cells and Facilitates Cancer Progression |
|  | STAT3 Facilitates the Function of Treg Cells and Cancer Progression |
|  | Proteins with Altered Expression in Tumor-Promoting Inflammation |
|  | N2 Neutrophils in Tumor-Promoting Inflammation and Tumor Progression |
|  | Apoptosis Block by Inhibitor of Apoptosis Proteins (IAPs) in Cancer |
|  | Proteins with Altered Expression in Cancer-Associated Resisting to Cell Death |
|  | Suppression of Caspase Activity in Cancer |
|  | Apoptosis Evasion in Cancer: Overview |
|  | TERT Activation in Cancer |
|  | Proteins with Altered Expression in Cancer-Associated Enabling of Replicative Immortality |
|  | VEGFA Dependent Angiogenesis in Cancer |
|  | VEGF Independent Angiogenesis in Cancer |
|  | Proteins with Altered Expression in Cancer Metastases |
|  | Epithelial to Mesenchymal Transition in Cancer: Overview |
|  | BSG (CD147) in Cancer Cells Motility, Invasion and Survival |
|  | Hyaluronic Acid, CD44 and HMMR in Cancer Cell Invasion and Survival |
|  | Skeletal Muscle Wasting in Cancer Cachexia |
|  | Myeloid Derived Suppressor Cells in Cancer Immune Escape |
|  | IDO1 in Cancer Immune Escape |
|  | CD46/CD55/CD59 Inhibit Complement Mediated Lysis of Cancer Cells |
|  | EZH2 Methyltransferase Role in Cancer |
|  | IGF2 -> STAT Expression Targets |
|  | IGF1 -> STAT Expression Targets |
|  | Insulin -> STAT Expression Targets |
|  | CD2 -> STAT Signaling |
|  | ICAM2 -> CTNNB/FOXO/STAT3 Signaling |
|  | PECAM -> STAT Signaling |
|  | InsulinR -> STAT Signaling |
|  | IGF1R -> STAT Signaling |
|  | Elevated Receptors -> Expression Targets in Skin |
|  | IFNLR1-> STAT/NFKB Signaling |
|  | IFNW1/IFNR Expression Target |
|  | IFNA1/IFNR Expression Targets |
|  | IFNB1/IFNR Expression Targets |
|  | IFNG/IFNR Expression Targets |
|  | IL22R -> STAT3 Signaling |
|  | IL31R -> STAT Signaling |
|  | IL15R -> STAT Signaling |
|  | IL1R -> STAT3 Signaling |
|  | IL10R -> STAT Signaling |
|  | IL13R -> STAT Signaling |
|  | IL3R -> STAT Signaling |
|  | IL27R -> STAT Signaling |
|  | IL7R -> STAT Signaling |
|  | IL9R -> STAT Signaling |
|  | IL5R -> STAT Signaling |
|  | IL11R -> STAT3 Signaling |
|  | IL6R -> STAT Signaling |
|  | IL2R -> STAT Signaling |
|  | IL12R -> STAT Signaling |
|  | IL23R -> STAT3/NF-kB Signaling |
|  | IL21R -> STAT Signaling |
|  | IL7 Expression Targets |
|  | IL13 Expression Targets |
|  | IL21 Expression Targets |
|  | IL15 Expression Targets |
|  | IL12B Expression Targets |
|  | IL3 Expression Targets |
|  | IL16 -> STAT Expression Targets |
|  | IL6 Expression Targets |
|  | IL10 Expression Targets |
|  | IL5 Expression Targets |
|  | IL22 Expression Targets |
|  | IL11 Expression Targets |
|  | IL9 Expression Targets |
|  | IL31 Expression Targets |
|  | IL2 Expression Targets |
|  | Leptin -> STAT Expression Targets |
|  | Morphine Expression Targets |
|  | NRG1 -> STAT Expression Targets |
|  | OSM/OSMR Expression Targets |
|  | PDGFC -> STAT Expression Target |
|  | PDGFD -> STAT Expression Targets |
|  | PDGF -> STAT Expression Targets |
|  | PDGFB -> STAT Expression Target |
|  | PDGFR -> STAT Signaling |
|  | CSF2 -> STAT Signaling |
|  | KIT -> STAT Signaling |
|  | GAST Expression Targets in GAST Expression Targets |
|  | POMC Expression Targets |
|  | CCL11 Expression Targets |
|  | AVP/Gq -> STAT Expression Targets |
|  | IFNA1/Gq Expression Targets |
|  | AVP/Gs -> STAT Expression Targets |
|  | AGT -> STAT Expression Targets |
|  | PRL/PRLR Expression Targets |
|  | PRL/GHR -> STAT Expression Targets |
|  | ProlactinR -> STAT Signaling |
|  | PDCD1 -> STAT Expression Targets |
|  | ALK -> STAT Signaling |
|  | AngiopoietinR -> STAT Signaling |
|  | EphrinR -> STAT Signaling |
|  | CSF1 -> STAT Expression Targets |
|  | MDK/PTN Expression Targets |
|  | KITLG -> STAT Expression Targets |
|  | EphrinR Expression Targets |
|  | EFNA1 -> STAT Expression Target |
|  | PAF Expression Targets |
|  | FOXO1 Signaling |
|  | BMPR2 Signaling |
|  | Ras-GAP Regulation Signaling |
|  | Estrogens/ESR1 non-Genomic Signaling |
|  | EGFR Signaling |
|  | TCR -> STAT Expression Targets |
|  | T-Cell Receptor -> STAT Signaling |
|  | TGFA -> STAT Expression Targets |
|  | LTA Expression Targets |
|  | CD40LG -> STAT Expression Targets |
|  | TNF -> STAT Expression Targets |
|  | TNFRSF5 -> STAT Signaling |
|  | TNFRSF1A -> STAT Signaling |
|  | Thrombopoietin -> STAT Expression Targets |
|  | IL1B -> PGE2 Expression Targets |
|  | IL1B Expression Targets |
|  | IL1A Expression Targets |
|  | VEGFA -> STAT Expression Targets |
|  | VEGFR -> STAT Signaling |
|  | PPAR Psoriasis |
|  | pprarg neg, uknown targets, ps-positive |
|  | prarg neg,ukn expres targets, ps-positive |
|  | PPARG negative targets |
|  | pparg expr target\_regulators |
|  | Neighbors of disease exacerbation |
|  | Neighbors of tumor progression |
|  | Neighbors of cancer progression |
|  | Neighbors of inflammatory status |
|  | Neighbors of overall survival |
|  | Neighbors of disease severity |
|  | Neighbors of tumor size |
|  | Neighbors of lesion size |
|  | Neighbors of mortality |
|  | Neighbors of survival rate |
|  | Neighbors of infarct size |
|  | Neighbors of survival time |
|  | Neighbors of functional recovery |
|  | Neighbors of insulin sensitivity |
|  | Neighbors of bacterial load |
|  | Neighbors of cancer survival |
|  | Neighbors of graft survival |
|  | Neighbors of therapeutic efficacy |
|  | Neighbors of clinical stage |
|  | Neighbors of lethality |
|  | Neighbors of tumor regression |
|  | Neighbors of recurrence-free survival |
|  | Neighbors of disease-free survival |
|  | Neighbors of tumor response |
|  | Neighbors of macrophage count |
|  | Neighbors of long-term survival |
|  | Neighbors of treatment outcome |
|  | Neighbors of body weight |
|  | Neighbors of body weight gain |
|  | Neighbors of cardiovascular risk |
|  | Neighbors of microvessel density |
|  | Neighbors of tumor recurrence |
|  | Neighbors of tumor promotion |
|  | Neighbors of neutrophil count |
|  | Neighbors of glucose tolerance |
|  | Neighbors of morbidity |
|  | Neighbors of fibrosis progression |
|  | Neighbors of mortality rate |
|  | Neighbors of fat mass |
|  | Neighbors of exhaustion |
|  | Neighbors of adverse outcome |
|  | Neighbors of radioresistance |
|  | Neighbors of lesion area |
|  | Neighbors of cell density |
|  | Neighbors of cancer-specific survival |
|  | Neighbors of progression-free survival |
|  | Neighbors of apoptosis rate |
|  | Neighbors of early mortality |
|  | Neighbors of viral load |
|  | Neighbors of blood pressure |
|  | Neighbors of tumor grade |
|  | Neighbors of body mass index |
|  | Neighbors of vascular density |
|  | Neighbors of muscle mass |
|  | Neighbors of cancer risk |
|  | Neighbors of cancer incidence |
|  | Neighbors of tumor aggressiveness |
|  | Neighbors of systolic blood pressure |
|  | Neighbors of tumor resistance |
|  | Neighbors of blood glucose |
|  | Neighbors of ejection fraction |
|  | Neighbors of median survival |
|  | Neighbors of fasting plasma glucose |
|  | Neighbors of T-cell count |
|  | Neighbors of antimetastatic activity |
|  | Neighbors of energy expenditure |
|  | Neighbors of glucose level |
|  | Neighbors of vascular tone |
|  | Neighbors of radiosensitivity |
|  | Neighbors of breast cancer risk |
|  | Neighbors of proliferation index |
|  | Neighbors of cell permeability |
|  | Neighbors of infarct area |
|  | Neighbors of fertility |
|  | Neighbors of pathological response |
|  | Neighbors of lymphocyte count |
|  | Neighbors of skin thickness |
|  | Neighbors of metabolic response |
|  | Neighbors of insulin level |
|  | Neighbors of bone volume |
|  | Neighbors of fractional shortening |
|  | Neighbors of disease exacerbation |
|  | Neighbors of tumor progression |
|  | Neighbors of cancer progression |
|  | Neighbors of inflammatory status |
|  | Neighbors of overall survival |
|  | Neighbors of disease severity |
|  | Neighbors of tumor size |
|  | Neighbors of lesion size |
|  | Neighbors of mortality |
|  | Neighbors of survival rate |
|  | Neighbors of infarct size |
|  | Neighbors of survival time |
|  | Neighbors of functional recovery |
|  | Neighbors of insulin sensitivity |
|  | Neighbors of bacterial load |
|  | Neighbors of cancer survival |
|  | Neighbors of graft survival |
|  | Neighbors of therapeutic efficacy |
|  | Neighbors of clinical stage |
|  | Neighbors of lethality |
|  | Neighbors of tumor regression |
|  | Neighbors of recurrence-free survival |
|  | Neighbors of disease-free survival |
|  | Neighbors of tumor response |
|  | Neighbors of macrophage count |
|  | Neighbors of long-term survival |
|  | Neighbors of treatment outcome |
|  | Neighbors of body weight |
|  | Neighbors of body weight gain |
|  | Neighbors of cardiovascular risk |
|  | Neighbors of microvessel density |
|  | Neighbors of tumor recurrence |
|  | Neighbors of tumor promotion |
|  | Neighbors of neutrophil count |
|  | Neighbors of glucose tolerance |
|  | Neighbors of morbidity |
|  | Neighbors of fibrosis progression |
|  | Neighbors of mortality rate |
|  | Neighbors of fat mass |
|  | Neighbors of exhaustion |
|  | Neighbors of adverse outcome |
|  | Neighbors of radioresistance |
|  | Neighbors of lesion area |
|  | Neighbors of cell density |
|  | Neighbors of cancer-specific survival |
|  | Neighbors of progression-free survival |
|  | Neighbors of apoptosis rate |
|  | Neighbors of early mortality |
|  | Neighbors of viral load |
|  | Neighbors of blood pressure |
|  | Neighbors of tumor grade |
|  | Neighbors of body mass index |
|  | Neighbors of vascular density |
|  | Neighbors of muscle mass |
|  | Neighbors of cancer risk |
|  | Neighbors of cancer incidence |
|  | Neighbors of tumor aggressiveness |
|  | Neighbors of systolic blood pressure |
|  | Neighbors of tumor resistance |
|  | Neighbors of blood glucose |
|  | Neighbors of ejection fraction |
|  | Neighbors of median survival |
|  | Neighbors of fasting plasma glucose |
|  | Neighbors of T-cell count |
|  | Neighbors of antimetastatic activity |
|  | Neighbors of energy expenditure |
|  | Neighbors of glucose level |
|  | Neighbors of vascular tone |
|  | Neighbors of radiosensitivity |
|  | Neighbors of breast cancer risk |
|  | Neighbors of proliferation index |
|  | Neighbors of cell permeability |
|  | Neighbors of infarct area |
|  | Neighbors of fertility |
|  | Neighbors of pathological response |
|  | Neighbors of lymphocyte count |
|  | Neighbors of skin thickness |
|  | Neighbors of metabolic response |
|  | Neighbors of insulin level |
|  | Neighbors of bone volume |
|  | Neighbors of fractional shortening |
|  | Neighbors of cell development |
|  | Neighbors of immune response |
|  | Neighbors of cell population |
|  | Neighbors of T-cell development |
|  | Neighbors of inflammatory response |
|  | Neighbors of wound healing |
|  | Neighbors of angiogenesis |
|  | Neighbors of innate immune response |
|  | Neighbors of cell function |
|  | Neighbors of macrophage activation |
|  | Neighbors of cell formation |
|  | Neighbors of adaptive immune response |
|  | Neighbors of osteoclast development |
|  | Neighbors of epithelial to mesenchymal transition |
|  | Neighbors of immunity |
|  | Neighbors of cell proliferative response |
|  | Neighbors of chemotaxis |
|  | Neighbors of dendritic cell differentiation |
|  | Neighbors of cell survival |
|  | Neighbors of osteoclast differentiation |
|  | Neighbors of cell phenotype |
|  | Neighbors of T-cell proliferation |
|  | Neighbors of tissue remodeling |
|  | Neighbors of cell adhesion |
|  | Neighbors of T-cell activation |
|  | Neighbors of endothelial cell proliferation |
|  | Neighbors of fibrogenesis |
|  | Neighbors of tumor growth |
|  | Neighbors of dendritic cell development |
|  | Neighbors of tissue repair |
|  | Neighbors of cell count |
|  | Neighbors of cell infiltration |
|  | Neighbors of cell interaction |
|  | Neighbors of SMC proliferation |
|  | Neighbors of lumen formation |
|  | Neighbors of endothelial cell migration |
|  | Neighbors of adipogenesis |
|  | Neighbors of lipid storage |
|  | Neighbors of cell damage |
|  | Neighbors of pregnancy |
|  | Neighbors of monocyte differentiation |
|  | Neighbors of aging |
|  | Neighbors of cell motility |
|  | Neighbors of monocyte migration |
|  | Neighbors of fibroblast proliferation |
|  | Neighbors of adipocyte differentiation |
|  | Neighbors of macrophage migration |
|  | Neighbors of microglial activation |
|  | Neighbors of neutrophil recruitment |
|  | Neighbors of regeneration |
|  | Neighbors of cell migration |
|  | Neighbors of cancer growth |
|  | Neighbors of stem cell proliferation |
|  | Neighbors of smooth muscle cell migration |
|  | Neighbors of hepatic regeneration |
|  | Neighbors of transendothelial migration |
|  | Neighbors of phagocytosis |
|  | Neighbors of immune system activation |
|  | Neighbors of leukocyte recruitment |
|  | Neighbors of osteoclast formation |
|  | Neighbors of cell invasion |
|  | Neighbors of heart function |
|  | Neighbors of hemopoiesis |
|  | Neighbors of bone remodeling |
|  | Neighbors of ossification |
|  | Neighbors of T-cell function |
|  | Neighbors of immune system function |
|  | Neighbors of lung development |
|  | Neighbors of autophagy |
|  | Neighbors of monocyte adhesion |
|  | Neighbors of transcription activation |
|  | Neighbors of senescence |
|  | Neighbors of T-cell response |
|  | Neighbors of tumor immunity |
|  | Neighbors of hepatocyte proliferation |
|  | Neighbors of cell transdifferentiation |
|  | Neighbors of neuroprotection |
|  | Neighbors of stem cell differentiation |
|  | Neighbors of endothelial cell function |
|  | Neighbors of neuronal death |
|  | Neighbors of cell growth |
|  | Neighbors of ROS generation |
|  | Neighbors of epithelial cell proliferation |
|  | Neighbors of first trimester pregnancy |
|  | Neighbors of cancer cell growth |
|  | Neighbors of sensitization |
|  | Neighbors of hemato-encephalic barrier |
|  | Neighbors of macrophage function |
|  | Neighbors of cellular immune response |
|  | Neighbors of cellular senescence |
|  | Neighbors of colony formation |
|  | Neighbors of macrophage differentiation |
|  | Neighbors of cell differentiation |
|  | Neighbors of keratinocyte proliferation |
|  | Neighbors of stem cell migration |
|  | Neighbors of life span |
|  | Neighbors of cell homeostasis |
|  | Neighbors of psoriasis |
|  | Neighbors of inflammatory disease |
|  | Neighbors of rheumatoid arthritis |
|  | Neighbors of atherosclerosis |
|  | Neighbors of fibrosis |
|  | Neighbors of colitis |
|  | Neighbors of arthritis |
|  | Neighbors of inflammatory bowel disease |
|  | Neighbors of injury |
|  | Neighbors of atherogenesis |
|  | Neighbors of vascular remodeling |
|  | Neighbors of metastasis |
|  | Neighbors of carcinogenesis |
|  | Neighbors of multiple sclerosis |
|  | Neighbors of chronic inflammation |
|  | Neighbors of inflammation |
|  | Neighbors of tumor microenvironment |
|  | Neighbors of pathological angiogenesis |
|  | Neighbors of obesity |
|  | Neighbors of vascular disease |
|  | Neighbors of diabetes mellitus |
|  | Neighbors of asthma |
|  | Neighbors of pneumonia |
|  | Neighbors of hyperplasia |
|  | Neighbors of osteoarthritis |
|  | Neighbors of liver fibrosis |
|  | Neighbors of chronic obstructive pulmonary disease |
|  | Neighbors of myocardial infarction |
|  | Neighbors of leukocyte infiltration |
|  | Neighbors of reperfusion injury |
|  | Neighbors of airway inflammation |
|  | Neighbors of hepatitis |
|  | Neighbors of insulin resistance |
|  | Neighbors of infection |
|  | Neighbors of vascular injury |
|  | Neighbors of renal injury |
|  | Neighbors of preeclampsia |
|  | Neighbors of macrophage infiltration |
|  | Neighbors of liver injury |
|  | Neighbors of neuroinflammation |
|  | Neighbors of sepsis |
|  | Neighbors of death |
|  | Neighbors of type 1 diabetes |
|  | Neighbors of encephalomyelitis |
|  | Neighbors of autoimmune disease |
|  | Neighbors of ischemic stroke |
|  | Neighbors of dermatitis |
|  | Neighbors of neutrophil infiltration |
|  | Neighbors of heart disease |
|  | Neighbors of diabetic nephropathy |
|  | Neighbors of lung injury |
|  | Neighbors of immunopathology |
|  | Neighbors of systemic sclerosis |
|  | Neighbors of hepatocellular carcinoma |
|  | Neighbors of autoimmunity |
|  | Neighbors of acute lung injury |
|  | Neighbors of gastric cancer |
|  | Neighbors of experimental autoimmune encephalomyelitis |
|  | Neighbors of colorectal cancer |
|  | Neighbors of cardiac remodeling |
|  | Neighbors of breast cancer |
|  | Neighbors of ischemia |
|  | Neighbors of systemic lupus erythematosus |
|  | Neighbors of melanoma |
|  | Neighbors of collagen-induced arthritis |
|  | Neighbors of interstitial fibrosis |
|  | Neighbors of neoplasm invasion |
|  | Neighbors of kidney disease |
|  | Neighbors of heart failure |
|  | Neighbors of lung disease |
|  | Neighbors of pulmonary fibrosis |
|  | Neighbors of cardiovascular disease |
|  | Neighbors of stroke |
|  | Neighbors of cancer |
|  | Neighbors of cerebral ischemia |
|  | Neighbors of type 2 diabetes |
|  | Neighbors of neoplasm |
|  | Neighbors of endometriosis |
|  | Neighbors of neointima |
|  | Neighbors of vasculitis |
|  | Neighbors of atrophy |
|  | Neighbors of diet-induced obesity |
|  | Neighbors of respiratory hypersensitivity |
|  | Neighbors of myocardial fibrosis |
|  | Neighbors of synovitis |
|  | Neighbors of coronary artery disease |
|  | Neighbors of rupture |
|  | Neighbors of endothelial cell dysfunction |
|  | Neighbors of liver disease |
|  | Neighbors of prostate cancer |
|  | Neighbors of hypertrophy |
|  | Neighbors of osteolysis |
|  | Neighbors of ovarian cancer |
|  | Neighbors of pancreatic cancer |
|  | Neighbors of hypertension |
|  | Neighbors of intimal hyperplasia |
|  | Neighbors of proteinuria |
|  | Neighbors of epithelial cell |
|  | Neighbors of keratinocyte |
|  | Neighbors of monocyte |
|  | Neighbors of macrophage |
|  | Neighbors of intestine epithelium cell |
|  | Neighbors of hepatocyte |
|  | Neighbors of endothelial cell |
|  | Neighbors of dendritic cell |
|  | Neighbors of inflammatory cell |
|  | Neighbors of neutrophil |
|  | Neighbors of astrocyte |
|  | Neighbors of cardiac myocyte |
|  | Neighbors of myeloid cell |
|  | Neighbors of stem cell |
|  | Neighbors of leukocyte |
|  | Neighbors of insulin-secreting cell |
|  | Neighbors of T-cell |
|  | Neighbors of hematopoietic cell |
|  | Neighbors of podocyte |
|  | Neighbors of antigen-presenting cell |
|  | Neighbors of lung |
|  | Neighbors of liver |
|  | Neighbors of kidney |
|  | Neighbors of skin |
|  | Neighbors of intestine |
|  | Neighbors of lymph node |
|  | Neighbors of colon |
|  | Neighbors of blood vessel |
|  | Neighbors of heart |
|  | Neighbors of microvessel |
|  | Neighbors of uterus |
|  | Neighbors of esophagus |
|  | Neighbors of retina |
|  | Neighbors of brain |
|  | Neighbors of ovary |
|  | Neighbors of breast |
|  | Neighbors of blood vessel wall |
|  | Neighbors of stomach |
|  | Neighbors of artery |
|  | Neighbors of pancreas |
|  | Neighbors of prostate |
|  | Neighbors of decidua |
|  | Neighbors of immune system |
|  | Neighbors of vein |
|  | Neighbors of ankle joint |
|  | Neighbors of thymus gland |
|  | Neighbors of brain blood vessel |
|  | Neighbors of myelin sheath |
|  | Neighbors of blastocyst |
|  | Neighbors of nerve |
|  | Neighbors of NF-kB family |
|  | Neighbors of PPAR |
|  | Neighbors of PI3K |
|  | Neighbors of STAT family |
|  | Neighbors of mitogen-activated protein kinase |
|  | Neighbors of IFNAR ligand |
|  | Neighbors of cytokine |
|  | Neighbors of JAK |
|  | Neighbors of Ras GTPase |
|  | Neighbors of ERK1/2 |
|  | Neighbors of ovalbumin |
|  | Neighbors of IgG |
|  | Neighbors of SMAD subfamily |
|  | Neighbors of estrogen receptor |
|  | Neighbors of Notch |
|  | Neighbors of heat shock protein 90 |
|  | Neighbors of proteasome endopeptidase complex |
|  | Neighbors of non-coding RNA |
|  | Neighbors of STAT5 |
|  | Neighbors of retinoid-X receptor subfamily |
|  | Neighbors of WNT |
|  | Neighbors of PP2B |
|  | Neighbors of TORC1 |
|  | Neighbors of Rho kinase |
|  | Neighbors of PDGF receptor |
|  | Neighbors of caspase |
|  | comon ps\_pos, pprarg\_neg targets |
|  | Model of PPARG signaling in psoriasis |
|  | PPARG negative regulators and targets |
|  | Model of PPARG related pathways in psoriasis (short version) |
|  | New Pathway (5) |
|  | New Pathway (3) |
|  | sars-cov no binding |
|  | H2A-K119 and H3-K27 |
|  | New Pathway |
|  | IFN signaling in alveolar cells |
|  | Possible Interplay between SOX11, CCND1 and EZH2 in Mantle Cell Lymphoma |
|  | Figure 3\_Mixed network of dexamethasone targets |
|  | Figure 3\_Mixed network of dexamethasone targets |
|  | 1\_Differentiation of psoriatic T cells |
|  | 2\_Interleukin-17 and interleukin-22 signaling in psoriasis |
|  | 1\_Formation of cysts in polycystic kidney disease |
|  | 2\_The role of endothelial cells in pyelonephritis |
|  | 3\_The role of interstitial fibroblasts in pyelonephritis |
|  | 3\_Mesangial cell dysfunction in glomerulonephritis |
|  | 3\_Role of neurotrophic factor deprivation in retinal ganglion cell death |
|  | 1\_Endothelial cells stimulate vasoconstriction and thrombosis in pulmonary hypertension |
|  | 3\_Increased proliferation of pulmonary artery smooth muscle cells (PASMCs) in familial forms of pulmonary hypertension |
|  | 2\_Cardiomyocyte hypertrophy |
|  | 1\_Th2 cell response in asthma |
|  | 3\_1\_Mucus accumulation in asthma |
|  | 2\_2\_Eosinophilia and neutrophilia in asthma: eosinophil apoptosis |
|  | 4\_Mucin hyperproduction in goblet and mucous cells in COPD |
|  | 2\_Paneth cell dysfunction in Crohn's disease |
|  | 1\_Defects in response to pathogens in the gut promotes inflammation in Crohn's disease |
|  | 4\_1\_Polymorphisms associated with inflammatory bowel diseases |
|  | 1\_Inflammatory mediators increase the production of hepcidin (HAMP) |
|  | 4\_Inflammatory mediators inhibit GATA signaling in erythropoiesis |
|  | 4\_2\_Insulin resistance: inflammation-related insulin resistance |
|  | 4\_3\_Insulin resistance: FFA-related insulin resistance |
|  | 1\_Antigen-presenting cells promote islet dysfunction and immune system activation in T1D |
|  | 2\_Defective tolerance of autoreactive T-cell in T1D |
|  | 1\_Decreased secretion of thyroid hormones |
|  | Model of PPARG signaling in psoriais (tested) |
|  | before laser treatment |
|  | New Pathway |
|  | Differentiation of psoriatic T cells |
|  | Interleukin-17 and interleukin-22 signaling in psoriasis |
|  | Anti-psoriatic drugs influence PPARG signaling |
|  | PPARG signaling after laser treatment |

---

|  |  |
| --- | --- |
| Group | Genes with Mutations Associated with Inflammatory Bowel Diseases |
|  | Proteins Involved in Cowden Syndrome |
|  | other partners for SARS |

---

|  |  |
| --- | --- |
| Source | curated |
|  | ResNet |
|  | GO component |

---

|  |  |
| --- | --- |
| MedScan ID | 6774 |

---

|  |  |
| --- | --- |
| LocusLink ID | 6774 |
|  | 20848 |
|  | 25125 |
|  | 68733 |
|  | 104593 |
|  | 114129 |
|  | 508541 |
|  | 282085 |
|  | 30767 |
|  | 323957 |
|  | 334563 |
|  | 793847 |
|  | 50949 |

---

|  |  |
| --- | --- |
| Alias | 1110034C02 |
|  | Signal transducer and activator of transcription III |
|  | STAT3 transcription factor |
|  | Signal transducer and activator of transcription 3 |
|  | signal transducer and activator of transcription 3 isoform 2 |
|  | signal transducer and activator of transcription 3 (acute-phase response fa |
|  | FLJ20882 |
|  | MGC16063 |
|  | OTTMUSP00000002053 |
|  | DNA-binding protein APRF |
|  | 1110034C02Rik |
|  | Hyper-IgE syndrome gene |
|  | signal transducer and activator of transcription 3 isoform II |
|  | signal transducer and activator of transcription 3 isoform 1 |
|  | RP23-279L23.5 |
|  | HIES gene |
|  | MGC93551 |
|  | APRF |
|  | Hyper-IgE syndrome protein |
|  | transcription factor STAT3 |
|  | RIKEN cDNA 1110034C02 gene |
|  | HIES protein |
|  | OTTMUSP00000045316 |
|  | OTTMUSP00000002052 |
|  | STAT3 |
|  | signal transducer and activator of transcription 3 (acute-phase response factor) |
|  | OTTMUSP00000045315 |
|  | signal transducer and activator of transcription 3 isoform I |
|  | acute phase response factor |
|  | AW109958 |
|  | ADMIO1 gene |
|  | HIES |
|  | ADMIO |
|  | ADMIO1 |
|  | acute-phase response factor |
|  | Acute-phase response factor |
|  | hypothetical protein MGC16063 |

---

|  |  |
| --- | --- |
| GO ID | 0031730 |
|  | 0003677 |
|  | 0001228 |
|  | 0003700 |
|  | 0000981 |
|  | 0000978 |
|  | 0001103 |
|  | 0031490 |
|  | 0035259 |
|  | 0004879 |
|  | 0042803 |
|  | 0019901 |
|  | 0019903 |
|  | 0008134 |
|  | 0044212 |
|  | 0007259 |
|  | 0060397 |
|  | 0072540 |
|  | 0006953 |
|  | 0007568 |
|  | 0048708 |
|  | 0008283 |
|  | 0071345 |
|  | 0032870 |
|  | 0044320 |
|  | 0071407 |
|  | 0019221 |
|  | 0006952 |
|  | 0042755 |
|  | 0097009 |
|  | 0001754 |
|  | 0042593 |
|  | 0060396 |
|  | 0006954 |
|  | 0035723 |
|  | 0038114 |
|  | 0038155 |
|  | 0070106 |
|  | 0070757 |
|  | 0070102 |
|  | 0038111 |
|  | 0038113 |
|  | 0030522 |
|  | 0033210 |
|  | 0042789 |
|  | 0035278 |
|  | 0050804 |
|  | 0043066 |
|  | 0010507 |
|  | 0008285 |
|  | 0045820 |
|  | 0010730 |
|  | 1901215 |
|  | 2001223 |
|  | 2000737 |
|  | 0000122 |
|  | 0007399 |
|  | 0016310 |
|  | 2001171 |
|  | 0051092 |
|  | 0045747 |
|  | 0045766 |
|  | 0030335 |
|  | 0045648 |
|  | 0010628 |
|  | 2000637 |
|  | 1902728 |
|  | 0045410 |
|  | 1904685 |
|  | 1902895 |
|  | 0045944 |
|  | 0045893 |
|  | 0042531 |
|  | 1905564 |
|  | 0099527 |
|  | 0006606 |
|  | 0060019 |
|  | 0051726 |
|  | 0042127 |
|  | 0060259 |
|  | 0046902 |
|  | 0040014 |
|  | 0006357 |
|  | 0006355 |
|  | 0032355 |
|  | 0045471 |
|  | 0044321 |
|  | 0043434 |
|  | 0019953 |
|  | 0007165 |
|  | 0035019 |
|  | 0001659 |
|  | 0016032 |
|  | 0090575 |
|  | 0098685 |
|  | 0005737 |
|  | 0005829 |
|  | 0098978 |
|  | 0005743 |
|  | 0000790 |
|  | 0005654 |
|  | 0005634 |
|  | 0005886 |
|  | 0014069 |
|  | 0005667 |
|  | 0043565 |
|  | 0060548 |
|  | 0008284 |
|  | 0034097 |
|  | 0019827 |
|  | 0006366 |
|  | 0005739 |
|  | 0042493 |
|  | 0014070 |
|  | 0010033 |
|  | 0006351 |
|  | 0001077 |
|  | 0005622 |
|  | 0005509 |
|  | 0005515 |
|  | 0046983 |
|  | 0048011 |
|  | 0004871 |
|  | 0019048 |
|  | 0005730 |
|  | 0006928 |
|  | 0042517 |
|  | 0016563 |
|  | 0044419 |
|  | 0055091 |
|  | 0005062 |
|  | 0045449 |
|  | 0006350 |
|  | 0007242 |

---

|  |  |
| --- | --- |
| KEGG ID | hsa:6774 |
|  | mmu:20848 |
|  | rno:25125 |

---

|  |  |
| --- | --- |
| MedLine Reference | 10323205 |
|  | 11710966 |
|  | 11799081 |
|  | 11867182 |
|  | 11872739 |
|  | 12080006 |
|  | 12087100 |
|  | 12130569 |
|  | 12153141 |
|  | 12191995 |
|  | 12242030 |
|  | 12392283 |
|  | 12477932 |
|  | 12487370 |
|  | 12494267 |
|  | 12498781 |
|  | 12528179 |
|  | 12531244 |
|  | 12584205 |
|  | 12595539 |
|  | 12695539 |
|  | 12847226 |
|  | 14678947 |
|  | 14725620 |
|  | 14767990 |
|  | 15007380 |
|  | 15060019 |
|  | 15187153 |
|  | 15256805 |
|  | 15292470 |
|  | 15322111 |
|  | 15469886 |
|  | 15476586 |
|  | 15548519 |
|  | 15590660 |
|  | 15591053 |
|  | 15716400 |
|  | 15860735 |
|  | 15893771 |
|  | 15948155 |
|  | 15948243 |
|  | 15998644 |
|  | 16025117 |
|  | 16127698 |
|  | 16225866 |
|  | 16300827 |
|  | 16306356 |
|  | 16413512 |
|  | 16514419 |
|  | 16551428 |
|  | 16565302 |
|  | 16616937 |
|  | 16764840 |
|  | 16809483 |
|  | 16931573 |
|  | 16934228 |
|  | 16971535 |
|  | 8530402 |
|  | 9716657 |
|  | 10023769 |
|  | 10077599 |
|  | 10080923 |
|  | 10200816 |
|  | 10205054 |
|  | 10349636 |
|  | 10428964 |
|  | 10458605 |
|  | 10469645 |
|  | 10521404 |
|  | 10526573 |
|  | 10675904 |
|  | 10725249 |
|  | 10781587 |
|  | 10781830 |
|  | 10851050 |
|  | 10851059 |
|  | 10851062 |
|  | 10867663 |
|  | 10922068 |
|  | 10978511 |
|  | 11013085 |
|  | 11042159 |
|  | 11076861 |
|  | 11087819 |
|  | 11089524 |
|  | 11161808 |
|  | 11180952 |
|  | 11217851 |
|  | 11238899 |
|  | 11239451 |
|  | 11275690 |
|  | 11437454 |
|  | 11520461 |
|  | 11579100 |
|  | 11591128 |
|  | 11726030 |
|  | 11735219 |
|  | 11739197 |
|  | 11764284 |
|  | 11807093 |
|  | 11812786 |
|  | 11853668 |
|  | 11861304 |
|  | 11881154 |
|  | 11889125 |
|  | 11964286 |
|  | 12032149 |
|  | 12049654 |
|  | 12057007 |
|  | 12061821 |
|  | 12084939 |
|  | 12090754 |
|  | 12093727 |
|  | 12111703 |
|  | 12147685 |
|  | 12150892 |
|  | 12193580 |
|  | 12208879 |
|  | 12219085 |
|  | 12426389 |
|  | 12438448 |
|  | 12444555 |
|  | 12466851 |
|  | 12526039 |
|  | 12540842 |
|  | 12571365 |
|  | 12594516 |
|  | 12628925 |
|  | 12634107 |
|  | 12691915 |
|  | 12727921 |
|  | 12738762 |
|  | 12743296 |
|  | 12746441 |
|  | 12748279 |
|  | 12753872 |
|  | 12810593 |
|  | 12826573 |
|  | 12832402 |
|  | 12842895 |
|  | 12907458 |
|  | 14525952 |
|  | 14527166 |
|  | 14566054 |
|  | 14623907 |
|  | 14634136 |
|  | 14636556 |
|  | 14657276 |
|  | 14660441 |
|  | 14670306 |
|  | 14673160 |
|  | 14673173 |
|  | 14688356 |
|  | 14702106 |
|  | 14716305 |
|  | 14729509 |
|  | 14729671 |
|  | 14737107 |
|  | 14764608 |
|  | 14764630 |
|  | 14975238 |
|  | 14978477 |
|  | 14996842 |
|  | 15021879 |
|  | 15037656 |
|  | 15044588 |
|  | 15069015 |
|  | 15070774 |
|  | 15188379 |
|  | 15192020 |
|  | 15194489 |
|  | 15207851 |
|  | 15208705 |
|  | 15218058 |
|  | 15226823 |
|  | 15251981 |
|  | 15254021 |
|  | 15278897 |
|  | 15284232 |
|  | 15292206 |
|  | 15306216 |
|  | 15343379 |
|  | 15343391 |
|  | 15356132 |
|  | 15358102 |
|  | 15366002 |
|  | 15467733 |
|  | 15485908 |
|  | 15541348 |
|  | 15561935 |
|  | 15572665 |
|  | 15582129 |
|  | 15607729 |
|  | 15618518 |
|  | 15632011 |
|  | 15634877 |
|  | 15640156 |
|  | 15647843 |
|  | 15650055 |
|  | 15659653 |
|  | 15664994 |
|  | 15671148 |
|  | 15673499 |
|  | 15673569 |
|  | 15688008 |
|  | 15688401 |
|  | 15694417 |
|  | 15705584 |
|  | 15730854 |
|  | 15735683 |
|  | 15735720 |
|  | 15749075 |
|  | 15749841 |
|  | 15761497 |
|  | 15761498 |
|  | 15778348 |
|  | 15781265 |
|  | 15782199 |
|  | 15790774 |
|  | 15793565 |
|  | 15838885 |
|  | 15893881 |
|  | 15895073 |
|  | 15917293 |
|  | 15919823 |
|  | 15923602 |
|  | 15936723 |
|  | 15964802 |
|  | 15970507 |
|  | 15976028 |
|  | 15978261 |
|  | 15991029 |
|  | 15994947 |
|  | 15996112 |
|  | 15998795 |
|  | 16014896 |
|  | 16026757 |
|  | 16041381 |
|  | 16046413 |
|  | 16054060 |
|  | 16107692 |
|  | 16116228 |
|  | 16141072 |
|  | 16141073 |
|  | 16141211 |
|  | 16174774 |
|  | 16192306 |
|  | 16212920 |
|  | 16230418 |
|  | 16285960 |
|  | 16286017 |
|  | 16288283 |
|  | 16289036 |
|  | 16293640 |
|  | 16298512 |
|  | 16368885 |
|  | 16377083 |
|  | 16388483 |
|  | 16417589 |
|  | 16418226 |
|  | 16452166 |
|  | 16581004 |
|  | 16601124 |
|  | 16634641 |
|  | 16636663 |
|  | 16647058 |
|  | 16679075 |
|  | 16702165 |
|  | 16709597 |
|  | 16713992 |
|  | 16717113 |
|  | 16718380 |
|  | 16720575 |
|  | 16728475 |
|  | 16737695 |
|  | 16751181 |
|  | 16783372 |
|  | 16787943 |
|  | 16788692 |
|  | 16825489 |
|  | 16829633 |
|  | 16888100 |
|  | 16939807 |
|  | 16959875 |
|  | 16971418 |
|  | 16982690 |
|  | 16988490 |
|  | 17015723 |
|  | 17023536 |
|  | 17028185 |
|  | 17030178 |
|  | 17077290 |
|  | 17078929 |
|  | 17082570 |
|  | 17095713 |
|  | 17202473 |
|  | 7512451 |
|  | 7523373 |
|  | 7545930 |
|  | 7568080 |
|  | 7579387 |
|  | 7681397 |
|  | 8140422 |
|  | 8530075 |
|  | 8626752 |
|  | 8631837 |
|  | 8670868 |
|  | 8675499 |
|  | 8692794 |
|  | 8692797 |
|  | 8782827 |
|  | 8833906 |
|  | 8889548 |
|  | 8918689 |
|  | 8942998 |
|  | 9022056 |
|  | 9108058 |
|  | 9119818 |
|  | 9287210 |
|  | 9342212 |
|  | 9464852 |
|  | 9497331 |
|  | 9630560 |
|  | 9638363 |
|  | 9695817 |
|  | 9794394 |
|  | 9874564 |
|  | 10037026 |
|  | 10446219 |
|  | 10464281 |
|  | 10490649 |
|  | 10506573 |
|  | 10521505 |
|  | 10570284 |
|  | 10602027 |
|  | 10688651 |
|  | 10764767 |
|  | 10809230 |
|  | 10825200 |
|  | 10875894 |
|  | 10878010 |
|  | 10918587 |
|  | 10925297 |
|  | 10954736 |
|  | 10982829 |
|  | 11021801 |
|  | 11134330 |
|  | 11163768 |
|  | 11171987 |
|  | 11279133 |
|  | 11294897 |
|  | 11335711 |
|  | 11350938 |
|  | 11418668 |
|  | 11429412 |
|  | 11438698 |
|  | 11463827 |
|  | 11470914 |
|  | 11536047 |
|  | 11585385 |
|  | 11594781 |
|  | 11722592 |
|  | 11751884 |
|  | 11751994 |
|  | 11773079 |
|  | 11809683 |
|  | 11815625 |
|  | 11827956 |
|  | 11843291 |
|  | 11856732 |
|  | 11859072 |
|  | 11861277 |
|  | 11861839 |
|  | 11882364 |
|  | 11923478 |
|  | 11929748 |
|  | 11940567 |
|  | 11940572 |
|  | 11959895 |
|  | 11960372 |
|  | 11987152 |
|  | 12023369 |
|  | 12039028 |
|  | 12060494 |
|  | 12061840 |
|  | 12067972 |
|  | 12070153 |
|  | 12105218 |
|  | 12168776 |
|  | 12193474 |
|  | 12235142 |
|  | 12244095 |
|  | 12359225 |
|  | 12361954 |
|  | 12389630 |
|  | 12393476 |
|  | 12396456 |
|  | 12444102 |
|  | 12444174 |
|  | 12466961 |
|  | 12506013 |
|  | 12531804 |
|  | 12545153 |
|  | 12547716 |
|  | 12551922 |
|  | 12555068 |
|  | 12556536 |
|  | 12559950 |
|  | 12562765 |
|  | 12576423 |
|  | 12600988 |
|  | 12615922 |
|  | 12623850 |
|  | 12626508 |
|  | 12629155 |
|  | 12629515 |
|  | 12637318 |
|  | 12637586 |
|  | 12640143 |
|  | 12670499 |
|  | 12707028 |
|  | 12748293 |
|  | 12763138 |
|  | 12777975 |
|  | 12782602 |
|  | 12789269 |
|  | 12804609 |
|  | 12821944 |
|  | 12833138 |
|  | 12846741 |
|  | 12865928 |
|  | 12865943 |
|  | 12867595 |
|  | 12873986 |
|  | 12900415 |
|  | 12947115 |
|  | 12960275 |
|  | 12963127 |
|  | 12969979 |
|  | 14515142 |
|  | 14522952 |
|  | 14551213 |
|  | 14593105 |
|  | 14647442 |
|  | 14672334 |
|  | 14674010 |
|  | 14688368 |
|  | 14701810 |
|  | 14702039 |
|  | 14712222 |
|  | 14715251 |
|  | 14715258 |
|  | 14736711 |
|  | 14963038 |
|  | 14966128 |
|  | 15034082 |
|  | 15064716 |
|  | 15070700 |
|  | 15077160 |
|  | 15141228 |
|  | 15143062 |
|  | 15145953 |
|  | 15150111 |
|  | 15156153 |
|  | 15161657 |
|  | 15162527 |
|  | 15163742 |
|  | 15165826 |
|  | 15184909 |
|  | 15194868 |
|  | 15198092 |
|  | 15207733 |
|  | 15223310 |
|  | 15229229 |
|  | 15242774 |
|  | 15254691 |
|  | 15277698 |
|  | 15284113 |
|  | 15286705 |
|  | 15297310 |
|  | 15336564 |
|  | 15378007 |
|  | 15465816 |
|  | 15474458 |
|  | 15476590 |
|  | 15530426 |
|  | 15575898 |
|  | 15588985 |
|  | 15590419 |
|  | 15604419 |
|  | 15615703 |
|  | 15629435 |
|  | 15643501 |
|  | 15649887 |
|  | 15650183 |
|  | 15653507 |
|  | 15665295 |
|  | 15677474 |
|  | 15682485 |
|  | 15688010 |
|  | 15703780 |
|  | 15735682 |
|  | 15735721 |
|  | 15736426 |
|  | 15764709 |
|  | 15809078 |
|  | 15837065 |
|  | 15845643 |
|  | 15870198 |
|  | 15878791 |
|  | 15882975 |
|  | 15894558 |
|  | 15905571 |
|  | 15912144 |
|  | 15935090 |
|  | 15936715 |
|  | 15944400 |
|  | 15950906 |
|  | 15958548 |
|  | 15979846 |
|  | 16007214 |
|  | 16036105 |
|  | 16061629 |
|  | 16061651 |
|  | 16081048 |
|  | 16082218 |
|  | 16098628 |
|  | 16125646 |
|  | 16140268 |
|  | 16142329 |
|  | 16192633 |
|  | 16236134 |
|  | 16236267 |
|  | 16278387 |
|  | 16331268 |
|  | 16382134 |
|  | 16407171 |
|  | 16425286 |
|  | 16432158 |
|  | 16503733 |
|  | 16512876 |
|  | 16524883 |
|  | 16557588 |
|  | 16568091 |
|  | 16571725 |
|  | 16619044 |
|  | 16636048 |
|  | 16651533 |
|  | 16709613 |
|  | 16732314 |
|  | 16807407 |
|  | 16825495 |
|  | 16828865 |
|  | 16835372 |
|  | 16840717 |
|  | 16861352 |
|  | 16871275 |
|  | 16877361 |
|  | 16887796 |
|  | 16926159 |
|  | 16935931 |
|  | 16948814 |
|  | 16959370 |
|  | 16984731 |
|  | 17008315 |
|  | 17012261 |
|  | 17015686 |
|  | 3031469 |
|  | 7624343 |
|  | 7719938 |
|  | 8272872 |
|  | 8608603 |
|  | 8626374 |
|  | 8626489 |
|  | 8631962 |
|  | 8657134 |
|  | 8921406 |
|  | 8923468 |
|  | 9162009 |
|  | 9211920 |
|  | 9343414 |
|  | 9373245 |
|  | 9388192 |
|  | 9398404 |
|  | 9440692 |
|  | 9484840 |
|  | 9566874 |
|  | 9584171 |
|  | 9647732 |
|  | 9670957 |
|  | 9714332 |
|  | 9864141 |
|  | 9872331 |
|  | 9917912 |
|  | 9923604 |
|  | 9989503 |
|  | 12419823 |
|  | 12637510 |
|  | 16257644 |
|  | 11076863 |
|  | 15194700 |
|  | 15489334 |
|  | 15489336 |
|  | 15592455 |
|  | 15951569 |
|  | 15994929 |
|  | 16381901 |
|  | 16685378 |
|  | 16841088 |
|  | 16904741 |
|  | 16946298 |
|  | 17078813 |
|  | 17142261 |
|  | 17145757 |
|  | 17151100 |
|  | 17182572 |
|  | 17194701 |
|  | 17203226 |
|  | 17204573 |
|  | 17209045 |
|  | 17220301 |
|  | 17241887 |
|  | 17276988 |
|  | 17289576 |
|  | 17311011 |
|  | 17318196 |
|  | 17341611 |
|  | 17341659 |
|  | 17344214 |
|  | 17360477 |
|  | 17374439 |
|  | 17376889 |
|  | 17379849 |
|  | 17404258 |
|  | 17438134 |
|  | 17459060 |
|  | 17460772 |
|  | 17463090 |
|  | 17531096 |
|  | 17533050 |
|  | 17543278 |
|  | 17543500 |
|  | 17545518 |
|  | 17597020 |
|  | 17602083 |
|  | 17603019 |
|  | 17616678 |
|  | 17634553 |
|  | 17676033 |
|  | 17689208 |
|  | 17824789 |
|  | 7701321 |
|  | 12704203 |
|  | 17082315 |
|  | 17089128 |
|  | 17113145 |
|  | 17131407 |
|  | 17161614 |
|  | 17198696 |
|  | 17202361 |
|  | 17204554 |
|  | 17227828 |
|  | 17233738 |
|  | 17234735 |
|  | 17241879 |
|  | 17256754 |
|  | 17277312 |
|  | 17353274 |
|  | 17360716 |
|  | 17363300 |
|  | 17363460 |
|  | 17404271 |
|  | 17452461 |
|  | 17475846 |
|  | 17485440 |
|  | 17493959 |
|  | 17509611 |
|  | 17525280 |
|  | 17562326 |
|  | 17581537 |
|  | 7543024 |
|  | 9671298 |
|  | 17031671 |
|  | 17322172 |
|  | 17326204 |
|  | 17464217 |
|  | 17487688 |
|  | 17498643 |
|  | 17611279 |
|  | 10441580 |

---

|  |  |
| --- | --- |
| Organism | Homo sapiens {Organism urn:agi-taxid:9606} |
|  | Mus musculus {Organism urn:agi-taxid:10090} |
|  | Rattus norvegicus {Organism urn:agi-taxid:10116} |
|  | Homo sapiens |
|  | Mus musculus |
|  | Rattus norvegicus |
|  | Bos taurus |
|  | Danio rerio |

---

|  |  |
| --- | --- |
| Mouse chromosome position | 11 63.82 cM |
|  | 11 60.5 cM |

---

|  |  |
| --- | --- |
| OMIM ID | 102582 |
|  | 615952 |
|  | 147060 |

---

|  |  |
| --- | --- |
| Rat chromosome position | 10q31 |
|  | 10q32.1 |

---

|  |  |
| --- | --- |
| Hugo ID | 11364 |
|  | HGNC:11364 |

---

|  |  |
| --- | --- |
| Human chromosome position | 17q21.2 |
|  | 17q21.31 |
|  | 4q21 |

---

|  |  |
| --- | --- |
| Swiss-Prot Accession | P40763 |
|  | P40763.2 |
|  | Q6GU23 |
|  | P42227 |
|  | Q3ULI4 |
|  | P42227.2 |
|  | P52631 |
|  | P52631.1 |
|  | A8K7B8 |
|  | K7ENL3 |
|  | O14916 |
|  | Q9BW54 |
|  | A2A5D1 |
|  | B7ZC17 |
|  | Q9BXH2 |
|  | Q3U5Q4 |
|  | Q3U6S9 |
|  | Q8CFJ6 |
|  | Q99ML3 |
|  | STAT3\_HUMAN |
|  | STAT3\_MOUSE |
|  | STAT3\_RAT |
|  | A2A5D1\_MOUSE |
|  | B7ZC17\_MOUSE |
|  | Q6GU23\_MOUSE |
|  | Q8N2X9 |

---

|  |  |
| --- | --- |
| PIR ID | A54444 |
|  | I49508 |

---

|  |  |
| --- | --- |
| GenBank ID | NC\_000017 |
|  | XM\_017024973 |
|  | XP\_016880462 |
|  | NM\_001369519 |
|  | NP\_001356448 |
|  | NM\_003150 |
|  | NP\_003141 |
|  | NM\_001369517 |
|  | NP\_001356446 |
|  | NM\_001369512 |
|  | NP\_001356441 |
|  | NM\_001369514 |
|  | NP\_001356443 |
|  | NM\_001369518 |
|  | NP\_001356447 |
|  | NM\_001369513 |
|  | NP\_001356442 |
|  | NM\_001369520 |
|  | NP\_001356449 |
|  | NM\_001369516 |
|  | NP\_001356445 |
|  | NM\_213662 |
|  | NP\_998827 |
|  | NM\_139276 |
|  | NP\_644805 |
|  | XM\_024450896 |
|  | XP\_024306664 |
|  | NG\_007370 |
|  | AC087691 |
|  | AC107993 |
|  | AF332508 |
|  | AAK17196 |
|  | AY572796 |
|  | AAS66986 |
|  | CH471152 |
|  | EAW60820 |
|  | EAW60821 |
|  | EAW60822 |
|  | EAW60823 |
|  | EAW60824 |
|  | EAW60825 |
|  | EAW60826 |
|  | EAW60827 |
|  | HI961665 |
|  | CBY84972 |
|  | JA362592 |
|  | CCA94578 |
|  | JX296640 |
|  | JX296641 |
|  | JX296642 |
|  | JX296643 |
|  | JX296644 |
|  | JX296645 |
|  | JX296646 |
|  | JX296647 |
|  | JX296648 |
|  | JX296649 |
|  | JX296650 |
|  | JX296651 |
|  | JX296652 |
|  | JX296653 |
|  | JX296654 |
|  | JX296655 |
|  | JX296656 |
|  | JX296657 |
|  | JX296658 |
|  | JX296659 |
|  | JX296660 |
|  | JX296661 |
|  | JX296662 |
|  | JX296663 |
|  | JX296664 |
|  | JX296665 |
|  | JX296666 |
|  | JX296667 |
|  | JX296668 |
|  | JX296669 |
|  | JX296670 |
|  | JX296671 |
|  | JX296672 |
|  | JX296673 |
|  | JX296674 |
|  | JX296675 |
|  | JX296676 |
|  | JX296677 |
|  | JX296678 |
|  | JX296679 |
|  | AB451232 |
|  | BAG70046 |
|  | AF029311 |
|  | AAB84254 |
|  | AI631896 |
|  | AJ012463 |
|  | CAA10032 |
|  | AK024535 |
|  | AK092965 |
|  | AK291933 |
|  | BAF84622 |
|  | AK297994 |
|  | BAG60302 |
|  | AK301200 |
|  | BAG62778 |
|  | AK316139 |
|  | BAH14510 |
|  | BC000627 |
|  | AAH00627 |
|  | BC008044 |
|  | BC014482 |
|  | AAH14482 |
|  | BC029783 |
|  | BC067119 |
|  | BC107775 |
|  | BI461226 |
|  | CB216860 |
|  | CB321646 |
|  | CF454565 |
|  | L29277 |
|  | AAA58374 |
|  | P40763 |
|  | NC\_000077 |
|  | NM\_213660 |
|  | NP\_998825 |
|  | NM\_011486 |
|  | NP\_035616 |
|  | NM\_213659 |
|  | NP\_998824 |
|  | XM\_011248846 |
|  | XP\_011247148 |
|  | XM\_017314401 |
|  | XP\_017169890 |
|  | AB008160 |
|  | AF246978 |
|  | AAL59017 |
|  | AF332507 |
|  | AAK17195 |
|  | AL591466 |
|  | DS033677 |
|  | EDL01234 |
|  | AA123454 |
|  | AK004083 |
|  | AK079406 |
|  | AK145486 |
|  | BAE26464 |
|  | AK153005 |
|  | BAE31645 |
|  | AK153170 |
|  | AK153472 |
|  | BAE32023 |
|  | AK161906 |
|  | AK184281 |
|  | AK192932 |
|  | AK192951 |
|  | AK193746 |
|  | AK197148 |
|  | AY299489 |
|  | AAQ75418 |
|  | AY299490 |
|  | AAQ75419 |
|  | BC003806 |
|  | AAH03806 |
|  | BC019168 |
|  | AAH19168 |
|  | BC037688 |
|  | AAH37688 |
|  | BY301717 |
|  | L29278 |
|  | AAA37254 |
|  | U06922 |
|  | AAA19452 |
|  | U08378 |
|  | AAA56668 |
|  | U30709 |
|  | AAC52612 |
|  | P42227 |
|  | NC\_005109 |
|  | XM\_006247259 |
|  | XP\_006247321 |
|  | XM\_006247257 |
|  | XP\_006247319 |
|  | XM\_006247258 |
|  | XP\_006247320 |
|  | NM\_012747 |
|  | NP\_036879 |
|  | AC\_000078 |
|  | AAHX01066019 |
|  | AC117979 |
|  | CH473948 |
|  | EDM06069 |
|  | BC087025 |
|  | AAH87025 |
|  | FQ223504 |
|  | FQ225489 |
|  | GU477503 |
|  | GU477504 |
|  | X91810 |
|  | CAA62920 |
|  | P52631 |
|  | XM\_017024972 |
|  | XM\_017024976 |
|  | XM\_017024974 |
|  | XM\_017024975 |
|  | XP\_005257673 |
|  | XP\_005257674 |
|  | XP\_011523447 |
|  | XP\_011523448 |
|  | XM\_011525146 |
|  | XM\_011525145 |
|  | XP\_016880463 |
|  | XP\_016880461 |
|  | XP\_016880465 |
|  | XP\_016880464 |
|  | XM\_005257616 |
|  | XM\_005257617 |
|  | NW\_001072672 |
|  | ABZ92186 |
|  | AAHY01199405 |
|  | AC\_000149 |
|  | XP\_005257677 |
|  | XP\_005257670 |
|  | XP\_005257671 |
|  | XP\_005257672 |
|  | XP\_005257675 |
|  | XP\_005257676 |
|  | AC\_000033 |
|  | AAHY01149956 |
|  | ABBA01006426 |
|  | ABBA01006427 |
|  | AABR06065579 |
|  | ABBA01006428 |
|  | AABR06065578 |
|  | NC\_018928 |
|  | ACE86450 |
|  | AM393108 |
|  | CAM19460 |
|  | CAM19461 |
|  | AMYH02033429 |
|  | NW\_001076646 |
|  | ACE87126 |
|  | AMYH02033428 |
|  | EU831531 |
|  | EU446657 |
|  | EU831439 |
|  | XM\_005257613 |
|  | CAX15621 |
|  | CAX15620 |
|  | XM\_005257614 |
|  | XM\_005257615 |
|  | XM\_005257618 |
|  | XM\_005257619 |
|  | AAHY01095762 |
|  | CAL37986 |
|  | XM\_005257620 |
|  | NT\_010783 |
|  | NW\_001838436 |
|  | NW\_926828 |
|  | AC\_000060 |
|  | Q9BXH2 |
|  | NT\_165773 |
|  | NW\_001030432 |
|  | Q3U5Q4 |
|  | Q3U6S9 |
|  | Q3ULI4 |
|  | Q6GU23 |
|  | Q8CFJ6 |
|  | Q99ML3 |
|  | NW\_047339 |
|  | NW\_001084656 |
|  | NT\_096143 |
|  | NT\_010755 |
|  | AAH29783 |
|  | Q8N2X9 |
|  | NT\_096135 |
|  | XP\_001005155 |
|  | XM\_907398 |
|  | AI325183 |
|  | XM\_001005155 |
|  | BI079838 |
|  | BG076355 |
|  | NW\_000040 |
|  | 1107849 |
|  | 1711554 |
|  | 56268829 |
|  | 1166506 |
|  | 123794241 |
|  | 123794262 |
|  | 123794726 |
|  | 13272530 |
|  | 13277852 |
|  | 1711553 |
|  | 17512414 |
|  | 18087726 |
|  | 22902299 |
|  | 34559408 |
|  | 34559410 |
|  | 458706 |
|  | 473890 |
|  | 476716 |
|  | 74151969 |
|  | 74211440 |
|  | 74225465 |
|  | 81867865 |
|  | 81878009 |
|  | 81886328 |
|  | AW109958 |
|  | 119581226 |
|  | 119581227 |
|  | 119581228 |
|  | 12653685 |
|  | 13272532 |
|  | 15680254 |
|  | 20987437 |
|  | 2613014 |
|  | 3850050 |
|  | 45505190 |
|  | 475789 |
|  | 48429227 |
|  | 74717617 |
|  | 74728735 |
|  | NT\_086877 |
|  | XP\_912491 |
|  | 44804293 |
|  | 44804295 |
|  | 44804297 |
|  | 44804303 |
|  | 44804305 |
|  | 44804309 |
|  | 44804311 |
|  | 44804313 |
|  | 44804317 |
|  | 48428571 |
|  | 8249012 |
|  | AJ276489 |
|  | AJ620655 |
|  | AJ620656 |
|  | AJ620657 |
|  | AJ620660 |
|  | AJ620661 |
|  | AJ620663 |
|  | AJ620664 |
|  | AJ620665 |
|  | AJ620667 |
|  | CAB93140 |
|  | CAF06182 |
|  | CAF06183 |
|  | CAF06184 |
|  | CAF06187 |
|  | CAF06188 |
|  | CAF06190 |
|  | CAF06191 |
|  | CAF06192 |
|  | CAF06194 |
|  | P61635 |
|  | 122143993 |
|  | 44804299 |
|  | 44804301 |
|  | 44804307 |
|  | 44804315 |
|  | 75044935 |
|  | 75044936 |
|  | 75044937 |
|  | 75044938 |
|  | 75044939 |
|  | 75044940 |
|  | 75044941 |
|  | 75044942 |
|  | 75044943 |
|  | 75044944 |
|  | 75050657 |
|  | 75053153 |
|  | 81674209 |
|  | AAI09482 |
|  | AJ620658 |
|  | AJ620659 |
|  | AJ620662 |
|  | AJ620666 |
|  | BC109481 |
|  | CAF06185 |
|  | CAF06186 |
|  | CAF06189 |
|  | CAF06193 |
|  | NC\_007317 |
|  | NM\_001012671 |
|  | NP\_001012689 |
|  | NW\_001493680 |
|  | Q32LP6 |
|  | Q704V9 |
|  | Q704W0 |
|  | Q704W1 |
|  | Q704W2 |
|  | Q704W3 |
|  | Q704W4 |
|  | Q704W5 |
|  | Q704W6 |
|  | Q704W7 |
|  | Q704W8 |
|  | Q704W9 |
|  | Q9N0J4 |
|  | AI641458 |
|  | AI657582 |
|  | BF157016 |
|  | BG307556 |
|  | NC\_007114 |
|  | NW\_001513072 |
|  | XM\_001333647 |
|  | XP\_001333683 |
|  | 117645040 |
|  | 119581224 |
|  | 119581225 |
|  | 119581229 |
|  | 119581230 |
|  | 119581231 |
|  | 123239571 |
|  | 123239572 |
|  | 148669287 |
|  | 149054252 |
|  | P40763-2 |

---

|  |  |
| --- | --- |
| Swiss-Prot ID | STAT3\_HUMAN |
|  | STAT3\_MOUSE |
|  | STAT3\_RAT |
|  | Q6GU23\_MOUSE |
|  | A2A5D1\_MOUSE |
|  | B7ZC17\_MOUSE |

---

|  |  |
| --- | --- |
| Cell Localization | Cytoplasm |
|  | Nucleus |

---

|  |  |
| --- | --- |
| Ensembl ID | ENSG00000168610 |
|  | ENSP00000384943.3 |
|  | ENST00000404395.3 |
|  | ENSP00000264657.4 |
|  | ENST00000264657.9 |
|  | ENSMUSG00000004040 |
|  | ENSMUSP00000099403.1 |
|  | ENSMUST00000103114.7 |
|  | ENSMUSP00000120152.1 |
|  | ENSMUST00000127638.7 |
|  | ENSMUSP00000090342.5 |
|  | ENSMUST00000092671.11 |
|  | ENSRNOG00000019742 |
|  | ENSRNOP00000026760.3 |
|  | ENSRNOT00000026760.4 |
|  | ENSP00000467985.1 |
|  | ENSP00000467000.1 |
|  | ENST00000588969.5 |
|  | ENST00000585517.5 |
|  | ENSMUSP00000090342 |
|  | ENSMUSP00000120152 |
|  | ENSP00000384943 |
|  | ENSMUST00000103114 |
|  | ENSP00000467000 |
|  | ENSP00000467985 |
|  | ENSRNOP00000026760 |
|  | ENST00000404395 |
|  | ENSP00000264657 |
|  | ENSMUSP00000099403 |
|  | ENSMUST00000092671 |
|  | ENST00000264657 |
|  | ENST00000585517 |
|  | ENST00000588969 |
|  | ENSRNOT00000026760 |
|  | ENSMUST00000127638 |

---

|  |  |
| --- | --- |
| MGI ID | MGI:103038 |
|  | 103038 |
|  | 1915983 |
|  | 2144497 |

---

|  |  |
| --- | --- |
| RGD ID | 3772 |

---

|  |  |
| --- | --- |
| Unigene ID | Rn.10247 |
|  | Hs.463059 |
|  | Mm.473190 |
|  | Mm.249934 |
|  | Mm.277403 |
|  | Hs.643543 |
|  | Hs.567649 |
|  | Bt.15334 |

---

|  |  |
| --- | --- |
| Homologene ID | 7960 |

---

|  |  |
| --- | --- |
| Shape | O-vertex |

---

|  |  |
| --- | --- |
| IPI ID | IPI00753792 |
|  | IPI00306436 |
|  | IPI00227814 |
|  | IPI00228955 |
|  | IPI00208224 |
|  | IPI00784414 |
|  | IPI00412752 |

---

|  |  |
| --- | --- |
| Microarray ID | 99099\_at |
|  | 99100\_at |
|  | 208992\_s\_at |

---

|  |  |
| --- | --- |
| Homo sapiens Chromosome position | 17q21.31 |
|  | 4q21 |

---

|  |  |
| --- | --- |
| Ensembl Transcript ID | ENST00000389272 |
|  | ENST00000264657 |
|  | ENST00000404395 |

---

|  |  |
| --- | --- |
| ENSEMBL | ENSG00000168610 |

---

|  |  |
| --- | --- |
| FunctionalClass | DNA binding |
|  | hematopoietin/interferon-class (D200-domain) cytokine receptor signal transducer |
|  | protein binding |
|  | protein dimerization |
|  | transcription factor |
|  | transcription factor binding |
|  | transcriptional activator |
|  | calcium ion binding |
|  | signal transducer |
|  | transcription activator |

---

|  |  |
| --- | --- |
| Bos taurus Chromosome position | 19 |

---

|  |  |
| --- | --- |
| Mus musculus Chromosome position | 11 60.5 cM |
|  | 11 |

---

|  |  |
| --- | --- |
| KEGG pathway | Jak-STAT signaling pathway |

---

|  |  |
| --- | --- |
| ProteinAtlas SubcellularLocation | Main location: Nucleus but not nucleoli;Cytoplasm; Other location: ; Expression type: Staining; Reliability: Supportive |

---

|  |  |
| --- | --- |
| Rattus norvegicus Chromosome position | 10q32.1 |

---

|  |  |
| --- | --- |
| Ensembl Gene ID | ENSG00000168610 |

---

|  |  |
| --- | --- |
| ProteinAtlas NormalTissue | Tissue: fallopian tube; Cell type: glandular cells; Level: High; Expression type: APE; Reliability: High |
|  | Tissue: kidney; Cell type: cells in tubules; Level: Medium; Expression type: APE; Reliability: High |
|  | Tissue: lung; Cell type: pneumocytes; Level: High; Expression type: APE; Reliability: High |
|  | Tissue: stomach |
|  | lower; Cell type: glandular cells; Level: Medium; Expression type: APE; Reliability: High |
|  | Tissue: spleen; Cell type: cells in white pulp; Level: None; Expression type: APE; Reliability: High |
|  | Tissue: uterus |
|  | pre-menopause; Cell type: glandular cells; Level: Low; Expression type: APE; Reliability: High |
|  | Tissue: tonsil; Cell type: squamous epithelial cells; Level: Medium; Expression type: APE; Reliability: High |
|  | Tissue: prostate; Cell type: glandular cells; Level: Medium; Expression type: APE; Reliability: High |
|  | Tissue: seminal vesicle; Cell type: glandular cells; Level: Low; Expression type: APE; Reliability: High |
|  | Tissue: kidney; Cell type: cells in glomeruli; Level: Medium; Expression type: APE; Reliability: High |
|  | Tissue: oral mucosa; Cell type: squamous epithelial cells; Level: Medium; Expression type: APE; Reliability: High |
|  | Tissue: vulva/anal skin; Cell type: epidermal cells; Level: Medium; Expression type: APE; Reliability: High |
|  | Tissue: ovary; Cell type: ovarian stroma cells; Level: Low; Expression type: APE; Reliability: High |
|  | Tissue: skeletal muscle; Cell type: myocytes; Level: Medium; Expression type: APE; Reliability: High |
|  | Tissue: adrenal gland; Cell type: glandular cells; Level: Medium; Expression type: APE; Reliability: High |
|  | Tissue: appendix; Cell type: glandular cells; Level: Medium; Expression type: APE; Reliability: High |
|  | Tissue: appendix; Cell type: lymphoid tissue; Level: Medium; Expression type: APE; Reliability: High |
|  | Tissue: bone marrow; Cell type: hematopoietic cells; Level: Low; Expression type: APE; Reliability: High |
|  | Tissue: breast; Cell type: glandular cells; Level: Medium; Expression type: APE; Reliability: High |
|  | Tissue: bronchus; Cell type: respiratory epithelial cells; Level: Medium; Expression type: APE; Reliability: High |
|  | Tissue: cerebellum; Cell type: cells in granular layer; Level: Low; Expression type: APE; Reliability: High |
|  | Tissue: cerebellum; Cell type: cells in molecular layer; Level: Low; Expression type: APE; Reliability: High |
|  | Tissue: cerebellum; Cell type: Purkinje cells; Level: Low; Expression type: APE; Reliability: High |
|  | Tissue: cerebral cortex; Cell type: glial cells; Level: Low; Expression type: APE; Reliability: High |
|  | Tissue: cerebral cortex; Cell type: neuronal cells; Level: Low; Expression type: APE; Reliability: High |
|  | Tissue: cervix, uterine; Cell type: glandular cells; Level: Low; Expression type: APE; Reliability: High |
|  | Tissue: cervix, uterine; Cell type: squamous epithelial cells; Level: Low; Expression type: APE; Reliability: High |
|  | Tissue: colon; Cell type: glandular cells; Level: Medium; Expression type: APE; Reliability: High |
|  | Tissue: duodenum; Cell type: glandular cells; Level: Medium; Expression type: APE; Reliability: High |
|  | Tissue: epididymis; Cell type: glandular cells; Level: Medium; Expression type: APE; Reliability: High |
|  | Tissue: esophagus; Cell type: squamous epithelial cells; Level: Medium; Expression type: APE; Reliability: High |
|  | Tissue: gall bladder; Cell type: glandular cells; Level: Medium; Expression type: APE; Reliability: High |
|  | Tissue: heart muscle; Cell type: myocytes; Level: Low; Expression type: APE; Reliability: High |
|  | Tissue: hippocampus; Cell type: glial cells; Level: Low; Expression type: APE; Reliability: High |
|  | Tissue: hippocampus; Cell type: neuronal cells; Level: Low; Expression type: APE; Reliability: High |
|  | Tissue: lateral ventricle; Cell type: glial cells; Level: Low; Expression type: APE; Reliability: High |
|  | Tissue: lateral ventricle; Cell type: neuronal cells; Level: Low; Expression type: APE; Reliability: High |
|  | Tissue: liver; Cell type: bile duct cells; Level: Low; Expression type: APE; Reliability: High |
|  | Tissue: liver; Cell type: hepatocytes; Level: None; Expression type: APE; Reliability: High |
|  | Tissue: lung; Cell type: macrophages; Level: Medium; Expression type: APE; Reliability: High |
|  | Tissue: lymph node; Cell type: germinal center cells; Level: Medium; Expression type: APE; Reliability: High |
|  | Tissue: lymph node; Cell type: non-germinal center cells; Level: Low; Expression type: APE; Reliability: High |
|  | Tissue: nasopharynx; Cell type: respiratory epithelial cells; Level: Medium; Expression type: APE; Reliability: High |
|  | Tissue: pancreas; Cell type: exocrine glandular cells; Level: Medium; Expression type: APE; Reliability: High |
|  | Tissue: pancreas; Cell type: islets of Langerhans; Level: Medium; Expression type: APE; Reliability: High |
|  | Tissue: parathyroid gland; Cell type: glandular cells; Level: Medium; Expression type: APE; Reliability: High |
|  | Tissue: placenta; Cell type: decidual cells; Level: Medium; Expression type: APE; Reliability: High |
|  | Tissue: placenta; Cell type: trophoblastic cells; Level: Low; Expression type: APE; Reliability: High |
|  | Tissue: rectum; Cell type: glandular cells; Level: Medium; Expression type: APE; Reliability: High |
|  | Tissue: salivary gland; Cell type: glandular cells; Level: Low; Expression type: APE; Reliability: High |
|  | Tissue: skin; Cell type: epidermal cells; Level: Medium; Expression type: APE; Reliability: High |
|  | Tissue: small intestine; Cell type: glandular cells; Level: Medium; Expression type: APE; Reliability: High |
|  | Tissue: smooth muscle; Cell type: smooth muscle cells; Level: None; Expression type: APE; Reliability: High |
|  | Tissue: spleen; Cell type: cells in red pulp; Level: Low; Expression type: APE; Reliability: High |
|  | Tissue: stomach, lower; Cell type: glandular cells; Level: Medium; Expression type: APE; Reliability: High |
|  | Tissue: stomach, upper; Cell type: glandular cells; Level: Medium; Expression type: APE; Reliability: High |
|  | Tissue: testis; Cell type: cells in seminiferus ducts; Level: Medium; Expression type: APE; Reliability: High |
|  | Tissue: testis; Cell type: Leydig cells; Level: Medium; Expression type: APE; Reliability: High |
|  | Tissue: thyroid gland; Cell type: glandular cells; Level: Medium; Expression type: APE; Reliability: High |
|  | Tissue: tonsil; Cell type: germinal center cells; Level: Medium; Expression type: APE; Reliability: High |
|  | Tissue: tonsil; Cell type: non-germinal center cells; Level: Medium; Expression type: APE; Reliability: High |
|  | Tissue: urinary bladder; Cell type: urothelial cells; Level: Medium; Expression type: APE; Reliability: High |
|  | Tissue: uterus, post-menopause; Cell type: cells in endometrial stroma; Level: Medium; Expression type: APE; Reliability: High |
|  | Tissue: uterus, post-menopause; Cell type: glandular cells; Level: Low; Expression type: APE; Reliability: High |
|  | Tissue: uterus, pre-menopause; Cell type: cells in endometrial stroma; Level: Medium; Expression type: APE; Reliability: High |
|  | Tissue: uterus, pre-menopause; Cell type: glandular cells; Level: Low; Expression type: APE; Reliability: High |
|  | Tissue: vagina; Cell type: squamous epithelial cells; Level: Medium; Expression type: APE; Reliability: High |

---

|  |  |
| --- | --- |
| LocatorString | gscquery:curcumin+prostate+cancer |
|  | pmftq:achondroplasia |
|  | pmquery:egfr+transactivation |

---

|  |  |
| --- | --- |
| Danio rerio Chromosome position | LG 3 |

---
