# Supplementary material for: Analysis of PPARγ Signaling Activity in Psoriasis
Source: Int J Mol Sci. 2021 Aug 10;22(16):8603. doi: 10.3390/ijms22168603 (PMC8395241; doi:10.3390/ijms22168603)
Supplement: Supplementary file 1 [file ijms-22-08603-s001.zip › Supplemental materials_Analysis of PPARg signaling activity in psoriasis/Pathway models/Models images and html files/Anti-psoriatic drugs influence PPARG signaling/112005.html]

corticosteroid


# Small Molecule corticosteroid

|  |  |
| --- | --- |
| URN | urn:agi-smol:corticosteroids |
| Total Entities | 8 |
| Connectivity | 7178 |
| Name | corticosteroid |
| Class | Endogenous compound |

---

|  |  |
| --- | --- |
| ChildConcepts | glucocorticoid |
|  | mineralocorticoid |
|  | 11-hydroxycorticosteroid |
|  | 17-hydroxycorticosteroid |
|  | corticosteroid derivative |
|  | 17-hydroxysteroid |
|  | benzodrocortisone |
|  | 16alpha-hydroxycorticosterone |

---

|  |  |
| --- | --- |
| Pathway | Vascular Endothelial Cell Activation by Cytokines |
|  | CRH -> Synthesis of Corticosteroids |
|  | Anti-psoriatic drugs influence PPARG signaling |

---

|  |  |
| --- | --- |
| MedScan ID | 1800006 |

---

|  |  |
| --- | --- |
| Alias | adreno corticoid |
|  | adrenocortical hormone |
|  | adrenocorticosteroid |
|  | adrenocorticoids |
|  | cortico steroid |
|  | adrenal cortex hormone |
|  | adrenocortical steroid |
|  | corticosteroid hormone |
|  | cortical steroid |
|  | fluorinated corticosteroid |
|  | adrenal cortical hormones |
|  | [3H] corticosteroid |
|  | adreno corticoids |
|  | corticosteroids |
|  | adrenocorticoid |
|  | adrenal steroid |
|  | adrenal cortical steroid |
|  | adreno cortical steroid |
|  | corticoid |
|  | adreno corticosteroid |
|  | corticosteroid agents |
|  | adrenal corticoids |
|  | corticosteroid calcium |
|  | corticosteroid |
|  | adrenal cortex hormones |
|  | adrenal corticoid |
|  | adrenal steroid hormone |
|  | dermocorticosteroid |
|  | adrenal cortical hormone |
|  | corticosteroid agent |

---

|  |  |
| --- | --- |
| PharmaPendium ID | Corticosteroids |

---
