# Supplementary material for: Analysis of PPARγ Signaling Activity in Psoriasis
Source: Int J Mol Sci. 2021 Aug 10;22(16):8603. doi: 10.3390/ijms22168603 (PMC8395241; doi:10.3390/ijms22168603)
Supplement: Supplementary file 1 [file ijms-22-08603-s001.zip › Supplemental materials_Analysis of PPARg signaling activity in psoriasis/Pathway models/Models images and html files/Anti-psoriatic drugs influence PPARG signaling/12548.html]

FOSL1


# Protein FOSL1

|  |  |
| --- | --- |
| URN | urn:agi-llid:8061 |
| Total Entities | 0 |
| Connectivity | 1352 |
| Name | FOSL1 |
| Description | FOS-like antigen 1 |
| Notes | The Fos gene family consists of 4 members: FOS, FOSB, FOSL1, and FOSL2. These genes encode leucine zipper proteins that can dimerize with proteins of the JUN family, thereby forming the transcription factor complex AP-1. As such, the FOS proteins have been implicated as regulators of cell proliferation, differentiation, and transformation. Several transcript variants encoding different isoforms have been found for this gene. [provided by RefSeq, Jul 2014] |
| Primary Cell Localization | Nucleus |
| Class | Transcription factor |

---

|  |  |
| --- | --- |
| Pathway | Kidney Cells Cilia Organization |
|  | Proteins Involved in Atherosclerosis |
|  | X-Linked Ichthyosis |
|  | Proteins Involved in non-Alcoholic Fatty Liver Disease |
|  | Osteoclast Activation in Multiple Myeloma |
|  | EGF -> CTNN Expression Targets |
|  | EGF -> AP-1/ATF Expression Targets |
|  | EGF -> CREB/CREBBP/ELK/SRF/MYC Expression Targets |
|  | EGF -> MEF/MYOD/NFATC Expression Targets |
|  | EGF -> STAT Expression Targets |
|  | F2 -> AP-1/CREB/ELK/SRF/SP1 Expression Targets |
|  | F2 -> STAT1/NF-kB Expression Targets |
|  | FGF2 -> STAT Expression Targets |
|  | FGF2 -> AP-1/CREB/CREBBP/ELK/SRF/MYC Expression Targets |
|  | HGF -> STAT Expression Targets |
|  | HGF -> AP-1/CREB/ELK/SRF/MYC Expression Targets |
|  | Insulin -> STAT Expression Targets |
|  | Insulin -> CEBPA/CTNNB/FOXA/FOXO Expression Targets |
|  | Insulin -> MEF/MYOD Expression Targets |
|  | Insulin -> ELK/SRF/HIF1A/MYC/SREBF Expression Targets |
|  | LPA Expression Targets |
|  | CSF1 -> STAT Expression Targets |
|  | CSF1 -> AP-1/CREB/CREBBP/MYC Expression Targets |
|  | ERK5/MAPK7 Signaling |
|  | TGFA -> STAT Expression Targets |
|  | TGFA -> CTNNB/CTNND Expression Targets |
|  | TGFA/AP-1/ATF Expression Targets |
|  | TGFA -> CREB/CREBBP/ELK-SRF/MYC Expression Targets |
|  | TNF -> AP-1 Expression Targets |
|  | prarg negative regulators, ps-positive |
|  | prarg neg,ukn expres targets, ps-positive |
|  | PPARG negative regulators |
|  | PPARG negative targets |
|  | pparg expr target\_regulators |
|  | Neighbors of tumor progression |
|  | Neighbors of cancer progression |
|  | Neighbors of inflammatory status |
|  | Neighbors of overall survival |
|  | Neighbors of tumor size |
|  | Neighbors of mortality |
|  | Neighbors of clinical stage |
|  | Neighbors of lethality |
|  | Neighbors of disease-free survival |
|  | Neighbors of bone mass |
|  | Neighbors of tumor promotion |
|  | Neighbors of mortality rate |
|  | Neighbors of radioresistance |
|  | Neighbors of shear stress |
|  | Neighbors of gastric cancer risk |
|  | Neighbors of bone volume |
|  | Neighbors of tumor progression |
|  | Neighbors of cancer progression |
|  | Neighbors of inflammatory status |
|  | Neighbors of overall survival |
|  | Neighbors of tumor size |
|  | Neighbors of mortality |
|  | Neighbors of clinical stage |
|  | Neighbors of lethality |
|  | Neighbors of disease-free survival |
|  | Neighbors of bone mass |
|  | Neighbors of tumor promotion |
|  | Neighbors of mortality rate |
|  | Neighbors of radioresistance |
|  | Neighbors of shear stress |
|  | Neighbors of gastric cancer risk |
|  | Neighbors of bone volume |
|  | Neighbors of cell development |
|  | Neighbors of immune response |
|  | Neighbors of inflammatory response |
|  | Neighbors of wound healing |
|  | Neighbors of angiogenesis |
|  | Neighbors of cell function |
|  | Neighbors of cell formation |
|  | Neighbors of osteoclast development |
|  | Neighbors of epithelial to mesenchymal transition |
|  | Neighbors of immunity |
|  | Neighbors of cell survival |
|  | Neighbors of osteoclast differentiation |
|  | Neighbors of cell phenotype |
|  | Neighbors of cell adhesion |
|  | Neighbors of tumor growth |
|  | Neighbors of cell count |
|  | Neighbors of lumen formation |
|  | Neighbors of endothelial cell migration |
|  | Neighbors of adipogenesis |
|  | Neighbors of cell damage |
|  | Neighbors of aging |
|  | Neighbors of cell motility |
|  | Neighbors of bone resorption |
|  | Neighbors of adipocyte differentiation |
|  | Neighbors of cell migration |
|  | Neighbors of cancer growth |
|  | Neighbors of cell invasion |
|  | Neighbors of ossification |
|  | Neighbors of transcription activation |
|  | Neighbors of senescence |
|  | Neighbors of cell transdifferentiation |
|  | Neighbors of stem cell differentiation |
|  | Neighbors of cell growth |
|  | Neighbors of ROS generation |
|  | Neighbors of epithelial cell proliferation |
|  | Neighbors of first trimester pregnancy |
|  | Neighbors of cancer cell growth |
|  | Neighbors of macrophage function |
|  | Neighbors of cellular immune response |
|  | Neighbors of colony formation |
|  | Neighbors of cell differentiation |
|  | Neighbors of cell homeostasis |
|  | Neighbors of psoriasis |
|  | Neighbors of atherosclerosis |
|  | Neighbors of fibrosis |
|  | Neighbors of arthritis |
|  | Neighbors of inflammatory bowel disease |
|  | Neighbors of injury |
|  | Neighbors of metastasis |
|  | Neighbors of carcinogenesis |
|  | Neighbors of inflammation |
|  | Neighbors of obesity |
|  | Neighbors of pneumonia |
|  | Neighbors of osteoarthritis |
|  | Neighbors of liver fibrosis |
|  | Neighbors of leukocyte infiltration |
|  | Neighbors of liver injury |
|  | Neighbors of sepsis |
|  | Neighbors of death |
|  | Neighbors of lung injury |
|  | Neighbors of hepatocellular carcinoma |
|  | Neighbors of acute lung injury |
|  | Neighbors of gastric cancer |
|  | Neighbors of colorectal cancer |
|  | Neighbors of breast cancer |
|  | Neighbors of melanoma |
|  | Neighbors of collagen-induced arthritis |
|  | Neighbors of neoplasm invasion |
|  | Neighbors of heart failure |
|  | Neighbors of pulmonary fibrosis |
|  | Neighbors of cancer |
|  | Neighbors of neoplasm |
|  | Neighbors of prostate cancer |
|  | Neighbors of osteolysis |
|  | Neighbors of adipocyte |
|  | Neighbors of lymph node |
|  | Neighbors of NF-kB family |
|  | Neighbors of JNK |
|  | Neighbors of IL1 family |
|  | Neighbors of Jun/Fos |
|  | Neighbors of PKC |
|  | Neighbors of PI3K |
|  | Neighbors of STAT family |
|  | Neighbors of mitogen-activated protein kinase |
|  | Neighbors of TLR |
|  | Neighbors of cytokine |
|  | Neighbors of JAK |
|  | Neighbors of MEK1/2 |
|  | Neighbors of PDGF |
|  | Neighbors of Ras GTPase |
|  | Neighbors of ERK1/2 |
|  | Neighbors of growth factor |
|  | Neighbors of IKBK |
|  | Neighbors of protein tyrosine kinase |
|  | Neighbors of LXR |
|  | Neighbors of p38 |
|  | Neighbors of PDGF-BB |
|  | Neighbors of endothelin |
|  | Neighbors of proteasome endopeptidase complex |
|  | Neighbors of class nuclear receptor with C4 zinc fingers |
|  | Neighbors of WNT |
|  | Neighbors of TORC1 |
|  | Neighbors of PDGF receptor |
|  | comon ps\_pos, pprarg\_neg targets |
|  | Model of PPARG signaling in psoriasis |
|  | PPARG negative regulators and targets |
|  | Model of PPARG related pathways in psoriasis (short version) |
|  | 2\_Harlequin ichthyosis overview |
|  | Model of PPARG signaling in psoriais (tested) |
|  | before laser treatment |
|  | Anti-psoriatic drugs influence PPARG signaling |
|  | PPARG signaling after laser treatment |

---

|  |  |
| --- | --- |
| MedScan ID | 8061 |

---

|  |  |
| --- | --- |
| LocusLink ID | 8061 |
|  | 14283 |
|  | 25445 |

---

|  |  |
| --- | --- |
| Alias | transcription factor Fra-1 |
|  | fos-like antigen 1 |
|  | FOSL I |
|  | FOS like 1, AP-1 transcription factor subunit |
|  | fos-like antigen I |
|  | AW538199 |
|  | FOS-related antigen 1 |
|  | Fra-1/AP-1 transcription factor |
|  | FOSL1 |
|  | FRA I |
|  | FRA1 |
|  | FRA1\_HUMAN |
|  | FOS-related antigen I |
|  | Fra-1/AP-1 |
|  | FRA |
|  | fra-1 |
|  | FOS like 1, AP-1 trancription factor subunit |
|  | FOS-like antigen-1 |

---

|  |  |
| --- | --- |
| GO ID | 0001228 |
|  | 0003700 |
|  | 0000981 |
|  | 0000978 |
|  | 0006968 |
|  | 0031668 |
|  | 0006935 |
|  | 0007565 |
|  | 0001701 |
|  | 0007612 |
|  | 0008285 |
|  | 0060674 |
|  | 0051091 |
|  | 2000144 |
|  | 0043065 |
|  | 0045787 |
|  | 0008284 |
|  | 0045944 |
|  | 0061614 |
|  | 0006357 |
|  | 0051591 |
|  | 0051412 |
|  | 0034097 |
|  | 0009629 |
|  | 0042542 |
|  | 0009612 |
|  | 0032570 |
|  | 0009615 |
|  | 0006366 |
|  | 0007296 |
|  | 0005829 |
|  | 0043005 |
|  | 0005654 |
|  | 0005634 |
|  | 0042734 |
|  | 0003677 |
|  | 0000977 |
|  | 0006355 |
|  | 0043231 |
|  | 0042493 |
|  | 0014070 |
|  | 0000982 |
|  | 0001077 |
|  | 0043565 |

---

|  |  |
| --- | --- |
| KEGG ID | hsa:8061 |
|  | mmu:14283 |
|  | rno:25445 |

---

|  |  |
| --- | --- |
| Organism | Homo sapiens {Organism urn:agi-taxid:9606} |
|  | Mus musculus {Organism urn:agi-taxid:10090} |
|  | Rattus norvegicus {Organism urn:agi-taxid:10116} |
|  | Homo sapiens |
|  | Mus musculus |
|  | Rattus norvegicus |

---

|  |  |
| --- | --- |
| Mouse chromosome position | 19 4.33 cM |

---

|  |  |
| --- | --- |
| OMIM ID | 136515 |

---

|  |  |
| --- | --- |
| Rat chromosome position | 1q43 |

---

|  |  |
| --- | --- |
| Hugo ID | 13718 |
|  | HGNC:13718 |

---

|  |  |
| --- | --- |
| Human chromosome position | 11q13.1 |
|  | 11q13 |

---

|  |  |
| --- | --- |
| Swiss-Prot Accession | E9PPX2 |
|  | P15407 |
|  | E9PKL5 |
|  | A0A0S2Z595 |
|  | P15407.1 |
|  | P48755 |
|  | Q3UMK5 |
|  | P48755.2 |
|  | P10158 |
|  | P10158.1 |
|  | B4DR11 |
|  | Q6FG51 |
|  | O35285 |
|  | Q4V8K7 |

---

|  |  |
| --- | --- |
| PIR ID | S15750 |
|  | A27722 |

---

|  |  |
| --- | --- |
| GenBank ID | NC\_000011 |
|  | NR\_125339 |
|  | NM\_001300857 |
|  | NP\_001287786 |
|  | NM\_001300856 |
|  | NP\_001287785 |
|  | NM\_001300844 |
|  | NP\_001287773 |
|  | NM\_001300855 |
|  | NP\_001287784 |
|  | NM\_005438 |
|  | NP\_005429 |
|  | AJ297411 |
|  | CAC50237 |
|  | AJ297412 |
|  | AJ297413 |
|  | AP006287 |
|  | CH471076 |
|  | EAW74469 |
|  | D14493 |
|  | BAA03386 |
|  | D16365 |
|  | BAA03867 |
|  | KT583991 |
|  | AA120824 |
|  | AK222902 |
|  | BAD96622 |
|  | AK293297 |
|  | BAG56818 |
|  | AK299050 |
|  | BAG61123 |
|  | AK313718 |
|  | BAG36461 |
|  | BC016648 |
|  | AAH16648 |
|  | BM764259 |
|  | BM846271 |
|  | BP222547 |
|  | BQ937004 |
|  | CB123614 |
|  | CR542257 |
|  | CAG47053 |
|  | CR542278 |
|  | CAG47074 |
|  | KU178407 |
|  | ALQ33865 |
|  | KU178408 |
|  | ALQ33866 |
|  | S68214 |
|  | X16707 |
|  | CAA34679 |
|  | P15407 |
|  | NC\_000085 |
|  | NM\_010235 |
|  | NP\_034365 |
|  | AC122861 |
|  | AF017128 |
|  | AAB71369 |
|  | CH466612 |
|  | EDL33130 |
|  | AK144785 |
|  | BAE26065 |
|  | AK144839 |
|  | BAE26093 |
|  | AK145035 |
|  | BAE26198 |
|  | BC052917 |
|  | AAH52917 |
|  | U34245 |
|  | AAC52888 |
|  | P48755 |
|  | NC\_005100 |
|  | NM\_012953 |
|  | NP\_037085 |
|  | AC\_000069 |
|  | AAHX01009278 |
|  | AC109096 |
|  | CH473953 |
|  | EDM12489 |
|  | U24154 |
|  | AAA82045 |
|  | BC060582 |
|  | BC097342 |
|  | AAH97342 |
|  | M19651 |
|  | AAA41171 |
|  | P10158 |
|  | NC\_018922 |
|  | AMYH02024966 |
|  | DQ891054 |
|  | ABM81980 |
|  | DQ892703 |
|  | ABM83629 |
|  | DQ893332 |
|  | ABM84258 |
|  | DQ894233 |
|  | ABM85159 |
|  | DQ896648 |
|  | ABM87647 |
|  | EU176469 |
|  | ABW03920 |
|  | AC\_000041 |
|  | AAHY01141362 |
|  | XM\_005274311 |
|  | XP\_005274368 |
|  | AC\_000143 |
|  | ABBA01024097 |

---

|  |  |
| --- | --- |
| Swiss-Prot ID | FOSL1\_HUMAN |
|  | FOSL1\_MOUSE |
|  | FOSL1\_RAT |

---

|  |  |
| --- | --- |
| Cell Localization | Nucleus |

---

|  |  |
| --- | --- |
| Ensembl ID | ENSG00000175592 |
|  | ENSP00000436276.1 |
|  | ENST00000531493.5 |
|  | ENSP00000431594.1 |
|  | ENST00000532401.1 |
|  | ENSP00000393302.2 |
|  | ENST00000448083.6 |
|  | ENSP00000310170.2 |
|  | ENST00000312562.6 |
|  | ENSMUSG00000024912 |
|  | ENSMUSP00000025850.5 |
|  | ENSMUST00000025850.6 |
|  | ENSRNOG00000020552 |
|  | ENSRNOP00000027891.1 |
|  | ENSRNOT00000027891.2 |
|  | ENSP00000436276 |
|  | ENST00000531493 |
|  | ENSP00000431594 |
|  | ENST00000532401 |
|  | ENSP00000393302 |
|  | ENST00000448083 |
|  | ENSP00000310170 |
|  | ENST00000312562 |
|  | ENSMUSP00000025850 |
|  | ENSMUST00000025850 |
|  | ENSRNOP00000027891 |
|  | ENSRNOT00000027891 |

---

|  |  |
| --- | --- |
| MGI ID | MGI:107179 |

---

|  |  |
| --- | --- |
| RGD ID | 2627 |

---

|  |  |
| --- | --- |
| Unigene ID | Hs.283565 |
|  | Mm.6215 |
|  | Rn.11306 |

---

|  |  |
| --- | --- |
| Homologene ID | 3967 |

---

|  |  |
| --- | --- |
| Shape | O-vertex |

---
