# Supplementary material for: Analysis of PPARγ Signaling Activity in Psoriasis
Source: Int J Mol Sci. 2021 Aug 10;22(16):8603. doi: 10.3390/ijms22168603 (PMC8395241; doi:10.3390/ijms22168603)
Supplement: Supplementary file 1 [file ijms-22-08603-s001.zip › Supplemental materials_Analysis of PPARg signaling activity in psoriasis/Pathway models/Models images and html files/Anti-psoriatic drugs influence PPARG signaling/237427.html]

psoriasis


# Disease psoriasis

|  |  |
| --- | --- |
| URN | urn:agi-meshdis:Psoriasis |
| Total Entities | 8 |
| Connectivity | 4629 |
| Name | psoriasis |

---

|  |  |
| --- | --- |
| ChildConcepts | pustular psoriasis |
|  | scalp psoriasis |
|  | parapsoriasis |
|  | plaque psoriasis |
|  | guttate psoriasis |
|  | inverse psoriasis |
|  | erythrodermic psoriasis |
|  | nail psoriasis |

---

|  |  |
| --- | --- |
| Pathway | Proteins Involved in Psoriasis |
|  | Genes with Mutations Associated with Psoriasis |
|  | Proteins with Altered Expression in Psoriasis |
|  | Proteins with Altered Expression in Psoriatic Arthritis |
|  | New Pathway (1) |
|  | prarg negative regulators, ps-positive |
|  | pprarg neg, uknown targets, ps-positive |
|  | prarg neg,ukn expres targets, ps-positive |
|  | PPARG inhibits phenotypes assotiated with psoriasis |
|  | efects selected |
|  | Neighbors of psoriasis |
|  | comon ps\_pos, pprarg\_neg targets |
|  | New Pathway (5) |
|  | Overview. Diseases of the skin (chapter 11) |
|  | Anti-psoriatic drugs influence PPARG signaling |

---

|  |  |
| --- | --- |
| MedScan ID | 9010649 |

---

|  |  |
| --- | --- |
| Alias | psoriasiform rashes |
|  | psoriatic |
|  | psoriatic epidermis |
|  | psoriases |
|  | psoriasiform dermatoses |
|  | psoriasiform dermatosis |
|  | psoriasis unspecified |
|  | psoriasiform rash |
|  | psoriatic skin |
|  | psoriatic skins |
|  | willan lepra |
|  | psoriasiform dermatitis |
|  | psoriasis |
|  | psoriasiform lesion |
|  | psoriasiform lesions |

---

|  |  |
| --- | --- |
| MeSH ID | D011565 |
|  | M0017975 |

---

|  |  |
| --- | --- |
| MeSH Heading | Psoriasis |

---
