# Supplementary material for: Analysis of PPARγ Signaling Activity in Psoriasis
Source: Int J Mol Sci. 2021 Aug 10;22(16):8603. doi: 10.3390/ijms22168603 (PMC8395241; doi:10.3390/ijms22168603)
Supplement: Supplementary file 1 [file ijms-22-08603-s001.zip › Supplemental materials_Analysis of PPARg signaling activity in psoriasis/Pathway models/Models images and html files/Anti-psoriatic drugs influence PPARG signaling/268164.html]

pioglitazone


# Small Molecule pioglitazone

|  |  |
| --- | --- |
| URN | urn:agi-cas:105355-27-9 |
| Total Entities | 0 |
| Connectivity | 2934 |
| Name | pioglitazone |
| Molecular Weight | 392.898000 |
| XLogP | 2.341000 |

---

|  |  |
| --- | --- |
| Pathway | Anti-psoriatic drugs influence PPARG signaling |

---

|  |  |
| --- | --- |
| MedScan ID | 1043036 |

---

|  |  |
| --- | --- |
| Alias | pioglitazona |
|  | 105355-27-9 |
|  | Actos |
|  | Pioglitazone hydrochloride |
|  | U 72107A |
|  | 111025-46-8 |
|  | (+/-)-5-((4-(2-(5-ethyl-2-pyridinyl)ethoxy)phenyl)methyl)-2,4-Thiazolidinedione |
|  | 5-[[4-[2-[(5-ethyl-2-pyridyl)]ethoxy]phenyl]methyl]thiazolidine-2,4-dione |
|  | HSDB 7322 |
|  | U-72107 |
|  | [3H]pioglitazone |
|  | pioglitazone |
|  | 5-((4-(2-(5-ethyl-2-pyridinyl)ethoxy)phenyl)methyl)-2,4-Thiazolidinedione (+/-)-monohydrochloride |
|  | (+/-)-5-[p-[2-[(5-ethyl-2-pyridyl)ethoxy]benzyl]-2,4-thiazolidinedione |
|  | U72107 |
|  | (+/-) 5-[p-[2-[(5-ethyl-2-pyridyl)ethoxy]benzyl]-2,4-thiazolidinedione |
|  | 5-(4-(2-(5-ethyl-2-pyridyl)ethoxy)benzyl)-2,4-thiazolidinedione |
|  | AD 4833 |
|  | Pioglitazone HCl |
|  | pioglitazonum |
|  | 5-((4-(2-(5-Ethyl-2-pyridinyl)ethoxy)phenyl)methyl)-2,4-thiazolidinedione |
|  | 112529-15-4 |
|  | (+/-)-5-(p-(2-(5-Ethyl-2-pyridyl)ethoxy)benzyl)-2,4-thiazolidinedione monohydrochloride |

---

|  |  |
| --- | --- |
| CAS ID | 105355-27-9 |
|  | 112529-15-4 |
|  | 127676-30-6 |
|  | 111025-46-8 |
|  | 198077-89-3 |

---

|  |  |
| --- | --- |
| Reaxys ID | 11338157 |
|  | 27504417 |
|  | 3579575 |
|  | 3595485 |

---

|  |  |
| --- | --- |
| ChEBI ID | 8228 |

---

|  |  |
| --- | --- |
| PharmaPendium ID | Pioglitazone Hydrochloride |

---

|  |  |
| --- | --- |
| HMDB ID | HMDB15264 |

---

|  |  |
| --- | --- |
| KEGG ID | C07675 |

---

|  |  |
| --- | --- |
| InChIKey | HYAFETHFCAUJAY-UHFFFAOYSA-N |
|  | GHUUBYQTCDQWRA-UHFFFAOYSA-N |

---

|  |  |
| --- | --- |
| Molecular Formula | C19H20N2O3S |
|  | C19H21ClN2O3S |

---

|  |  |
| --- | --- |
| PubChem SID | 135017830 |
|  | 135035596 |

---

|  |  |
| --- | --- |
| PubChem CID | 60560 |
|  | 4829 |

---

|  |  |
| --- | --- |
| XLogP-AA | 3.8 |

---

|  |  |
| --- | --- |
| IUPAC Name | 5-[4-[2-(5-ethyl-2-pyridyl)ethoxy]benzyl]thiazolidine-2,4-quinone |
|  | 5-[4-[2-(5-ethyl-2-pyridyl)ethoxy]benzyl]thiazolidine-2,4-quinone;hydrochloride |

---

|  |  |
| --- | --- |
| Rotatable Bond Count | 7 |

---
