# Supplementary material for: Analysis of PPARγ Signaling Activity in Psoriasis
Source: Int J Mol Sci. 2021 Aug 10;22(16):8603. doi: 10.3390/ijms22168603 (PMC8395241; doi:10.3390/ijms22168603)
Supplement: Supplementary file 1 [file ijms-22-08603-s001.zip › Supplemental materials_Analysis of PPARg signaling activity in psoriasis/Pathway models/Models images and html files/Anti-psoriatic drugs influence PPARG signaling/28943.html]

FOXP3


# Protein FOXP3

|  |  |
| --- | --- |
| URN | urn:agi-llid:28953 |
| Total Entities | 0 |
| Connectivity | 3780 |
| Name | FOXP3 |
| Description | forkhead box P3 |
| Notes | The protein encoded by this gene is a member of the forkhead/winged-helix family of transcriptional regulators. Defects in this gene are the cause of immunodeficiency polyendocrinopathy, enteropathy, X-linked syndrome (IPEX), also known as X-linked autoimmunity-immunodeficiency syndrome. Alternatively spliced transcript variants encoding different isoforms have been identified. [provided by RefSeq, Jul 2008] |
| Primary Cell Localization | Nucleus |
| Class | Transcription factor |

---

|  |  |
| --- | --- |
| Pathway | T-Cell Maturation (Hypothesis) |
|  | Treg-Cell Differentiation |
|  | AHR Signaling in Treg and Dendritic Cells Function |
|  | Kynurenine/AHR Signaling in Treg Cell Activation |
|  | Proteins Involved in Atherosclerosis |
|  | Dendritic Cells Function in Atherosclerosis |
|  | Lymphocyte Mediated Myocardial Injury in Myocarditis |
|  | Proteins Involved in Melanoma |
|  | T-Cells Differentiation Block in Psoriasis |
|  | Proteins with Altered Expression in Psoriasis |
|  | Treg-Cell Function in Diabetes Mellitus Type 1 |
|  | non-Suppressive Treg-Cell in Diabetes Mellitus Type 1 |
|  | TLR2 Signaling in Treg-Cell in Type 1 Diabetes (Animal Model) |
|  | Peripheral Tolerance to Autoantigens Recession in Diabetes Mellitus Type 1 |
|  | Proteins Involved in Diabetes Mellitus Type 1 |
|  | Treg-Cell Activation in Diabetes Mellitus |
|  | Neonatal Diabetes Mellitus |
|  | Proteins Involved in Celiac Disease |
|  | Th17-Cell Activation in Crohn's Disease |
|  | Proteins Involved in Inflammatory Bowel Diseases |
|  | Autoimmune Polyglandular Syndromes Progression (Hypothesis) |
|  | Proteins Involved in Breast Cancer Related to ERBB2/VEGFR/Akt Signaling Pathway |
|  | Proteins Involved in Endometriosis |
|  | Proteins Involved in Chronic Obstructive Pulmonary Disease |
|  | CD80 -> AP-1 Expression Targets |
|  | CD81 Expression Targets |
|  | Elevated Receptors -> Expression Targets in Colon |
|  | Elevated Receptors -> Expression Targets in Appendix |
|  | STAT3 Facilitates the Function of Treg Cells and Cancer Progression |
|  | Treg Cells Promote Immunosuppression in Cancer Immune Escape |
|  | Effector T-cell Inactivation in Cancer Immune Escape |
|  | Adenosine/cAMP Promote Immunosuppression by Treg Cells |
|  | IL15 Expression Targets |
|  | IL6 Expression Targets |
|  | IL4 Expression Targets |
|  | IL2 Expression Targets |
|  | LDLR -> Expression Targets in Lymphoid System and Blood |
|  | Immunoglobulin Superfamily -> Expression Targets in Lymphoid System and Blood |
|  | NOTCH -> Expression Targets in Thymus |
|  | Elevated Receptors -> Expression Targets in Thymus |
|  | PDCD1 -> STAT Expression Targets |
|  | PDCD1 -> NFATC Expression Targets |
|  | PDCD1 -> AP-1 Expression Targets |
|  | TCR -> NFAT Expression Targets |
|  | TCR -> STAT Expression Targets |
|  | TGFB1-TGFBR1 Expression Targets |
|  | TGFB1-ACVRL1 Expression Targets |
|  | PPAR Psoriasis |
|  | pprarg neg, uknown targets, ps-positive |
|  | Model of PPARG signaling in psoriasis |
|  | PPARG negative regulators and targets |
|  | Model of PPARG related pathways in psoriasis (short version) |
|  | Figure 3\_Mixed network of dexamethasone targets |
|  | Figure 3\_Mixed network of dexamethasone targets |
|  | 1\_Differentiation of psoriatic T cells |
|  | 4\_1\_Polymorphisms associated with inflammatory bowel diseases |
|  | 2\_Defective tolerance of autoreactive T-cell in T1D |
|  | Model of PPARG signaling in psoriais (tested) |
|  | before laser treatment |
|  | Differentiation of psoriatic T cells |
|  | Anti-psoriatic drugs influence PPARG signaling |
|  | PPARG signaling after laser treatment |

---

|  |  |
| --- | --- |
| Group | Genes with Mutations Associated with Diabetes Mellitus Type 1 |
|  | Genes with Mutations Associated with Neonatal Diabetes Mellitus |

---

|  |  |
| --- | --- |
| MedScan ID | 28953 |

---

|  |  |
| --- | --- |
| LocusLink ID | 28953 |
|  | 50943 |
|  | 20371 |
|  | 317382 |

---

|  |  |
| --- | --- |
| Alias | RP23-54C14.1 |
|  | JM2 protein |
|  | OTTHUMP00000025832 |
|  | XPID |
|  | FOXP3 transcription factor |
|  | RGD1562112 |
|  | FOXP3delta7 |
|  | scurfy |
|  | MGC141963 |
|  | MGC141961 |
|  | OTTHUMP00000025833 |
|  | FOXP3 |
|  | immunodeficiency, polyendocrinopathy, enteropathy, X-linked locus |
|  | Zinc finger protein JM2 |
|  | PIDX |
|  | OTTMUSP00000017962 |
|  | OTTMUSP00000017961 |
|  | Scurfins |
|  | transcription factor FOXP3 |
|  | forkhead box P3, scurfy |
|  | OTTMUSP00000017952 |
|  | AIID |
|  | forkhead box P3 |
|  | X-linked immunodeficiency, polyendocrinopathy, enteropathy gene |
|  | Scurfin |
|  | X-linked immune dysregulation, polyendocrinopathy, enteropathy gene |
|  | DIETER |
|  | JM2 |
|  | IPEX |
|  | immune dysregulation, polyendocrinopathy, enteropathy, X-linked |
|  | immunodeficiency, polyendocrinopathy, enteropathy, X-linked |
|  | forkhead box protein P3 |
|  | sf |

---

|  |  |
| --- | --- |
| GO ID | 0003677 |
|  | 0003700 |
|  | 0000981 |
|  | 0051059 |
|  | 0051525 |
|  | 0000978 |
|  | 0035035 |
|  | 0042826 |
|  | 0046872 |
|  | 0042803 |
|  | 0043565 |
|  | 0003714 |
|  | 0001782 |
|  | 0002362 |
|  | 0042110 |
|  | 0043029 |
|  | 0002456 |
|  | 0050852 |
|  | 0006338 |
|  | 0001816 |
|  | 0002262 |
|  | 0032792 |
|  | 0043433 |
|  | 0032088 |
|  | 0002725 |
|  | 0042130 |
|  | 2000320 |
|  | 0046007 |
|  | 0008285 |
|  | 0002677 |
|  | 0042036 |
|  | 0050710 |
|  | 0035067 |
|  | 0031064 |
|  | 0050777 |
|  | 0045077 |
|  | 0032689 |
|  | 0032693 |
|  | 0032700 |
|  | 0045085 |
|  | 0032703 |
|  | 0032713 |
|  | 0032714 |
|  | 0032715 |
|  | 0048294 |
|  | 0000122 |
|  | 0045892 |
|  | 0032720 |
|  | 0032831 |
|  | 0002669 |
|  | 0035066 |
|  | 0033092 |
|  | 0032753 |
|  | 0002851 |
|  | 0045944 |
|  | 0045893 |
|  | 0032914 |
|  | 0002667 |
|  | 0030111 |
|  | 0048302 |
|  | 0045589 |
|  | 0006355 |
|  | 0009615 |
|  | 0002513 |
|  | 0005737 |
|  | 0005654 |
|  | 0005634 |
|  | 0032991 |
|  | 0003676 |
|  | 0002361 |
|  | 0010629 |
|  | 0050728 |
|  | 0050672 |
|  | 0002666 |
|  | 0010628 |
|  | 0045591 |
|  | 0002637 |
|  | 0006357 |
|  | 0002507 |
|  | 0045066 |
|  | 0005622 |
|  | 0006351 |
|  | 0003705 |
|  | 0001047 |
|  | 0043234 |
|  | 0009653 |
|  | 0030154 |
|  | 0006366 |
|  | 0008301 |
|  | 0003682 |
|  | 0003690 |
|  | 0046982 |
|  | 0008270 |
|  | 0043010 |
|  | 0021757 |
|  | 0021549 |
|  | 0021987 |
|  | 0009790 |
|  | 0040007 |
|  | 0048286 |
|  | 0007389 |
|  | 0060501 |
|  | 0002053 |
|  | 0009791 |
|  | 0021758 |
|  | 0060013 |
|  | 0007519 |
|  | 0048745 |
|  | 0042297 |
|  | 0005667 |

---

|  |  |
| --- | --- |
| KEGG ID | hsa:50943 |
|  | mmu:20371 |
|  | rno:317382 |

---

|  |  |
| --- | --- |
| Organism | Homo sapiens {Organism urn:agi-taxid:9606} |
|  | Mus musculus {Organism urn:agi-taxid:10090} |
|  | Rattus norvegicus {Organism urn:agi-taxid:10116} |
|  | Homo sapiens |
|  | Mus musculus |
|  | Rattus norvegicus |

---

|  |  |
| --- | --- |
| Mouse chromosome position | X 3.41 cM |
|  | X 2.1 cM |

---

|  |  |
| --- | --- |
| OMIM ID | 300292 |
|  | 304790 |
|  | 222100 |

---

|  |  |
| --- | --- |
| Rat chromosome position | Xq12 |
|  | Xq13 |

---

|  |  |
| --- | --- |
| Hugo ID | 6106 |
|  | HGNC:6106 |

---

|  |  |
| --- | --- |
| Human chromosome position | Xp11.23 |

---

|  |  |
| --- | --- |
| Swiss-Prot Accession | Q9BZS1 |
|  | B7ZLG1 |
|  | Q9BZS1.1 |
|  | Q53Z59 |
|  | Q99JB6 |
|  | Q99JB6.1 |
|  | D3ZKI1 |
|  | D4Q8I2 |
|  | A5HJT1 |
|  | B7ZLG0 |
|  | B9UN80 |
|  | O60827 |
|  | Q14DD8 |
|  | Q4ZH51 |

---

|  |  |
| --- | --- |
| GenBank ID | NC\_000023 |
|  | XM\_006724533 |
|  | XP\_006724596 |
|  | NM\_014009 |
|  | NP\_054728 |
|  | XM\_017029567 |
|  | XP\_016885056 |
|  | NM\_001114377 |
|  | NP\_001107849 |
|  | NG\_007392 |
|  | AC232271 |
|  | AF235097 |
|  | CH471224 |
|  | EAW50671 |
|  | EAW50672 |
|  | EAW50673 |
|  | GN344065 |
|  | CAY55968 |
|  | JA738833 |
|  | CCF77062 |
|  | JB259913 |
|  | CDH61704 |
|  | KP784358 |
|  | KP784359 |
|  | KP784360 |
|  | KP784361 |
|  | KP784362 |
|  | KP784363 |
|  | KP784364 |
|  | KP784365 |
|  | KP784366 |
|  | KP784367 |
|  | KP784368 |
|  | KP784369 |
|  | AF277993 |
|  | AAG53607 |
|  | AJ005891 |
|  | CAA06748 |
|  | AK292052 |
|  | BAF84741 |
|  | AK299988 |
|  | BAG61809 |
|  | BC113401 |
|  | AAI13402 |
|  | BC113403 |
|  | AAI13404 |
|  | BC143785 |
|  | AAI43786 |
|  | BC143786 |
|  | AAI43787 |
|  | BQ184335 |
|  | DB342786 |
|  | DQ010327 |
|  | AAY27088 |
|  | EF534714 |
|  | ABQ15210 |
|  | EU855812 |
|  | ACJ46653 |
|  | Q9BZS1 |
|  | NC\_000086 |
|  | NM\_001199347 |
|  | NP\_001186276 |
|  | NM\_054039 |
|  | NP\_473380 |
|  | NM\_001199348 |
|  | NP\_001186277 |
|  | AF277994 |
|  | AAG53608 |
|  | AL672231 |
|  | AL731793 |
|  | CH466638 |
|  | EDL33899 |
|  | EDL33900 |
|  | AB673115 |
|  | AF277991 |
|  | AAG53605 |
|  | AF277992 |
|  | AAG53606 |
|  | AK157447 |
|  | BAE34089 |
|  | AK210240 |
|  | AK212556 |
|  | AK216372 |
|  | AY357712 |
|  | AAR11305 |
|  | AY357713 |
|  | AAR11306 |
|  | BC132333 |
|  | AAI32334 |
|  | BC132335 |
|  | AAI32336 |
|  | DQ387959 |
|  | ABD52722 |
|  | KF471319 |
|  | AHA56672 |
|  | Q99JB6 |
|  | NC\_005120 |
|  | NM\_001108250 |
|  | NP\_001101720 |
|  | XM\_006256731 |
|  | XP\_006256793 |
|  | AC\_000089 |
|  | AABR07073520 |
|  | AAHX01109184 |
|  | CH474078 |
|  | EDL83847 |
|  | EDL83848 |
|  | AB232988 |
|  | BAJ05810 |
|  | AB232989 |
|  | BAJ05811 |
|  | AB232990 |
|  | BAJ05812 |
|  | XM\_011543915 |
|  | XP\_011542217 |
|  | XM\_011543918 |
|  | XP\_011542220 |
|  | XM\_011543919 |
|  | XP\_011542221 |
|  | XM\_011543916 |
|  | XP\_011542218 |
|  | XM\_011543917 |
|  | XP\_011542219 |
|  | NC\_018934 |
|  | AMYH02039324 |
|  | BC111853 |
|  | AAI11854 |
|  | HQ258232 |
|  | ADR82986 |
|  | AC\_000042 |
|  | AAHY01206809 |
|  | XM\_005272611 |
|  | XP\_005272668 |
|  | XM\_005272610 |
|  | XP\_005272667 |
|  | AC\_000155 |
|  | ABBA01046142 |
|  | ABBA01046143 |
|  | NW\_004070880 |
|  | CAM25950 |

---

|  |  |
| --- | --- |
| Swiss-Prot ID | FOXP3\_HUMAN |
|  | FOXP3\_MOUSE |

---

|  |  |
| --- | --- |
| Cell Localization | Nucleus |
|  | Cytoplasm |

---

|  |  |
| --- | --- |
| Ensembl ID | ENSG00000049768 |
|  | ENSP00000365372.2 |
|  | ENST00000376199.7 |
|  | ENSP00000365380.4 |
|  | ENST00000376207.9 |
|  | ENSP00000396415.3 |
|  | ENST00000455775.7 |
|  | ENSP00000451208.1 |
|  | ENST00000557224.6 |
|  | ENSMUSG00000039521 |
|  | ENSMUSP00000111405.1 |
|  | ENSMUST00000115740.8 |
|  | ENSMUSP00000111403.1 |
|  | ENSMUST00000115738.7 |
|  | ENSMUSP00000111404.1 |
|  | ENSMUST00000115739.8 |
|  | ENSRNOG00000011702 |
|  | ENSRNOP00000015641.4 |
|  | ENSRNOT00000015641.6 |
|  | ENSRNOP00000073801.1 |
|  | ENSRNOT00000077391.1 |
|  | ENST00000376199.6 |
|  | ENST00000557224.5 |
|  | ENSMUST00000115739.7 |
|  | ENST00000455775.6 |
|  | ENST00000376207.8 |
|  | ENSMUST00000115740.7 |
|  | ENSP00000365372 |
|  | ENST00000376199 |
|  | ENSP00000365380 |
|  | ENST00000376207 |
|  | ENSP00000396415 |
|  | ENST00000455775 |
|  | ENSMUSP00000111405 |
|  | ENSMUST00000115740 |
|  | ENSMUSP00000111403 |
|  | ENSMUST00000115738 |
|  | ENSMUSP00000111404 |
|  | ENSMUST00000115739 |
|  | ENSRNOP00000015641 |
|  | ENSRNOT00000015641 |
|  | ENSRNOP00000073801 |
|  | ENSRNOT00000077391 |

---

|  |  |
| --- | --- |
| MGI ID | MGI:1891436 |
|  | 1891436 |

---

|  |  |
| --- | --- |
| RGD ID | 1562112 |

---

|  |  |
| --- | --- |
| Unigene ID | Mm.182291 |
|  | Mm.288192 |
|  | Rn.177272 |
|  | Hs.247700 |

---

|  |  |
| --- | --- |
| Homologene ID | 8516 |

---

|  |  |
| --- | --- |
| Shape | O-vertex |

---

|  |  |
| --- | --- |
| IPI ID | IPI00115123 |
|  | IPI00328094 |
|  | IPI00604500 |
|  | IPI00644203 |

---
