# Supplementary material for: Analysis of PPARγ Signaling Activity in Psoriasis
Source: Int J Mol Sci. 2021 Aug 10;22(16):8603. doi: 10.3390/ijms22168603 (PMC8395241; doi:10.3390/ijms22168603)
Supplement: Supplementary file 1 [file ijms-22-08603-s001.zip › Supplemental materials_Analysis of PPARg signaling activity in psoriasis/Pathway models/Models images and html files/Anti-psoriatic drugs influence PPARG signaling/29564.html]

paclitaxel


# Small Molecule paclitaxel

|  |  |
| --- | --- |
| URN | urn:agi-cas:33069-62-4 |
| Total Entities | 12 |
| Connectivity | 4233 |
| Name | paclitaxel |
| Molecular Weight | 853.918000 |
| XLogP | 2.500000 |

---

|  |  |
| --- | --- |
| ChildConcepts | 3'-p-hydroxypaclitaxel |
|  | 6 alpha-hydroxytaxol |
|  | DHA-paclitaxel |
|  | TOCOSOL paclitaxel |
|  | paclitaxel derivative |
|  | 7-acetyltaxol |
|  | 10-deacetyltaxol |
|  | 6,3'-p-dihydroxypaclitaxel |
|  | paclitaxel dihydrate |
|  | 7-epi-paclitaxel |

---

|  |  |
| --- | --- |
| Pathway | Endometrial Cancer |
|  | Prostate Cancer |
|  | Anti-psoriatic drugs influence PPARG signaling |

---

|  |  |
| --- | --- |
| MedScan ID | 1198793 |

---

|  |  |
| --- | --- |
| Alias | britaxol |
|  | coroxane |
|  | HSDB 6839 |
|  | taxanes paclitaxel |
|  | Anzatax |
|  | BMY 45622 |
|  | 7,11-Methano-5H-cyclodeca(3,4)benz(1,2-b)oxete, benzenepropanoic acid deriv. |
|  | NSC-125973 |
|  | (4 alpha)-Isomer of Paclitaxel |
|  | albumin-bound paclitaxel |
|  | padexol |
|  | Plaxicel |
|  | Paxceed |
|  | oaspac100 |
|  | s. dort paclitaxel |
|  | benzenepropanoic acid deriv. 7,11-Methano-5H-cyclodeca(3,4)benz(1,2-b)oxete |
|  | sb 05 (terpenoid) |
|  | EndoTAG-1 |
|  | bolus paclitaxel |
|  | bms181339 |
|  | mitotax |
|  | nanoparticle albumin bound paclitaxel |
|  | ifaxol |
|  | Capxol |
|  | ABI-007 |
|  | Paxene |
|  | taxus (drug) |
|  | formoxol |
|  | genexol pm |
|  | parexel |
|  | taxol paclitaxel |
|  | Onxol |
|  | TaxAlbin |
|  | infinnium |
|  | Xorane |
|  | biotax |
|  | Semi-Synthetic Paclitaxel |
|  | DRG-0190 |
|  | 12alpha,12balpha]]-beta-(benzoylamino)-alpha-hydroxybenzene propanoic acid |
|  | taycovit |
|  | MBT 0206 |
|  | [(3)H]-paclitaxel |
|  | intaxel |
|  | OncoGel |
|  | NSC-673089 |
|  | [3H]-Taxol |
|  | taxocris |
|  | asotax |
|  | Tax-11-en-9-one, 5beta,20-epoxy-1,2alpha,4,7beta,10beta,13alpha- hexahydroxy-, 4,10-diacetate 2-benzoate, 13-ester with (2R,3S)-N-benzoyl-3-phenylisoserine |
|  | QW 8184 |
|  | taxus express |
|  | paclitaxel nab |
|  | taxane paclitaxel |
|  | Baccatin III N-benzyl-beta-phenylisoserine Ester |
|  | Taxol A |
|  | PG-TXL |
|  | nab-paclitaxel |
|  | peclitaxel |
|  | NK-105 |
|  | Ebetaxel |
|  | 5beta,20-epoxy-1,2 alpha, 4,7beta, 10beta, 13alpha-hexahydroxy tax-11-en-9-one 4,10-diacetate 2-benzoate 13-ester with (2r, 3s)-n-benzoyl-3-phenylisoserine |
|  | LipoPac |
|  | pacitaxel |
|  | PROTAX |
|  | pazenir |
|  | OAS-PAC-100 |
|  | Paclitaxel, (4 alpha)-Isomer |
|  | Praxel |
|  | Yewtaxan |
|  | genexol |
|  | Paclitaxel |
|  | paxus |
|  | Abraxane |
|  | Paclitaxel, Semi-Synthetic |
|  | apealea |
|  | medixel |
|  | [(14)C]paclitaxel |
|  | paclitaxel, s. dort |
|  | bristaxol |
|  | 33069-62-4 |
|  | hunxol |
|  | polyglutamate paclitaxel |
|  | DTS-301 |
|  | 12alpha,12balpha]]-beta-(benzoylamino)-alpha-hydroxybenzene propanoate |
|  | pacxel |
|  | paclitaxel taxol |
|  | 5beta,20-Epoxy-1,2-alpha,4,7beta,10beta,13alpha-hexahydroxytax-11-en-9-one 4,10-diacetate 2-benzoate 13-ester with (2R,3S)-N-benzoyl-3-phenylisoserine |
|  | taxol |
|  | BMS 181339-01 |

---

|  |  |
| --- | --- |
| CAS ID | 33069-62-4 |
|  | 1203669-79-7 |
|  | 157069-30-2 |

---

|  |  |
| --- | --- |
| Reaxys ID | 12485534 |
|  | 1420456 |
|  | 1420457 |
|  | 23443221 |
|  | 4290260 |
|  | 4290261 |
|  | 4837659 |
|  | 4903097 |
|  | 5712582 |
|  | 8468496 |

---

|  |  |
| --- | --- |
| ChEBI ID | 45863 |

---

|  |  |
| --- | --- |
| PharmaPendium ID | Paclitaxel |

---

|  |  |
| --- | --- |
| HMDB ID | HMDB15360 |

---

|  |  |
| --- | --- |
| KEGG ID | C07394 |

---

|  |  |
| --- | --- |
| InChIKey | RCINICONZNJXQF-MZXODVADSA-N |

---

|  |  |
| --- | --- |
| Molecular Formula | C47H51NO14 |

---

|  |  |
| --- | --- |
| PubChem SID | 134998291 |

---

|  |  |
| --- | --- |
| PubChem CID | 36314 |

---

|  |  |
| --- | --- |
| Rotatable Bond Count | 14 |

---
