# Supplementary material for: Analysis of PPARγ Signaling Activity in Psoriasis
Source: Int J Mol Sci. 2021 Aug 10;22(16):8603. doi: 10.3390/ijms22168603 (PMC8395241; doi:10.3390/ijms22168603)
Supplement: Supplementary file 1 [file ijms-22-08603-s001.zip › Supplemental materials_Analysis of PPARg signaling activity in psoriasis/Pathway models/Models images and html files/Anti-psoriatic drugs influence PPARG signaling/38571.html]

calcitriol


# Small Molecule calcitriol

|  |  |
| --- | --- |
| URN | urn:agi-cas:32222-06-3 |
| Total Entities | 1 |
| Connectivity | 5118 |
| Name | calcitriol |
| Class | Endogenous compound |
| Molecular Weight | 416.646000 |
| XLogP | 6.745000 |

---

|  |  |
| --- | --- |
| ChildConcepts | 5,6-trans-25-hydroxycholecalciferol |

---

|  |  |
| --- | --- |
| Pathway | Ca2+ Reabsorption Decline in Intestine |
|  | Osteoblast Function Decline in Gout |
|  | PAPA Syndrome |
|  | Blau Syndrome |
|  | Ca2+ Reabsorption Decline in Kidney |
|  | Hyperparathyroidism, Secondary Effect |
|  | Ca2+ Absorption Decline in Intestine in Osteoporosis |
|  | Prostate Cancer |
|  | Ions Reabsorption Dysregulation in Nephrolithiasis |
|  | Ca2+ Reabsorption Dysregulation in Urolithiasis |
|  | Biosynthesis of Cholesterol |
|  | Anti-psoriatic drugs influence PPARG signaling |

---

|  |  |
| --- | --- |
| MedScan ID | 1198576 |

---

|  |  |
| --- | --- |
| Alias | 1,25(OH)2 vitamin D |
|  | 20-epi-1alpha,25-dihydroxycholecaliferol |
|  | 32222-06-3 |
|  | roical |
|  | citrihexal |
|  | bonky |
|  | 1-alpha,25-Dihydroxyvitamin D3 |
|  | 1alpha,25(OH)2D3 |
|  | Renatriol |
|  | toptriol |
|  | kosteo |
|  | DM200 |
|  | 1-hydroxylated 25-hydroxyvitamin-D3 |
|  | 66772-14-3 |
|  | 1,25 dihydroxy cholecalciferol |
|  | hitrol |
|  | (3H)1,25(OH)2D3 |
|  | Vitamin D3 derivative 1,25-dihydroxyvitamin D3 |
|  | 1,25-dihydroxyvitamin D3 |
|  | (1alpha,3beta,5Z,7E)-9,10-Secocholesta-5,7,10(19)-triene-1,3,25-triol |
|  | Bocatriol |
|  | Sitriol |
|  | lemytriol |
|  | alpha 25-dihydroxyvitamin D3 |
|  | ecatrol f |
|  | vitamin D 1,25(OH)2D3 |
|  | Calcitriolum |
|  | kolkatriol |
|  | 1a,25-dihydroxycholecalciferol |
|  | 9,10 secocholesta 5,7,10(19) triene 1alpha,3beta,25 triol |
|  | 1,25 dihydrocholecalciferol |
|  | 1alpha,25-Dihydroxyvitamin D |
|  | 1-alfa,25-dihydroxycholecalciferol |
|  | 1-alpha,-1,25-Dihydroxyvitamin D3 |
|  | rolsical |
|  | Osteotriol |
|  | Soltriol |
|  | 1alpha,25 dihydroxycalciferol |
|  | 1,25-dihydroxycalciferol |
|  | cicarol |
|  | (5Z,7E)-9,10-Secocholesta-5,7,10(19)-triene-1-alpha,3-beta,25-triol |
|  | alpha 25-dihydroxycholecalciferol |
|  | rexamat |
|  | 1,25(OH)(2)-Vitamin D(3) |
|  | Calcijex |
|  | 1 alpha,25-dihydroxycholecalciferol |
|  | 1,25-DHCC |
|  | 1-alpha-25-DihydroxyvitaminD3 |
|  | vectical |
|  | DN-101 |
|  | 1alpha,25(OH)(2)-vitamin D(3) |
|  | 1,25D |
|  | Difix |
|  | ecatrol |
|  | CCRIS 5522 |
|  | 25 dihydroxycholecalciferol |
|  | 1,25(OH)2D |
|  | Calcitriol KyraMed |
|  | Decostriol |
|  | HSDB 3482 |
|  | cabone |
|  | 25-dihydroxyvitamin D |
|  | 1(gr a),25-dihydroxyvitamin D3 |
|  | EINECS 250-963-8 |
|  | poscal |
|  | RO 215535 |
|  | U 49562 |
|  | 1alpha 25-Dihydroxy-Vitamin D-3 |
|  | 1(gr a),25-dihydroxycholecalciferol |
|  | 1a,25-(OH)2D3 |
|  | meditrol |
|  | RO 21-5535 |
|  | 1,25-Dihydroxycholecaliferol |
|  | 1,25D3 |
|  | Calcitriol |
|  | 125338-24-1 |
|  | [3H]1,25-dihydroxycholecalciferol |
|  | colecalciferol 1,25 diol |
|  | [3H]1,25(OH)2D3 |
|  | 1,25(OH2)D3 |
|  | Topitriol |
|  | 25-dihydroxyvitamin |
|  | 1,25-Dihydroxyvitamin D |
|  | tariol |
|  | Rocaltrol |
|  | 25[OH]2 vitamin D3 |
|  | 9,10-Seco(5Z,7E)-5,7,10(19)-cholestatriene-1alpha,3beta,25-triol |
|  | Calcitriol-Nefro |
|  | 1,25-dihydroxyvitamin |
|  | 1,25-dihydroxyvitamin D3 (calcitriol) |
|  | Tirocal |
|  | 1-25 vitamin D3 |
|  | caraben sc |
|  | 1,25 dihydroxycolecalciferol |
|  | [3H]calcitriol |
|  | 1,25-dihydroxy vitamin D3 |
|  | 1,25-dihydroxycholecalciferol |
|  | Silkis |
|  | 1 alpha,25-dihydroxycolecalciferol |
|  | triocalcit |

---

|  |  |
| --- | --- |
| CAS ID | 32222-06-3 |
|  | 1000873-74-4 |
|  | 125338-24-1 |
|  | 69878-52-0 |

---

|  |  |
| --- | --- |
| Reaxys ID | 11327511 |
|  | 2065596 |
|  | 2065597 |
|  | 2065598 |
|  | 2065599 |
|  | 2227646 |
|  | 2227647 |
|  | 2341008 |
|  | 2341009 |
|  | 2512362 |
|  | 2512363 |
|  | 2512364 |
|  | 3656842 |
|  | 4718471 |
|  | 5307209 |
|  | 5485108 |
|  | 5771818 |
|  | 5771819 |
|  | 5771820 |

---

|  |  |
| --- | --- |
| ChEBI ID | 17823 |

---

|  |  |
| --- | --- |
| PharmaPendium ID | Calcitriol |

---

|  |  |
| --- | --- |
| HMDB ID | HMDB01903 |

---

|  |  |
| --- | --- |
| KEGG ID | C01673 |

---

|  |  |
| --- | --- |
| InChIKey | GMRQFYUYWCNGIN-NKMMMXOESA-N |

---

|  |  |
| --- | --- |
| Molecular Formula | C27H44O3 |

---

|  |  |
| --- | --- |
| PubChem SID | 134999639 |

---

|  |  |
| --- | --- |
| PubChem CID | 5280453 |

---

|  |  |
| --- | --- |
| XLogP-AA | 5.1 |

---

|  |  |
| --- | --- |
| IUPAC Name | (1R,3S,5Z)-5-[(2E)-2-[(1R,3aS,7aR)-1-[(1R)-5-hydroxy-1,5-dimethyl-hexyl]-7a-methyl-2,3,3a,5,6,7-hexahydro-1H-inden-4-ylidene]ethylidene]-4-methylene-cyclohexane-1,3-diol |

---

|  |  |
| --- | --- |
| Rotatable Bond Count | 6 |

---
