# Supplementary material for: Analysis of PPARγ Signaling Activity in Psoriasis
Source: Int J Mol Sci. 2021 Aug 10;22(16):8603. doi: 10.3390/ijms22168603 (PMC8395241; doi:10.3390/ijms22168603)
Supplement: Supplementary file 1 [file ijms-22-08603-s001.zip › Supplemental materials_Analysis of PPARg signaling activity in psoriasis/Pathway models/Models images and html files/Anti-psoriatic drugs influence PPARG signaling/40091.html]

steroid


# Small Molecule steroid

|  |  |
| --- | --- |
| URN | urn:agi-smol:steroids |
| Total Entities | 36 |
| Connectivity | 9889 |
| Name | steroid |
| Class | Endogenous compound |

---

|  |  |
| --- | --- |
| ChildConcepts | spirostan derivative |
|  | sterols |
|  | 17-ketosteroid |
|  | ganaxolone |
|  | androstane derivative |
|  | pamaqueside |
|  | pregnane derivative |
|  | diosgenin |
|  | chandonium iodide |
|  | sarsasapogenin |
|  | smilagenin |
|  | hecogenin |
|  | 1,4,6-androstatriene-3,17-dione |
|  | alisol A |
|  | pregnane |
|  | 19-norsteroid |
|  | PX-866 |
|  | 5alpha-androstane derivative |
|  | 5 beta-androstane derivative |
|  | 8-azasteroid |
|  | azasteroid |
|  | catatoxic steroid |
|  | steroid hormone |
|  | estrane derivative |
|  | gonane derivative |
|  | hydroxysteroid |
|  | N,N-Diethyl-4-methyl-3-oxo-4-aza-5 alpha-androst-1-ene-17 beta-carboxamide |
|  | oxosteroid |
|  | secosteroid |
|  | fluasterone |
|  | clascoterone |
|  | rostafuroxin |
|  | androstenol |
|  | zuranolone |
|  | etiocholanolone |
|  | macrostemonoside A |

---

|  |  |
| --- | --- |
| Pathway | Steroids Induced Cataract |
|  | Metabolism of Glycerophospholipids and Ether Lipids |
|  | Anti-psoriatic drugs influence PPARG signaling |

---

|  |  |
| --- | --- |
| MedScan ID | 1808985 |

---

|  |  |
| --- | --- |
| Alias | steroid derivative |
|  | steroid-14C |
|  | steroid compound |
|  | steroid |
|  | steroid derivatives |
|  | cyclosteroids |
|  | [14C] steroids |
|  | steroids |
|  | steroid-3H |
|  | steroidal compound |
|  | [3H]-steroids |

---
