# Supplementary material for: Analysis of PPARγ Signaling Activity in Psoriasis
Source: Int J Mol Sci. 2021 Aug 10;22(16):8603. doi: 10.3390/ijms22168603 (PMC8395241; doi:10.3390/ijms22168603)
Supplement: Supplementary file 1 [file ijms-22-08603-s001.zip › Supplemental materials_Analysis of PPARg signaling activity in psoriasis/Pathway models/Models images and html files/Anti-psoriatic drugs influence PPARG signaling/46810.html]

methotrexate


# Small Molecule methotrexate

|  |  |
| --- | --- |
| URN | urn:agi-cas:59-05-2 |
| Total Entities | 16 |
| Connectivity | 3913 |
| Name | methotrexate |
| Molecular Weight | 476.429000 |
| XLogP | -1.800000 |
| ObjectType | Small Molecule |

---

|  |  |
| --- | --- |
| ChildConcepts | folate |
|  | dichloromethotrexate |
|  | D-amethopterin |
|  | 9-methyl pteroylglutamic acid |
|  | methopterin |
|  | 3',5'-difluoromethotrexate |
|  | 4-aminopteroylaspartic acid |
|  | isoaminopterin |
|  | gamma-tert-butyl methotrexate |
|  | 7-hydroxymethotrexate |
|  | tetrahydroamethopterin |
|  | gamma-tBAMT |
|  | gamma-fluoromethotrexate |
|  | 2-desamino-2-methylaminopterin |
|  | folate polyglutamate |
|  | aminopterin |

---

|  |  |
| --- | --- |
| Pathway | Acute Myeloid Leukemia |
|  | B-Cell Acute Lymphoblastic Leukemia |
|  | T-Cell Acute Lymphoblastic Leukemia |
|  | Anti-psoriatic drugs influence PPARG signaling |

---

|  |  |
| --- | --- |
| MedScan ID | 1209078 |

---

|  |  |
| --- | --- |
| Alias | Methylaminopterinum |
|  | DL-N-(p-(((2,4-diamino-6-pteridinyl)methyl)methylamino)benzoyl)-Glutamic acid |
|  | L-glutamyl 3-4-3H |
|  | methohexate |
|  | DRG-0024 |
|  | methyl aminopterin |
|  | R 9985 |
|  | N-(p-(((2,4-Diamino-6-pteridinyl)methyl)methylamino)benzoyl)-L-(+)-glutamate |
|  | ametopterine |
|  | [3',5',9-3H]methotrexate |
|  | Glutamic acid, N-(p-(((2,4-diamino-6-pteridinyl)methyl)methylamino)benzoyl)-, sodium salt |
|  | L-(+)-N-(p-(((2,4-Diamino-6-pteridinyl)methyl)methylamino)benzoyl)glutamate |
|  | Folex for injection |
|  | EINECS 231-022-0 |
|  | HDMTX |
|  | metothrexate sodium |
|  | Methotrexate, Dicesium Salt |
|  | Methotrexatum |
|  | emt-25299 |
|  | kyselina 4-desoxy-4-amino-n(sup 10)-methyllistova |
|  | emthexat |
|  | 2 [[4 [[(2,4 diaminopteridin 6 yl)methyl](methyl)amino]benzoyl]amino]pentanedioic acid |
|  | methotrate |
|  | Metatrexan |
|  | nordimet |
|  | Methotrexate |
|  | N-(4-[[(2,4-Diamino-6-pteridinyl)methyl](methyl)amino]benzoyl)glutamate |
|  | methotrexate gamma-mono t-butyl ester |
|  | 2 [[4 [[(2,4 diamino 6 pteridinyl)methyl](methyl)amino]benzoyl]amino]pentanedioic acid |
|  | 4-Amino-N10-methylpteroyl-L-glutamic acid |
|  | Methotrexate, (DL)-Isomer |
|  | N-(4-[[(2,4-Diamino-6-pteridinyl)methyl](methyl)amino]benzoyl)glutamic acid |
|  | N-(p-(((2,4-diamino-6-pteridinyl)methyl)methylamino)benzoyl)-Glutamic acid L-(+)-disodium salt |
|  | L-Glutamic acid, N-(4-(((2,4-diamino-6-pteridinyl)methyl)methylamino)benzoyl)-, sodium salt |
|  | Folex |
|  | CL-14377 |
|  | Folex-Pfs |
|  | mpi 5004 |
|  | texorate |
|  | A-Methpterin |
|  | Methotrexate, Sodium Salt |
|  | Methotrexate Hydrate |
|  | Kyselina 4-amino-N10-methylpteroylglutamova |
|  | canceren |
|  | poly(ethylene oxide)-block-poly(2-hydroxyethyl-L-aspartamide) |
|  | EMT 25,299 |
|  | NCI-C04671 |
|  | metrex |
|  | (+)-Amethopterin hydrate |
|  | n-(4-(((2,4-diamino-6-pteridinyl)methyl)methylamino)benzoyl)-l-glutamate |
|  | lantarel |
|  | metotrexate |
|  | N-(p-(((2,4-Diamino-6-pteridyl)methyl)methylamino)benzoyl)glutamic acid |
|  | 4-Amino-N(sup 10)-methylpteroylglutamate |
|  | n-(4-(((2,4-diamino-6-pteridinyl)methyl)methylamino)benzoyl)-l-glutamic acid |
|  | acide 4-amino 10-methyl folique |
|  | Sodium N-(4-(((2,4-diamino-6-pteridinyl)methyl)methylamino)benzoyl)-L-glutamate |
|  | l(+)-amethopterin dihydrate |
|  | [14C]methotrexate |
|  | emtrexate |
|  | Sodium L-(+)-methotrexate |
|  | meticil |
|  | emthexate |
|  | reumatrex |
|  | N-(4-(((2,4-Diamino-6-pteridinyl)methyl)methylamino)benzoyl)-DL-glutamate |
|  | 4-amino-n(10)-methylpteroylglutamate |
|  | methotrexate preservative free |
|  | Kyselina 4-desoxy-4-amino-N10-methyllistova |
|  | jylamvo |
|  | methotextrate |
|  | Amethopterin |
|  | [125l]methotrexate |
|  | N-(4-(((2,4-Diamino-6-pteridinyl)methyl)methylamino)benzoyl)-L-glutamicacid |
|  | methylaminopterine |
|  | metoject |
|  | 4-Aminomethylpteroylglutamic acid |
|  | zexate |
|  | methotrexate sodium preservative free |
|  | methotrexat |
|  | 4-Amino-10-methylfolate |
|  | N-(4-(((2,4-Diamino-6-pteridinyl)methyl)methylamino)benzoyl)-DL-glutamic acid |
|  | Methotrexate monohydrate |
|  | (DL)-Isomer of Methotrexate |
|  | novatrex |
|  | (+)-Amethopterin |
|  | HSDB 3123 |
|  | N-(p-(((2,4-Diamino-6-pteridyl)methyl)methylamino)benzoyl)glutamate |
|  | 15475-56-6 |
|  | methoblastin |
|  | 4-amino-n(10)-methylpteroylglutamic acid |
|  | methotrexate or mtx or rheumotrex |
|  | L-Amethopterin |
|  | n [4 [(2,4 diamino 6 pteridylmethyl)methylamino]benzoyl]glutamic acid |
|  | AI3-25299 |
|  | Disodium methotrexate |
|  | N-Bismethylpteroylglutamic acid |
|  | Methotrexate sodium (x-Na) |
|  | rasuvo |
|  | 4-Amino-N(sup 10)-methylpteroylglutamic acid disodium salt |
|  | Amethopterin sodium |
|  | methotrexate lpf |
|  | Kyselina N-(p-((2,4-diamino-6-pteridinylmethyl)methylamino)benzoyl)-L-glutamova |
|  | 4-Aminomethylpteroylglutamate |
|  | Methotrexate, Disodium Salt |
|  | Ledertrexate |
|  | Metotrexato |
|  | 59-05-2 |
|  | Antifolan |
|  | NSC-740 |
|  | methotrexate,human-mediated |
|  | metothrexate |
|  | methylaminopterin |
|  | IT MTS |
|  | biotrexate |
|  | 4-Amino-N(sup 10)-methylpteroylglutamic acid |
|  | L-(+)-N-(p-(((2,4-Diamino-6-pteridinyl)methyl)methylamino)benzoyl)glutamic acid |
|  | Disodium Salt of Methotrexate |
|  | [(3)H]methotrexate |
|  | Trexall |
|  | Dicesium Salt of Methotrexate |
|  | DL-Amethopterin |
|  | texate-t |
|  | Methotrexat dinatrium |
|  | metex |
|  | xatmep |
|  | xaken |
|  | N-(p-(((2,4-Diamino-6-pteridinyl)methyl)methylamino)benzoyl)-DL-glutamate |
|  | Rheumatrex |
|  | farmotrex |
|  | maxtrex |
|  | MeAPA-Glu |
|  | otrexup |
|  | Sodium methotrexate |
|  | n-(p-(((2,4-diamino-6-pteridinyl)methyl)methylamino)benzoyl)-l-()-glutamate |
|  | metotrexat |
|  | 4-Amino-10-methylfolic acid |
|  | n-(p-(((2,4-diamino-6-pteridinyl)methyl)methylamino)benzoyl)-l-()-glutamic acid |
|  | EINECS 262-213-7 |
|  | mexate-aq |
|  | L-Methotrexate |
|  | farmitrexate |
|  | neotrexate |
|  | A-Methopterin |
|  | texate |
|  | Methotrexate sodium |
|  | EINECS 200-413-8 |
|  | 4-amino-4-deoxy-n(sup 10)-methylpteroylglutamate |
|  | 60388-53-6 |
|  | rheumatrex dose pack |
|  | NSC 740 |
|  | Mexate |
|  | EINECS 239-495-5 |
|  | CCRIS 1109 |
|  | methrotrexate |
|  | 4-Amino-N10-methylpteroyl-L-glutamate |
|  | Methotrexat-Ebewe |
|  | metotrexin |
|  | 6745-93-3 |
|  | N-(p-(((2,4-Diamino-6-pteridinyl)methyl)methylamino)benzoyl)-L-(+)-glutamic acid |
|  | Metotressato |
|  | intradose MTX |
|  | 4 amino 10 methylpteroylglutamic acid |
|  | enthexate |
|  | Sodium Salt of Methotrexate |
|  | methoxtrexate |
|  | Farmitrexat |
|  | a methopterine |
|  | Abitrexate |
|  | MTX sodium |
|  | N-(p-(((2,4-Diamino-6-pteridinyl)methyl)methylamino)benzoyl)-DL-glutamic acid |
|  | mexate-aq preserved |
|  | DL-Methotrexate |
|  | Disodium N-(4-(((2,4-diamino-6-pteridinyl)methyl)methylamino)benzoyl)-L-glutamate |
|  | methotrexato |
|  | Kyselina 4-amino-N(sup 10)-methylpteroylglutamova |
|  | kyselinan-(p-((2,4-diamino-6-pteridinylmethyl)methylamino)benzoyl)-l-glutamova |
|  | n-(4((2,4-diamino-6-pteridinyl)methyl)methylamino)benzoyl)-l-glutamic |
|  | N-Bismethylpteroylglutamate |
|  | N-(4-(((2,4-diamino-6-pteridinyl)methyl)methylamino)benzoyl)-L-Glutamic acid sodium salt |
|  | antifolate amethopterin |
|  | ifamet |
|  | N-(p-(((2,4-diamino-6-pteridinyl)methyl)methylamino)benzoyl)-Glutamic acid sodium salt |
|  | NSC-117356 |
|  | amethopterine |
|  | 4-amino-4-deoxy-n(sup 10)-methylpteroylglutamic acid |
|  | 7413-34-5 |
|  | MTX disodium |

---

|  |  |
| --- | --- |
| CAS ID | 59-05-2 |
|  | 1082707-84-3 |
|  | 7413-34-5 |
|  | 15475-56-6 |
|  | 60388-53-6 |
|  | 6745-93-3 |

---

|  |  |
| --- | --- |
| Reaxys ID | 12976500 |
|  | 15191926 |
|  | 4219876 |
|  | 4644426 |
|  | 4730582 |
|  | 4730583 |
|  | 5224471 |
|  | 5678284 |
|  | 6042366 |
|  | 6045736 |
|  | 70669 |
|  | 9171329 |
|  | 9600914 |

---

|  |  |
| --- | --- |
| ChEBI ID | 44185 |

---

|  |  |
| --- | --- |
| PharmaPendium ID | Methotrexate Sodium |

---

|  |  |
| --- | --- |
| HMDB ID | HMDB14703 |

---

|  |  |
| --- | --- |
| KEGG ID | C01937 |

---

|  |  |
| --- | --- |
| InChIKey | FBOZXECLQNJBKD-ZDUSSCGKSA-N |
|  | FBOZXECLQNJBKD-UHFFFAOYSA-N |
|  | FPJYMUQSRFJSEW-ZOWNYOTGSA-N |
|  | DASQOOZCTWOQPA-GXKRWWSZSA-L |
|  | BKBBTCORRZMASO-ZOWNYOTGSA-M |

---

|  |  |
| --- | --- |
| Molecular Formula | C20H22N8O5 |
|  | C20H24N8O6 |
|  | C20H20N8Na2O5 |
|  | C20H21N8NaO5 |

---

|  |  |
| --- | --- |
| PubChem SID | 134972286 |
|  | 134987860 |
|  | 135018907 |
|  | 135065271 |
|  | 135122321 |

---

|  |  |
| --- | --- |
| PubChem CID | 126941 |
|  | 11329481 |
|  | 23678981 |
|  | 4112 |
|  | 165528 |

---

|  |  |
| --- | --- |
| IUPAC Name | 2-[[4-[(2,4-diaminopteridin-6-yl)methyl-methyl-amino]benzoyl]amino]glutaric acid |
|  | (2S)-2-[[4-[(2,4-diaminopteridin-6-yl)methyl-methyl-amino]benzoyl]amino]glutaric acid |
|  | (2S)-2-[[4-[(2,4-diaminopteridin-6-yl)methyl-methyl-amino]benzoyl]amino]glutaric acid;hydrate |
|  | disodium;(2S)-2-[[4-[(2,4-diaminopteridin-6-yl)methyl-methyl-amino]benzoyl]amino]glutarate |
|  | sodium;(4S)-4-[[4-[(2,4-diaminopteridin-6-yl)methyl-methyl-amino]benzoyl]amino]-5-hydroxy-5-keto-valerate |

---

|  |  |
| --- | --- |
| Rotatable Bond Count | 9 |
|  | 7 |

---
