# Supplementary material for: Analysis of PPARγ Signaling Activity in Psoriasis
Source: Int J Mol Sci. 2021 Aug 10;22(16):8603. doi: 10.3390/ijms22168603 (PMC8395241; doi:10.3390/ijms22168603)
Supplement: Supplementary file 1 [file ijms-22-08603-s001.zip › Supplemental materials_Analysis of PPARg signaling activity in psoriasis/Pathway models/Models images and html files/Anti-psoriatic drugs influence PPARG signaling/48185.html]

dexamethasone


# Small Molecule dexamethasone

|  |  |
| --- | --- |
| URN | urn:agi-cas:50-02-2 |
| Total Entities | 17 |
| Connectivity | 9094 |
| Name | dexamethasone |
| Molecular Weight | 392.461063 |
| XLogP | 1.900000 |
| ObjectType | Small Molecule |

---

|  |  |
| --- | --- |
| ChildConcepts | betamethasone 17-benzoate |
|  | triamcinolone |
|  | fludrocortisone |
|  | halometasone |
|  | fluocortolone |
|  | isoflupredone |
|  | desoximetasone |
|  | dexamethasone palmitate |
|  | dexamethasone valerate |
|  | fluocinolone |
|  | betamethasone 17-propionate |
|  | THS-201 |
|  | flumethasone |
|  | U-42129 |
|  | diflucortolone |
|  | methylprednisolone |
|  | paramethasone |

---

|  |  |
| --- | --- |
| Pathway | B-Cell Acute Lymphoblastic Leukemia |
|  | T-Cell Acute Lymphoblastic Leukemia |
|  | MYOC Associated Glaucoma |
|  | Dexamethasone Induced Neurotoxicity |
|  | Dexamethasone Induced Diabetes |
|  | Dexamethasone Induced Osteoporosis |
|  | hemophylia |
|  | phenylketonuria |
|  | Figure 3\_Mixed network of dexamethasone targets |
|  | Figure 3\_Mixed network of dexamethasone targets |
|  | New Pathway |
|  | Anti-psoriatic drugs influence PPARG signaling |

---

|  |  |
| --- | --- |
| MedScan ID | 1211077 |

---

|  |  |
| --- | --- |
| Alias | 1-dehydro-16alpha-methyl-0alpha-fluorohydrocortisone |
|  | Prednisolone F |
|  | glucocorticoid desametasone |
|  | dexaclinit |
|  | decdan |
|  | hexadiol |
|  | decadron 5-12 pak |
|  | Dexametasona Belmac |
|  | Dexametasona |
|  | (3H)-Dexamethasone (labeled with tritium) |
|  | ozurdex |
|  | oftan-dexa |
|  | Hexadreson |
|  | sterasone |
|  | posurdex |
|  | decaesadril |
|  | Dexa Mamallet |
|  | desadrene |
|  | Azium |
|  | Dexamethasone |
|  | PMS Dexamethasone |
|  | dexason |
|  | dexa clinit |
|  | dexamethasone Monofree |
|  | Azupharma, dexamethasone |
|  | dexamethasone FNA |
|  | methazonione |
|  | 9-Fluoro-11,17,21-trihydroxy-16-methylpregna-1,4-diene-3,20-dione |
|  | Belmac, Dexametasona |
|  | isnacort |
|  | dexasone s |
|  | Monofree, dexamethasone |
|  | dexona |
|  | orgadrone |
|  | dexahelvacort |
|  | prodexone |
|  | Hexadecadrol |
|  | decilone |
|  | Aphtasolon |
|  | HSDB 3053 |
|  | 21-trihydroxy-1,4-pregnadiene-3,20-dione |
|  | doxamethasone |
|  | Dexazone |
|  | Dexaphos |
|  | Millicorten |
|  | Dexone |
|  | mephameson |
|  | neoforderx |
|  | Desamethasone |
|  | Dexahexal |
|  | mediamethasone |
|  | Methasone |
|  | Naquasone (Veterinary) |
|  | oradexone |
|  | Calonat |
|  | DexamethasonRotexmedica |
|  | Dexa-Cortidelt |
|  | semisynthetic glucocorticoid |
|  | dexascheroson |
|  | aflucosone |
|  | sanamethasone |
|  | DexaEDO |
|  | dexinoral |
|  | dexadrol |
|  | decadeltosone |
|  | exadione |
|  | Dexone L.A. |
|  | DEX PS DDS |
|  | MK-125 |
|  | delladec |
|  | sawasone |
|  | Decadron Topical Cream |
|  | decadran |
|  | dexascherozon |
|  | Dekasol |
|  | methazone ion |
|  | dergramin |
|  | Decaspray |
|  | dexameson |
|  | dexamesone |
|  | dibasona |
|  | prodexona |
|  | Dexapent |
|  | gammacortene |
|  | Fulvidex (Veterinary) |
|  | isopto-maxidex |
|  | AI3-50934 |
|  | dexa korti |
|  | ratioDexamethasone |
|  | arcodexane |
|  | Dexagalen |
|  | SK-Dexamethasone |
|  | VAD doxil |
|  | Dexafarma |
|  | cortastat |
|  | Bisu DS |
|  | desigdron |
|  | Desameton |
|  | DexaAllvoran |
|  | adrenocot |
|  | methazonion |
|  | dezone |
|  | Decaderm |
|  | dextenza |
|  | Dexapos |
|  | Decalix |
|  | dexamethasone Azupharma |
|  | dexacort |
|  | cortastat la |
|  | pidexon |
|  | luxazone |
|  | Noriplon |
|  | (11beta,16alpha)-9-Fluoro-11,17,21-trihydroxy-16-methylpregna-1,4-diene-3,20-dione |
|  | Dexa-Cortisyl |
|  | dexone 4 |
|  | dekacort |
|  | IsoptoDex |
|  | Hexadrol |
|  | dexamethasone GALEN |
|  | 9-alpha-Fluoro-16-alpha-methyl-1,4-pregnadiene-11-beta,17-alpha,21-triol-3,20-dione |
|  | 9alpha-fluoro-16alpha-methyl-Prednisolone |
|  | opticorten |
|  | dexamethasone Jenapharm |
|  | ratio Dexamethasone |
|  | decasterolone |
|  | visumethazone |
|  | fluormethyl prednisolone |
|  | grosodexone |
|  | Siozwo, Dexa |
|  | dexascherozone |
|  | Auxiron |
|  | delta(sup 1)-9-alpha-Fluoro-16-alpha-methylcortisol |
|  | dexamecortin |
|  | millicortenol |
|  | 9alpha-Fluoro-16alpha-methyl-11beta,17alpha,21-trihydroxy-1,4-pregnadiene-3,20-dione |
|  | vexamet |
|  | dalalone d.p. |
|  | 16-alpha-Methyl-9-alpha-fluoro-1,4-pregnadiene-11-beta,17-alpha,21-triol-3,20-dione |
|  | dexamethonium |
|  | Dexa-Scheroson |
|  | dacortine fuerte |
|  | Dexsone |
|  | 9-fluoro-11-beta,17,21-trihydroxy-16-alpha-methyl-pregna-1,4-diene-3,20-dione |
|  | mymethasone |
|  | firmalone |
|  | desalark |
|  | dexachel |
|  | Dexa Jenapharm |
|  | thilodexine |
|  | 16-alpha-Methyl-9-alpha-fluoro-1-dehydrocortisol |
|  | Mexidex |
|  | dexone 1.5 |
|  | Dexagrin |
|  | metisone lafi |
|  | Dexametasona, Oft Cusi |
|  | Dexamethasonum |
|  | oradexon |
|  | dexycu |
|  | Dexa Allvoran |
|  | Dexacortin |
|  | 9alpha fluoro 16alpha methyl delta corticosterone |
|  | Dinormon |
|  | dalalone l.a. |
|  | 9-Fluoro-11-beta,17,21-trihydroxy-16-alpha-methylpregna-1,4-diene-3,20-dione |
|  | fluoromethylprednisolone |
|  | cortisumman |
|  | Decagel |
|  | dexa scherozone |
|  | dexone 0.75 |
|  | Desametasone |
|  | decadrone |
|  | [3H]desamethasone |
|  | Intensol, Dexamethasone |
|  | Dexaflam |
|  | DexaEffekton |
|  | 9alpha fluoro 11beta,17alpha,21 trihydroxy 16alpha methylpregna 1,4 diene 3,20 dione |
|  | de-sone la |
|  | predni f tablinen |
|  | CCRIS 7067 |
|  | isoptomaxidex |
|  | Dexamethasonmp |
|  | decadion |
|  | desacortone |
|  | santeson |
|  | grosodexon |
|  | EINECS 200-003-9 |
|  | artrosone |
|  | Dexpak |
|  | decilone forte |
|  | Sunia Sol D |
|  | metasolon |
|  | predni-f |
|  | 16alpha-methyl-9alpha-fluoro-1,4-pregnadiene-11beta,17alpha,21-triol-2,20-dione |
|  | solurex la |
|  | DexaPhlogont |
|  | oradexan |
|  | dacortina fuerte |
|  | delta1-9alpha-Fluoro-16alpha-methylcortisol |
|  | DexaSine |
|  | Spoloven |
|  | adrecort |
|  | DRG-0013 |
|  | alfalyl |
|  | Methazon-Ion |
|  | 16-alpha-Methyl-9-alpha-fluoro-delta1-hydrocortisone |
|  | (3H)-Dexamethasone |
|  | Dexa Effekton |
|  | dexame |
|  | Visumetazone |
|  | Dexalona |
|  | Dexamonozon |
|  | GALEN, dexamethasone |
|  | fluormethylprednisolon |
|  | Superprednol |
|  | santenson |
|  | Jenapharm, Dexa |
|  | marvidione |
|  | FNA, dexamethasone |
|  | 16-alpha-Methyl-9-alpha-fluoro-delta(sup 1)-hydrocortisone |
|  | ex s1 |
|  | Corsone |
|  | novocort |
|  | dexapot |
|  | dexamethasone mp |
|  | 23495-06-9 |
|  | Dexatad |
|  | Dexamedium |
|  | corsona |
|  | 9-alpha-Fluoro-16-alpha-methylprednisolone |
|  | deronil |
|  | decasone |
|  | Solutio cordes Dexa N |
|  | dexa scherosan |
|  | 9alpha fluoro 11beta,17alpha,21 trihydroxy 16alpha methyl 1,4 pregnadiene 3,20 dione |
|  | dexane |
|  | dalalone |
|  | dexa scherozon |
|  | 137098-19-2 |
|  | Decadron Inhalation |
|  | cebedex |
|  | Dexa Phlogont |
|  | Dexadeltone |
|  | PredniFTablinen |
|  | decofluor |
|  | dexacortal |
|  | Solupen |
|  | 9-fluoro-11beta,17,21-trihydroxy-16a-methylpregna-1,4-diene-3,20-dione |
|  | Loscon, Dexa |
|  | cortidrone |
|  | dexionil |
|  | Decadron Elixir |
|  | cortastat 10 |
|  | Deseronil |
|  | L.A., Dexone |
|  | aeroseb-dex |
|  | Decadron |
|  | Dexa Siozwo |
|  | danasone |
|  | aflucoson |
|  | Deltafluorene |
|  | Decameth |
|  | 8054-59-9 |
|  | Dexaratiopharm |
|  | dextrasone |
|  | arcodexan |
|  | dexan |
|  | 16-alpha-Methyl-9-alpha-fluoro-11-beta,17-alpha,21-trihydroxypregna-1,4-diene-3,20-dione |
|  | Dexavene |
|  | bidexol |
|  | 50-02-2 |
|  | dexmethsone |
|  | fluorocort |
|  | mexasone |
|  | Cusi Dexametasona, Oft |
|  | dexa-p |
|  | Fluormethylprednisolone |
|  | Cortamethasone |
|  | DekasolL.A. |
|  | Hexadrol Elixir |
|  | HL-Dex |
|  | cortidex |
|  | megacortin |
|  | Dexaprol |
|  | dexalien |
|  | cortidrona |
|  | decamethasone |
|  | Dexa Pos |
|  | 16alpha methyl 9alpha fluoroprednisolone |
|  | Dexasone |
|  | dexadecadrol |
|  | dexano |
|  | Decadron Tablets |
|  | Policort |
|  | 1-Dehydro-16-alpha-methyl-9-alpha-fluorohydrocortisone |
|  | dexa dabrosan |
|  | Dexasel |
|  | decadeltosona |
|  | Jenapharm, dexamethasone |
|  | Dexinolon |
|  | dexpak taperpak |
|  | dexone 0.5 |
|  | (11beta,16alpha)-9-fluoro-11,17,21-trihydroxy-16-methyl-Pregna-1,4-diene-3,20-dione |
|  | nisomethasona |
|  | Dexoral |
|  | desacort |
|  | Stmerin-D Azimycin |
|  | opticortinol |
|  | Dexapolcort |
|  | Lipotalon |
|  | dexacortisyl |
|  | Dexabene |
|  | fluorodelta |
|  | Dexamethazone |
|  | Dexamet |
|  | hexadecadiol |
|  | dextelan |
|  | dectancyl |
|  | cetadexon |
|  | deltafluoren |
|  | m-ACOD |
|  | lokalison f |
|  | dexacorten |
|  | triamcimetil |
|  | Gammacorten |
|  | metasolone |
|  | dexasone la |
|  | dexamethasone Rotexmedica |
|  | Aeroseb-D |
|  | Dexa ratiopharm |
|  | dexagel |
|  | Decacortin |
|  | Dex-ide |
|  | dexakorti |
|  | NSC 34521 |
|  | Dexamethasone alcohol |
|  | loverine |
|  | Anaflogistico |
|  | Dexabeta |
|  | dexacen 4 |
|  | Dekasol L.A. |
|  | cortidexason |
|  | dexagen |
|  | fluormone |
|  | Oft Cusi Dexametasona |
|  | neofordex |
|  | m-VAD |
|  | dexadabroson |
|  | exadion |
|  | 4-alpha-Fluoro-16-alpha-methyl-11-beta,17,21-trihydroxypregna-1,4-diene-3,20-dione |
|  | PMSDexamethasone |
|  | (11beta,16alpha)-9-Fluoro-11,17,21-trihydroxy-16-methylpregna-1,4-diene-3,20-dione labeled with tritium |
|  | esacortene |
|  | desametone |
|  | isopto-dex |
|  | Dexa Sine |
|  | (11beta,16alpha)-9-fluoro-11,17,21-trihydroxy-16-methyl-Pregna-1,4-diene-3,20-dione labeled with tritium |
|  | Methylfluorprednisolone |
|  | fortecortin |
|  | Indarzona |
|  | Dexa Loscon |
|  | anaflogistico novobios |
|  | Dexameth |
|  | Dexamethasone Intensol |
|  | dexalocal |
|  | Decacort |
|  | dexametason |
|  | Dexamethason Monofree |
|  | Dexamethason Rotexmedica |
|  | GALEN, Dexamethason |
|  | dexamethazon |
|  | dexamethason |
|  | Azupharma, Dexamethason |
|  | Dexamethason Azupharma |
|  | FNA, Dexamethason |
|  | Monofree, Dexamethason |
|  | Dexamethason mp |
|  | Prednisolon F |
|  | Dexamethason FNA |
|  | dexametasone |
|  | Dexamethason GALEN |
|  | Dexamethason Jenapharm |
|  | Jenapharm, Dexamethason |

---

|  |  |
| --- | --- |
| CAS ID | 50-02-2 |
|  | 1050677-47-8 |
|  | 137098-19-2 |
|  | 8054-59-9 |
|  | 906362-70-7 |
|  | 906422-84-2 |
|  | 23495-06-9 |

---

|  |  |
| --- | --- |
| Reaxys ID | 2066646 |
|  | 2066647 |
|  | 2066648 |
|  | 2066649 |
|  | 2066650 |
|  | 2066651 |
|  | 2066652 |
|  | 2228630 |
|  | 2228631 |
|  | 2228632 |
|  | 2341697 |
|  | 2341698 |
|  | 2711208 |
|  | 3040476 |
|  | 3176546 |
|  | 3657441 |
|  | 3657442 |
|  | 4847476 |
|  | 5311709 |
|  | 5775258 |
|  | 6075646 |

---

|  |  |
| --- | --- |
| ChEBI ID | 41879 |

---

|  |  |
| --- | --- |
| PharmaPendium ID | Dexamethasone |

---

|  |  |
| --- | --- |
| HMDB ID | HMDB15364 |
|  | HMDB30049 |

---

|  |  |
| --- | --- |
| InChIKey | UREBDLICKHMUKA-CXSFZGCWSA-N |

---

|  |  |
| --- | --- |
| Molecular Formula | C22H29FO5 |

---

|  |  |
| --- | --- |
| PubChem SID | 134970849 |
|  | 135020767 |

---

|  |  |
| --- | --- |
| PubChem CID | 5743 |

---

|  |  |
| --- | --- |
| IUPAC Name | (8S,9R,10S,11S,13S,14S,16R,17R)-9-fluoro-17-glycoloyl-11,17-dihydroxy-10,13,16-trimethyl-6,7,8,11,12,14,15,16-octahydrocyclopenta[a]phenanthren-3-one |

---

|  |  |
| --- | --- |
| Rotatable Bond Count | 2 |

---
