# Supplementary material for: Analysis of PPARγ Signaling Activity in Psoriasis
Source: Int J Mol Sci. 2021 Aug 10;22(16):8603. doi: 10.3390/ijms22168603 (PMC8395241; doi:10.3390/ijms22168603)
Supplement: Supplementary file 1 [file ijms-22-08603-s001.zip › Supplemental materials_Analysis of PPARg signaling activity in psoriasis/Pathway models/Models images and html files/Anti-psoriatic drugs influence PPARG signaling/55681.html]

atorvastatin


# Small Molecule atorvastatin

|  |  |
| --- | --- |
| URN | urn:agi-cas:110862-48-1 |
| Total Entities | 1 |
| Connectivity | 3296 |
| Name | atorvastatin |
| Molecular Weight | 558.650000 |
| XLogP | 5.678400 |

---

|  |  |
| --- | --- |
| ChildConcepts | AM-682 |

---

|  |  |
| --- | --- |
| Pathway | Anti-psoriatic drugs influence PPARG signaling |

---

|  |  |
| --- | --- |
| MedScan ID | 1223066 |

---

|  |  |
| --- | --- |
| Alias | Atorvastatin (R-(R\*,R\*)) |
|  | Atorvastatin |
|  | astator |
|  | DRG-0321 |
|  | (R\*,R\*)-2-(4-fluorophenyl)-beta,delta-dihydroxy-5-(1-methylethyl)-3-phenyl-4-((phenylamino)carbonyl)-1H-Pyrrole-1-heptanoic acid |
|  | Sotis |
|  | Liprimar |
|  | [(14)C]atorvastatin |
|  | lipibec |
|  | orbeos |
|  | Xavator |
|  | Calcium (betaR,deltaR)-2-(p-fluorophenyl)-beta,delta-dihydroxy-5-isopropyl-3-phenyl-4-(phenylcarbamoyl)pyrrole-1-heptanoate (1:2) |
|  | 134523-03-8 |
|  | PD 134298-38A |
|  | (betaR,deltaR)-rel-2-(4-fluorophenyl)-beta,delta-dihydroxy-5-(1-methylethyl)-3-phenyl-4-((phenylamino)carbonyl)-1H-Pyrrole-1-heptanoate |
|  | 2 (4 fluorophenyl) beta,delta dihydroxy 5 isopropyl 3 phenyl 4 phenylcarbamoyl 1h pyrrole 1 heptanoic acid |
|  | Atovastatin |
|  | atostin |
|  | atorvastatin calcium trihydrate |
|  | atoris |
|  | Atorvastatin calcium salt |
|  | (1betaR,deltaR)-2-(p-Fluorophenyl)-beta,delta-dihydroxy-5-isopropyl-3-phenyl-4-(phenylcarbamoyl)pyrrole-1-heptanoic acid |
|  | statorva |
|  | (betaR,deltaR)- 2-(4-fluorophenyl)-beta,delta-dihydroxy-5-(1-methylethyl)-3-phenyl-4-((phenylamino)carbonyl)-1H-Pyrrole-1-heptanoic acid |
|  | (betaR,deltaR)-2-(p-Fluorophenyl)-beta,delta-dihydroxy-5-isopropyl-3-phenyl-4-(phenylcarbamoyl)pyrrole-1-heptanoic acid |
|  | 7-[[2-(4-fluorophenyl)-5-isopropyl-3-phenyl-4-(phenylcarbamoyl)-1H-pyrrol-1-yl]]-3,5-dihydroxy-heptanoic acid |
|  | (R\*,R\*)-2-(4-fluorophenyl)-beta,delta-dihydroxy-5-(1-methylethyl)-3-phenyl-4-((phenylamino)carbonyl)-1H-Pyrrole-1-heptanoate |
|  | Atorvastatin (R\*,R\*) |
|  | storvas |
|  | ator |
|  | 110862-48-1 |
|  | Atorvastan |
|  | liptonorm |
|  | (R-(R\*,R\*))-2-(4-fluorophenyl)-beta,delta-dihydroxy-5-(1-methylethyl)-3-phenyl-4-((phenylamino)carbonyl)-1H-Pyrrole-1-heptanoic acid calcium salt (2:1) |
|  | 7-[[2-(4-fluorophenyl)-5-isopropyl-3-phenyl-4-(phenylcarbamoyl)-1H-pyrrol-1-yl]]-3,5-dihydroxy-heptanoate |
|  | CCRIS 7159 |
|  | atovarol |
|  | Atorlip |
|  | (R-(R\*,R\*))-2-(4-Fluorophenyl)-beta,delta-dihydroxy-5-(1-methylethyl)-3-phenyl-4-((phenylamino)carbonyl)-1H-pyrrole-1-heptanoate |
|  | Torvast |
|  | Atorvastatin calcium |
|  | zarator |
|  | atorab |
|  | glustar |
|  | obradon |
|  | Totalip |
|  | lowlipen |
|  | 134523-00-5 |
|  | (1betaR,deltaR)-2-(p-Fluorophenyl)-beta,delta-dihydroxy-5-isopropyl-3-phenyl-4-(phenylcarbamoyl)pyrrole-1-heptanoate |
|  | Tozalip |
|  | HSDB 7039 |
|  | CI 981 |
|  | (R-(R\*,R\*))-beta,delta-dihydroxy-2-(4-fluorophenyl)-5-(1-methylethyl)-3-phenyl-4-((phenylamino)carbonyl)-1H-Pyrrole-1-heptanoic acid calcium salt (2:1) |
|  | Sortis |
|  | atovans |
|  | tahor |
|  | (betaR,deltaR)- 2-(4-fluorophenyl)-beta,delta-dihydroxy-5-(1-methylethyl)-3-phenyl-4-((phenylamino)carbonyl)-1H-Pyrrole-1-heptanoate |
|  | CI-981 |
|  | (R-(R\*,R\*))-2-(4-Fluorophenyl)-beta,delta-dihydroxy-5-(1-methylethyl)-3-phenyl-4-((phenylamino)carbonyl)-1H-pyrrole-1-heptanoic acid |
|  | Atorvistatin |
|  | atorvastine |
|  | calcium (betar,dr)-2-(p-fluorophenyl)-beta,d-dihydroxy-5-isopropyl-3-phenyl-4-(phenylcarbamoyl)pyrrole-1-heptanoate |
|  | (betaR,deltaR)-rel-2-(4-fluorophenyl)-beta,delta-dihydroxy-5-(1-methylethyl)-3-phenyl-4-((phenylamino)carbonyl)-1H-Pyrrole-1-heptanoic acid |
|  | prevencor |
|  | (betaR,deltaR)-2-(p-Fluorophenyl)-beta,delta-dihydroxy-5-isopropyl-3-phenyl-4-(phenylcarbamoyl)pyrrole-1-heptanoate |
|  | ym548 |
|  | atorvadivid |
|  | calcium 1-(6-carboxylato-3,5-dihydroxy-hexyl)-5-(4-fluorophenyl)-2-isopropyl-N,4-diphenyl-1H-pyrrole-3-carboxamide |
|  | Lipitor |
|  | xarator |
|  | Cardyl |

---

|  |  |
| --- | --- |
| CAS ID | 110862-48-1 |
|  | 1108202-55-6 |
|  | 134523-03-8 |
|  | 334757-04-9 |
|  | 134523-00-5 |

---

|  |  |
| --- | --- |
| Reaxys ID | 14456345 |
|  | 14531466 |
|  | 15443248 |
|  | 5373842 |
|  | 8373630 |

---

|  |  |
| --- | --- |
| ChEBI ID | 39548 |
|  | 50686 |

---

|  |  |
| --- | --- |
| PharmaPendium ID | Atorvastatin Calcium |

---

|  |  |
| --- | --- |
| HMDB ID | HMDB05006 |

---

|  |  |
| --- | --- |
| KEGG ID | C06834 |

---

|  |  |
| --- | --- |
| InChIKey | XUKUURHRXDUEBC-KAYWLYCHSA-N |
|  | FQCKMBLVYCEXJB-MNSAWQCASA-L |
|  | XUKUURHRXDUEBC-SVBPBHIXSA-N |

---

|  |  |
| --- | --- |
| Molecular Formula | C33H35FN2O5 |
|  | C66H68CaF2N4O10 |

---

|  |  |
| --- | --- |
| PubChem SID | 135017553 |
|  | 135019185 |
|  | 135260057 |

---

|  |  |
| --- | --- |
| PubChem CID | 60822 |
|  | 60823 |
|  | 62976 |

---

|  |  |
| --- | --- |
| XLogP-AA | 5 |

---

|  |  |
| --- | --- |
| IUPAC Name | calcium;(3R,5R)-7-[2-(4-fluorophenyl)-5-isopropyl-3-phenyl-4-(phenylcarbamoyl)pyrrol-1-yl]-3,5-dihydroxy-enanthate |
|  | (3R,5R)-7-[2-(4-fluorophenyl)-5-isopropyl-3-phenyl-4-(phenylcarbamoyl)pyrrol-1-yl]-3,5-dihydroxy-enanthic acid |
|  | (3S,5S)-7-[2-(4-fluorophenyl)-5-isopropyl-3-phenyl-4-(phenylcarbamoyl)pyrrol-1-yl]-3,5-dihydroxy-enanthic acid |

---

|  |  |
| --- | --- |
| Rotatable Bond Count | 22 |
|  | 12 |

---
