# Supplementary material for: Analysis of PPARγ Signaling Activity in Psoriasis
Source: Int J Mol Sci. 2021 Aug 10;22(16):8603. doi: 10.3390/ijms22168603 (PMC8395241; doi:10.3390/ijms22168603)
Supplement: Supplementary file 1 [file ijms-22-08603-s001.zip › Supplemental materials_Analysis of PPARg signaling activity in psoriasis/Pathway models/Models images and html files/Anti-psoriatic drugs influence PPARG signaling/56904.html]

tacrolimus


# Small Molecule tacrolimus

|  |  |
| --- | --- |
| URN | urn:agi-cas:104987-11-3 |
| Total Entities | 4 |
| Connectivity | 4173 |
| Name | tacrolimus |
| Molecular Weight | 822.046000 |
| XLogP | 2.700000 |
| ObjectType | Small Molecule |

---

|  |  |
| --- | --- |
| ChildConcepts | pimecrolimus |
|  | ABT-281 |
|  | L-685,818 |
|  | ascomycin |

---

|  |  |
| --- | --- |
| Pathway | beta-Cell Function Inhibition by Ciclosporine and Tacrolimus (Rodent Model) |
|  | AREG -> NFATC Expression Target |
|  | BTC -> NFATC Expression Targets |
|  | EGF -> MEF/MYOD/NFATC Expression Targets |
|  | EGFR/ERBB3 -> MEF/MYOD/NFATC/MYOG Signaling |
|  | HBEGF -> MEF/MYOD Expression Target |
|  | NRG1 -> MEF/MYOD Expression Targets |
|  | AVP/Gs -> MEF/MYOD/NFATC/MYOG Expression Targets |
|  | AVP/Gq -> MEF/MYOD/NFATC/MYOG Expression Targets |
|  | TGFA -> MEF/MYOD/NFATC Expression Targets |
|  | Anti-psoriatic drugs influence PPARG signaling |

---

|  |  |
| --- | --- |
| MedScan ID | 1224796 |

---

|  |  |
| --- | --- |
| Alias | Tacrolimus anhydrous |
|  | (-)-FK 506 |
|  | 104987-11-3 |
|  | Astagraf XL |
|  | envarsus xr |
|  | Tacrolimus monohydrate |
|  | macrolide FK 506 |
|  | Tacrolimus hydrate |
|  | L 679934 |
|  | FK 506 |
|  | CCRIS 7124 |
|  | macrolide lactone |
|  | [3H]tacrolimus |
|  | modigraf |
|  | FK506 |
|  | Fujimycin |
|  | tacforius |
|  | immunosuppressants FK506 |
|  | 109581-93-3 |
|  | protopic ointment |
|  | advagraf |
|  | FK-506 |
|  | mustopic oint |
|  | Prograff |
|  | (-)FK-506 |
|  | SKF 506 |
|  | sterling 37000 |
|  | Tsukubaenolide |
|  | Anhydrous Tacrolimus |
|  | prograft |
|  | envarsus |
|  | Protopic |
|  | hecoria |
|  | Tsukubaenolide hydrate |
|  | protopy |
|  | FK 506-tacrolimus |
|  | SKF-506 |
|  | 15,19-epoxy-3h-pyrido(2,1-c)(1,4)oxaazacyclotri cosine-1,7,20,21(4h,23h)-tetrone,5,6,8,11,12,13,14,15,16,17,18,19 |
|  | Prograf |
|  | Tacrolimus |
|  | FR-900506 |

---

|  |  |
| --- | --- |
| CAS ID | 104987-11-3 |
|  | 109581-93-3 |

---

|  |  |
| --- | --- |
| Reaxys ID | 3647477 |
|  | 4290083 |
|  | 4290084 |
|  | 4902953 |
|  | 6265275 |
|  | 8383646 |

---

|  |  |
| --- | --- |
| PharmaPendium ID | Tacrolimus |

---

|  |  |
| --- | --- |
| HMDB ID | HMDB15002 |

---

|  |  |
| --- | --- |
| InChIKey | QJJXYPPXXYFBGM-LFZNUXCKSA-N |
|  | NWJQLQGQZSIBAF-MLAUYUEBSA-N |

---

|  |  |
| --- | --- |
| Molecular Formula | C44H69NO12 |
|  | C44H71NO13 |

---

|  |  |
| --- | --- |
| PubChem SID | 135016976 |
|  | 135022992 |

---

|  |  |
| --- | --- |
| PubChem CID | 5282315 |
|  | 445643 |

---

|  |  |
| --- | --- |
| Rotatable Bond Count | 7 |

---
