# Supplementary material for: Analysis of PPARγ Signaling Activity in Psoriasis
Source: Int J Mol Sci. 2021 Aug 10;22(16):8603. doi: 10.3390/ijms22168603 (PMC8395241; doi:10.3390/ijms22168603)
Supplement: Supplementary file 1 [file ijms-22-08603-s001.zip › Supplemental materials_Analysis of PPARg signaling activity in psoriasis/Pathway models/Models images and html files/Anti-psoriatic drugs influence PPARG signaling/6391.html]

IL17A


# Protein IL17A

|  |  |
| --- | --- |
| URN | urn:agi-llid:3605 |
| Total Entities | 0 |
| Connectivity | 8173 |
| Name | IL17A |
| Description | interleukin 17A |
| Notes | The protein encoded by this gene is a proinflammatory cytokine produced by activated T cells. This cytokine regulates the activities of NF-kappaB and mitogen-activated protein kinases. This cytokine can stimulate the expression of IL6 and cyclooxygenase-2 (PTGS2/COX-2), as well as enhance the production of nitric oxide (NO). High levels of this cytokine are associated with several chronic inflammatory diseases including rheumatoid arthritis, psoriasis and multiple sclerosis. [provided by RefSeq, Jul 2008] |
| Primary Cell Localization | Extracellular |
| Class | Ligand |

---

|  |  |
| --- | --- |
| Pathway | Melatonin Effects on Circadian Cycle |
|  | AHR Signaling in Treg and Dendritic Cells Function |
|  | AHR Signaling in Th17 Cells Function |
|  | Th17-Cell Differentiation |
|  | AHR in Intestinal Cell Antimicrobial Barrier Maintenance |
|  | Nociception Expression Targets Signaling |
|  | PTGER2/3 -> Inflammation-Related Expression Targets |
|  | Proteins Involved in Atherosclerosis |
|  | Proteins Involved in Dilated Cardiomyopathy |
|  | Lymphocyte Mediated Myocardial Injury in Myocarditis |
|  | Proteins Involved in Myocarditis |
|  | IL17 Signaling in Psoriasis |
|  | Proteins Involved in Psoriasis |
|  | T-Cells Differentiation Block in Psoriasis |
|  | Proteins Involved in Systemic Lupus Erythematosus |
|  | Th17-Cell Function in Systemic Lupus Erythematosus |
|  | non-Suppressive Treg-Cell in Diabetes Mellitus Type 1 |
|  | Proteins Involved in non-Alcoholic Fatty Liver Disease |
|  | Proteins Involved in Obesity |
|  | Proteins Involved in Colorectal Neoplasms |
|  | Paneth-Cell Function in Crohn's Disease |
|  | Th17-Cell Activation in Crohn's Disease |
|  | Proteins Involved in Inflammatory Bowel Diseases |
|  | Proteins Involved in Helicobacter Infections |
|  | Proteins Involved in Ulcerative Colitis |
|  | Proteins Involved in Multiple Myeloma |
|  | Proteins Involved in Hepatocellular Carcinoma |
|  | Il17 Signaling Related Neutrophilia in Asthma |
|  | Airway Smooth Muscle Cell Contraction |
|  | Th17-Cell Differentiation in Asthma |
|  | Th17-Cell and Th1 Immune Responsein Psoriatic Arthritis |
|  | Synovial Fibroblast Activation in Psoriatic Arthritis |
|  | Keratinocyte Activation in Psoriatic Arthritis |
|  | Proteins Involved in Psoriatic Arthritis |
|  | Synovial Fibroblast Activation by Citokines in Rheumatoid Arthritis |
|  | Proteins Involved in Rheumatoid Arthritis |
|  | Proteins Involved in Systemic Scleroderma |
|  | Th17-Cell Numbers Reduction in HIV |
|  | Proteins Involved in Periodontitis |
|  | Proteins Involved in Osteoporosis |
|  | Proteins with Altered Expression in Psoriatic Arthritis |
|  | MBP/MOG/PLP in Immune System Activation |
|  | Proteins Involved in Multiple Sclerosis |
|  | Proteins Involved in Endometriosis |
|  | Proteins Involved in Spontaneous Abortion |
|  | Proteins Involved in Age-Related Macular Degeneration |
|  | Cytokines Trigger Otitis Media |
|  | Proteins with Altered Expression in Asthma |
|  | Proteins Involved in Chronic Obstructive Pulmonary Disease |
|  | Proteins Involved in Pulmonary Emphysema |
|  | Proteins Involved in Glomerulonephritis |
|  | Proteins Involved in Pyelonephritis |
|  | Elevated Receptors -> Expression Targets in Adipose Tissue |
|  | Elevated Receptors -> Expression Targets in Bone and Joint |
|  | CD81 Expression Targets |
|  | CD86 -> ATF/CREB/CREBBP Expression Targets |
|  | CD86 -> STAT Expression Targets |
|  | CD86-> NFATC Expression Targets |
|  | HGF -> STAT Expression Targets |
|  | IL23A and IL17A Provoke Cancer-Associated Inflammation |
|  | Proteins with Altered Expression in Tumor-Promoting Inflammation |
|  | IFNG/IFNR Expression Targets |
|  | IL13 Expression Targets |
|  | IL21 Expression Targets |
|  | IL15 Expression Targets |
|  | IL12B Expression Targets |
|  | IL10 Expression Targets |
|  | IL9 Expression Targets |
|  | IL4 Expression Targets |
|  | CD Markers -> Expression Targets in Lymphoid System and Blood |
|  | GPCRs Family -> Expression Targets in Lymphoid System and Blood |
|  | Cell Adhesion Receptors -> Expression Targets in Lymphoid System and Blood |
|  | TNF Receptors -> Expression Targets in Lymphoid System and Blood |
|  | Receptor Tyrosine Kinase Family -> Expression Targets in Lymphoid System and Blood |
|  | Interleukin Receptors -> Expression Targets in Lymphoid System and Blood |
|  | CD40 -> Expression Targets in Thymus |
|  | Chemokine Receptor Family -> Expression Targets in Lymphoid System |
|  | DLL4 Expression Targets |
|  | TCR -> NF-kB Expression Targets |
|  | TCR -> NFAT Expression Targets |
|  | TCR -> STAT Expression Targets |
|  | TGFB1-TGFBR1/AP-1 Expression Targets |
|  | TGFB1-ACVRL1 Expression Targets |
|  | FASLG Expression Targets |
|  | TNF -> NF-kB Expression Targets |
|  | CD40LG -> STAT Expression Targets |
|  | CD40LG -> NF-kB/ELK/SRF -> CREB/NFATC Expression Targets |
|  | TNF -> STAT Expression Targets |
|  | IL1B -> PGE2 Expression Targets |
|  | IL1B Expression Targets |
|  | IL1A Expression Targets |
|  | PPAR Psoriasis |
|  | prarg negative regulators, ps-positive |
|  | pprarg neg, uknown targets, ps-positive |
|  | prarg neg,ukn expres targets, ps-positive |
|  | PPARG negative regulators |
|  | PPARG negative targets |
|  | pparg expr target\_regulators |
|  | Neighbors of disease exacerbation |
|  | Neighbors of tumor progression |
|  | Neighbors of cancer progression |
|  | Neighbors of inflammatory status |
|  | Neighbors of overall survival |
|  | Neighbors of disease severity |
|  | Neighbors of tumor size |
|  | Neighbors of lesion size |
|  | Neighbors of mortality |
|  | Neighbors of survival rate |
|  | Neighbors of infarct size |
|  | Neighbors of survival time |
|  | Neighbors of functional recovery |
|  | Neighbors of insulin sensitivity |
|  | Neighbors of bacterial load |
|  | Neighbors of cancer survival |
|  | Neighbors of graft survival |
|  | Neighbors of therapeutic efficacy |
|  | Neighbors of clinical stage |
|  | Neighbors of lethality |
|  | Neighbors of tumor regression |
|  | Neighbors of disease-free survival |
|  | Neighbors of tumor response |
|  | Neighbors of macrophage count |
|  | Neighbors of long-term survival |
|  | Neighbors of treatment outcome |
|  | Neighbors of bone mass |
|  | Neighbors of body weight |
|  | Neighbors of body weight gain |
|  | Neighbors of cardiovascular risk |
|  | Neighbors of microvessel density |
|  | Neighbors of tumor recurrence |
|  | Neighbors of tumor promotion |
|  | Neighbors of neutrophil count |
|  | Neighbors of glucose tolerance |
|  | Neighbors of morbidity |
|  | Neighbors of mortality rate |
|  | Neighbors of exhaustion |
|  | Neighbors of bone mineral density |
|  | Neighbors of adverse outcome |
|  | Neighbors of radioresistance |
|  | Neighbors of lesion area |
|  | Neighbors of cell density |
|  | Neighbors of progression-free survival |
|  | Neighbors of early mortality |
|  | Neighbors of viral load |
|  | Neighbors of blood pressure |
|  | Neighbors of leukocyte count |
|  | Neighbors of vascular reactivity |
|  | Neighbors of vascular leakage |
|  | Neighbors of vascular density |
|  | Neighbors of muscle mass |
|  | Neighbors of cancer risk |
|  | Neighbors of cancer incidence |
|  | Neighbors of systolic blood pressure |
|  | Neighbors of tumor resistance |
|  | Neighbors of blood glucose |
|  | Neighbors of median survival |
|  | Neighbors of fasting plasma glucose |
|  | Neighbors of osteoclast number |
|  | Neighbors of T-cell count |
|  | Neighbors of vascular tone |
|  | Neighbors of pregnancy outcome |
|  | Neighbors of functional status |
|  | Neighbors of gastric cancer risk |
|  | Neighbors of neurological outcome |
|  | Neighbors of infarct area |
|  | Neighbors of fertility |
|  | Neighbors of pathological response |
|  | Neighbors of lymphocyte count |
|  | Neighbors of skin thickness |
|  | Neighbors of comorbidity |
|  | Neighbors of bone volume |
|  | Neighbors of disease exacerbation |
|  | Neighbors of tumor progression |
|  | Neighbors of cancer progression |
|  | Neighbors of inflammatory status |
|  | Neighbors of overall survival |
|  | Neighbors of disease severity |
|  | Neighbors of tumor size |
|  | Neighbors of lesion size |
|  | Neighbors of mortality |
|  | Neighbors of survival rate |
|  | Neighbors of infarct size |
|  | Neighbors of survival time |
|  | Neighbors of functional recovery |
|  | Neighbors of insulin sensitivity |
|  | Neighbors of bacterial load |
|  | Neighbors of cancer survival |
|  | Neighbors of graft survival |
|  | Neighbors of therapeutic efficacy |
|  | Neighbors of clinical stage |
|  | Neighbors of lethality |
|  | Neighbors of tumor regression |
|  | Neighbors of disease-free survival |
|  | Neighbors of tumor response |
|  | Neighbors of macrophage count |
|  | Neighbors of long-term survival |
|  | Neighbors of treatment outcome |
|  | Neighbors of bone mass |
|  | Neighbors of body weight |
|  | Neighbors of body weight gain |
|  | Neighbors of cardiovascular risk |
|  | Neighbors of microvessel density |
|  | Neighbors of tumor recurrence |
|  | Neighbors of tumor promotion |
|  | Neighbors of neutrophil count |
|  | Neighbors of glucose tolerance |
|  | Neighbors of morbidity |
|  | Neighbors of mortality rate |
|  | Neighbors of exhaustion |
|  | Neighbors of bone mineral density |
|  | Neighbors of adverse outcome |
|  | Neighbors of radioresistance |
|  | Neighbors of lesion area |
|  | Neighbors of cell density |
|  | Neighbors of progression-free survival |
|  | Neighbors of early mortality |
|  | Neighbors of viral load |
|  | Neighbors of blood pressure |
|  | Neighbors of leukocyte count |
|  | Neighbors of vascular reactivity |
|  | Neighbors of vascular leakage |
|  | Neighbors of vascular density |
|  | Neighbors of muscle mass |
|  | Neighbors of cancer risk |
|  | Neighbors of cancer incidence |
|  | Neighbors of systolic blood pressure |
|  | Neighbors of tumor resistance |
|  | Neighbors of blood glucose |
|  | Neighbors of median survival |
|  | Neighbors of fasting plasma glucose |
|  | Neighbors of osteoclast number |
|  | Neighbors of T-cell count |
|  | Neighbors of vascular tone |
|  | Neighbors of pregnancy outcome |
|  | Neighbors of functional status |
|  | Neighbors of gastric cancer risk |
|  | Neighbors of neurological outcome |
|  | Neighbors of infarct area |
|  | Neighbors of fertility |
|  | Neighbors of pathological response |
|  | Neighbors of lymphocyte count |
|  | Neighbors of skin thickness |
|  | Neighbors of comorbidity |
|  | Neighbors of bone volume |
|  | Neighbors of cell development |
|  | Neighbors of immune response |
|  | Neighbors of cell population |
|  | Neighbors of T-cell development |
|  | Neighbors of inflammatory response |
|  | Neighbors of wound healing |
|  | Neighbors of angiogenesis |
|  | Neighbors of innate immune response |
|  | Neighbors of cell function |
|  | Neighbors of macrophage activation |
|  | Neighbors of cell formation |
|  | Neighbors of adaptive immune response |
|  | Neighbors of osteoclast development |
|  | Neighbors of epithelial to mesenchymal transition |
|  | Neighbors of immunity |
|  | Neighbors of cell proliferative response |
|  | Neighbors of chemotaxis |
|  | Neighbors of dendritic cell differentiation |
|  | Neighbors of cell survival |
|  | Neighbors of osteoclast differentiation |
|  | Neighbors of cell phenotype |
|  | Neighbors of T-cell proliferation |
|  | Neighbors of tissue remodeling |
|  | Neighbors of cell adhesion |
|  | Neighbors of T-cell activation |
|  | Neighbors of endothelial cell proliferation |
|  | Neighbors of fibrogenesis |
|  | Neighbors of tumor growth |
|  | Neighbors of dendritic cell development |
|  | Neighbors of tissue repair |
|  | Neighbors of cell count |
|  | Neighbors of cell infiltration |
|  | Neighbors of cell interaction |
|  | Neighbors of SMC proliferation |
|  | Neighbors of lumen formation |
|  | Neighbors of endothelial cell migration |
|  | Neighbors of adipogenesis |
|  | Neighbors of lipid storage |
|  | Neighbors of cell damage |
|  | Neighbors of pregnancy |
|  | Neighbors of monocyte differentiation |
|  | Neighbors of aging |
|  | Neighbors of cell motility |
|  | Neighbors of bone resorption |
|  | Neighbors of monocyte migration |
|  | Neighbors of fibroblast proliferation |
|  | Neighbors of adipocyte differentiation |
|  | Neighbors of macrophage migration |
|  | Neighbors of microglial activation |
|  | Neighbors of neutrophil recruitment |
|  | Neighbors of regeneration |
|  | Neighbors of cell migration |
|  | Neighbors of cancer growth |
|  | Neighbors of stem cell proliferation |
|  | Neighbors of smooth muscle cell migration |
|  | Neighbors of hepatic regeneration |
|  | Neighbors of transendothelial migration |
|  | Neighbors of phagocytosis |
|  | Neighbors of immune system activation |
|  | Neighbors of leukocyte migration |
|  | Neighbors of leukocyte recruitment |
|  | Neighbors of osteoclast formation |
|  | Neighbors of monocyte recruitment |
|  | Neighbors of cell invasion |
|  | Neighbors of heart function |
|  | Neighbors of hemopoiesis |
|  | Neighbors of bone remodeling |
|  | Neighbors of ossification |
|  | Neighbors of T-cell function |
|  | Neighbors of immune system function |
|  | Neighbors of lung development |
|  | Neighbors of autophagy |
|  | Neighbors of monocyte adhesion |
|  | Neighbors of transcription activation |
|  | Neighbors of senescence |
|  | Neighbors of T-cell response |
|  | Neighbors of tumor immunity |
|  | Neighbors of hepatocyte proliferation |
|  | Neighbors of cell transdifferentiation |
|  | Neighbors of neuroprotection |
|  | Neighbors of stem cell differentiation |
|  | Neighbors of neuronal death |
|  | Neighbors of cell growth |
|  | Neighbors of ROS generation |
|  | Neighbors of epithelial cell proliferation |
|  | Neighbors of first trimester pregnancy |
|  | Neighbors of cancer cell growth |
|  | Neighbors of sensitization |
|  | Neighbors of hemato-encephalic barrier |
|  | Neighbors of macrophage function |
|  | Neighbors of cellular immune response |
|  | Neighbors of cellular senescence |
|  | Neighbors of colony formation |
|  | Neighbors of macrophage differentiation |
|  | Neighbors of cell differentiation |
|  | Neighbors of keratinocyte proliferation |
|  | Neighbors of stem cell migration |
|  | Neighbors of life span |
|  | Neighbors of cell homeostasis |
|  | Neighbors of psoriasis |
|  | Neighbors of inflammatory disease |
|  | Neighbors of rheumatoid arthritis |
|  | Neighbors of atherosclerosis |
|  | Neighbors of fibrosis |
|  | Neighbors of colitis |
|  | Neighbors of arthritis |
|  | Neighbors of inflammatory bowel disease |
|  | Neighbors of injury |
|  | Neighbors of atherogenesis |
|  | Neighbors of vascular remodeling |
|  | Neighbors of metastasis |
|  | Neighbors of carcinogenesis |
|  | Neighbors of multiple sclerosis |
|  | Neighbors of chronic inflammation |
|  | Neighbors of inflammation |
|  | Neighbors of tumor microenvironment |
|  | Neighbors of pathological angiogenesis |
|  | Neighbors of obesity |
|  | Neighbors of vascular disease |
|  | Neighbors of diabetes mellitus |
|  | Neighbors of asthma |
|  | Neighbors of pneumonia |
|  | Neighbors of hyperplasia |
|  | Neighbors of osteoarthritis |
|  | Neighbors of liver fibrosis |
|  | Neighbors of chronic obstructive pulmonary disease |
|  | Neighbors of myocardial infarction |
|  | Neighbors of leukocyte infiltration |
|  | Neighbors of reperfusion injury |
|  | Neighbors of airway inflammation |
|  | Neighbors of hepatitis |
|  | Neighbors of insulin resistance |
|  | Neighbors of infection |
|  | Neighbors of vascular injury |
|  | Neighbors of renal injury |
|  | Neighbors of preeclampsia |
|  | Neighbors of macrophage infiltration |
|  | Neighbors of liver injury |
|  | Neighbors of neuroinflammation |
|  | Neighbors of sepsis |
|  | Neighbors of death |
|  | Neighbors of type 1 diabetes |
|  | Neighbors of encephalomyelitis |
|  | Neighbors of autoimmune disease |
|  | Neighbors of ischemic stroke |
|  | Neighbors of dermatitis |
|  | Neighbors of neutrophil infiltration |
|  | Neighbors of heart disease |
|  | Neighbors of diabetic nephropathy |
|  | Neighbors of lung injury |
|  | Neighbors of immunopathology |
|  | Neighbors of systemic sclerosis |
|  | Neighbors of hepatocellular carcinoma |
|  | Neighbors of autoimmunity |
|  | Neighbors of acute lung injury |
|  | Neighbors of gastric cancer |
|  | Neighbors of atherosclerotic plaque |
|  | Neighbors of experimental autoimmune encephalomyelitis |
|  | Neighbors of colorectal cancer |
|  | Neighbors of cardiac remodeling |
|  | Neighbors of breast cancer |
|  | Neighbors of ischemia |
|  | Neighbors of systemic lupus erythematosus |
|  | Neighbors of melanoma |
|  | Neighbors of collagen-induced arthritis |
|  | Neighbors of cartilage degeneration |
|  | Neighbors of interstitial fibrosis |
|  | Neighbors of neoplasm invasion |
|  | Neighbors of kidney disease |
|  | Neighbors of heart failure |
|  | Neighbors of lung disease |
|  | Neighbors of pulmonary fibrosis |
|  | Neighbors of cardiovascular disease |
|  | Neighbors of stroke |
|  | Neighbors of cancer |
|  | Neighbors of type 2 diabetes |
|  | Neighbors of neoplasm |
|  | Neighbors of endometriosis |
|  | Neighbors of vasculitis |
|  | Neighbors of atrophy |
|  | Neighbors of diet-induced obesity |
|  | Neighbors of respiratory hypersensitivity |
|  | Neighbors of myocardial fibrosis |
|  | Neighbors of synovitis |
|  | Neighbors of coronary artery disease |
|  | Neighbors of rupture |
|  | Neighbors of endothelial cell dysfunction |
|  | Neighbors of liver disease |
|  | Neighbors of prostate cancer |
|  | Neighbors of hypertrophy |
|  | Neighbors of osteolysis |
|  | Neighbors of ovarian cancer |
|  | Neighbors of pancreatic cancer |
|  | Neighbors of hypertension |
|  | Neighbors of intimal hyperplasia |
|  | Neighbors of allograft rejection |
|  | Neighbors of proteinuria |
|  | Neighbors of epithelial cell |
|  | Neighbors of keratinocyte |
|  | Neighbors of monocyte |
|  | Neighbors of endometrial stromal cell |
|  | Neighbors of alveolar macrophage |
|  | Neighbors of synoviocyte |
|  | Neighbors of macrophage |
|  | Neighbors of adipocyte |
|  | Neighbors of fibroblast |
|  | Neighbors of airway epithelial cell |
|  | Neighbors of PBMC |
|  | Neighbors of stromal cell |
|  | Neighbors of airway smooth muscle cell |
|  | Neighbors of osteoblast |
|  | Neighbors of pneumocyte |
|  | Neighbors of endometrium cell |
|  | Neighbors of microglia |
|  | Neighbors of trophoblast |
|  | Neighbors of intestine epithelium cell |
|  | Neighbors of mononuclear cell |
|  | Neighbors of hepatic stellate cell |
|  | Neighbors of hepatocyte |
|  | Neighbors of endothelial cell |
|  | Neighbors of dendritic cell |
|  | Neighbors of Kuppfer cell |
|  | Neighbors of proadipocyte |
|  | Neighbors of kidney tubule cell |
|  | Neighbors of inflammatory cell |
|  | Neighbors of bone marrow macrophage |
|  | Neighbors of peritoneal macrophage |
|  | Neighbors of osteoclast |
|  | Neighbors of neutrophil |
|  | Neighbors of smooth muscle myocyte |
|  | Neighbors of astrocyte |
|  | Neighbors of bone marrow derived dendritic cell |
|  | Neighbors of activated T-cell |
|  | Neighbors of mesothelial cell |
|  | Neighbors of immunocompetent cell |
|  | Neighbors of granulocyte |
|  | Neighbors of cancer-associated fibroblast |
|  | Neighbors of myeloid cell |
|  | Neighbors of mesenchymal stroma cell |
|  | Neighbors of corneal epithelial cell |
|  | Neighbors of stem cell |
|  | Neighbors of tumor infiltrating macrophage |
|  | Neighbors of B-cell |
|  | Neighbors of leukocyte |
|  | Neighbors of chondrocyte |
|  | Neighbors of insulin-secreting cell |
|  | Neighbors of mesenchymal cell |
|  | Neighbors of T-cell |
|  | Neighbors of M1 macrophage |
|  | Neighbors of mast cell |
|  | Neighbors of bone marrow stromal cell |
|  | Neighbors of spleen cell |
|  | Neighbors of neuroglia |
|  | Neighbors of aortic smooth muscle cell |
|  | Neighbors of hematopoietic cell |
|  | Neighbors of conventional dendritic cell |
|  | Neighbors of pericyte |
|  | Neighbors of antigen-presenting cell |
|  | Neighbors of bone marrow derived mast cell |
|  | Neighbors of phagocyte |
|  | Neighbors of kidney cell |
|  | Neighbors of lymphocyte |
|  | Neighbors of Langerhans cell |
|  | Neighbors of brain cell |
|  | Neighbors of M2 macrophage |
|  | Neighbors of peritoneum cell |
|  | Neighbors of lymphoid cell |
|  | Neighbors of retina cell |
|  | Neighbors of blood cell |
|  | Neighbors of skin cell |
|  | Neighbors of peripheral lymphocyte |
|  | Neighbors of activated B-cell |
|  | Neighbors of lymph node cell |
|  | Neighbors of lung |
|  | Neighbors of liver |
|  | Neighbors of kidney |
|  | Neighbors of skin |
|  | Neighbors of intestine |
|  | Neighbors of lymph node |
|  | Neighbors of colon |
|  | Neighbors of airway |
|  | Neighbors of heart |
|  | Neighbors of aorta |
|  | Neighbors of gastrointestinal tract |
|  | Neighbors of microvessel |
|  | Neighbors of bone |
|  | Neighbors of placenta |
|  | Neighbors of synovial membrane |
|  | Neighbors of uterus |
|  | Neighbors of esophagus |
|  | Neighbors of periodontium |
|  | Neighbors of retina |
|  | Neighbors of brain |
|  | Neighbors of ovary |
|  | Neighbors of breast |
|  | Neighbors of blood vessel wall |
|  | Neighbors of stomach |
|  | Neighbors of islets of Langerhans |
|  | Neighbors of joint |
|  | Neighbors of cardiovascular system |
|  | Neighbors of pancreas |
|  | Neighbors of prostate |
|  | Neighbors of central nervous system |
|  | Neighbors of kidney glomerulus |
|  | Neighbors of lymphatic system |
|  | Neighbors of colorectal region |
|  | Neighbors of decidua |
|  | Neighbors of immune system |
|  | Neighbors of spleen |
|  | Neighbors of peritoneum |
|  | Neighbors of alveolus |
|  | Neighbors of coronary artery |
|  | Neighbors of villus |
|  | Neighbors of bronchus |
|  | Neighbors of glands |
|  | Neighbors of nose |
|  | Neighbors of eye |
|  | Neighbors of lymph node of mesentery |
|  | Neighbors of vagina |
|  | Neighbors of thymus gland |
|  | Neighbors of brain blood vessel |
|  | Neighbors of myelin sheath |
|  | Neighbors of draining lymph node |
|  | Neighbors of nervous system |
|  | Neighbors of cervical lymph node group |
|  | Neighbors of salivary gland |
|  | Neighbors of small intestine |
|  | Neighbors of aggregated lymphoid follicle of small intestine |
|  | Neighbors of mediastinal lymph node group |
|  | Neighbors of hip region |
|  | Neighbors of reproductive system |
|  | Neighbors of NF-kB family |
|  | Neighbors of oxidized LDL |
|  | Neighbors of JNK |
|  | Neighbors of IL1 family |
|  | Neighbors of PPAR |
|  | Neighbors of Jun/Fos |
|  | Neighbors of NFKBI |
|  | Neighbors of PKC |
|  | Neighbors of PI3K |
|  | Neighbors of STAT family |
|  | Neighbors of mitogen-activated protein kinase |
|  | Neighbors of IFNAR ligand |
|  | Neighbors of transforming growth factor |
|  | Neighbors of TLR |
|  | Neighbors of cytokine |
|  | Neighbors of interferon |
|  | Neighbors of interleukin |
|  | Neighbors of JAK |
|  | Neighbors of MEK1/2 |
|  | Neighbors of Ras GTPase |
|  | Neighbors of ERK1/2 |
|  | Neighbors of PKA |
|  | Neighbors of ovalbumin |
|  | Neighbors of NF-AT family |
|  | Neighbors of beta adrenoceptor |
|  | Neighbors of chorionic gonadotropin |
|  | Neighbors of protein tyrosine kinase |
|  | Neighbors of NAD(P)H oxidase |
|  | Neighbors of IgG |
|  | Neighbors of SMAD subfamily |
|  | Neighbors of LXR |
|  | Neighbors of inflammatory cytokine |
|  | Neighbors of IL23 |
|  | Neighbors of T-cell receptor |
|  | Neighbors of histone deacetylase |
|  | Neighbors of IL1R |
|  | Neighbors of HIF-1 |
|  | Neighbors of GPCR |
|  | Neighbors of IL12 |
|  | Neighbors of Notch |
|  | Neighbors of heat shock protein 90 |
|  | Neighbors of lectin |
|  | Neighbors of fibrinogen |
|  | Neighbors of interleukin-35 |
|  | Neighbors of endothelin |
|  | Neighbors of NOS |
|  | Neighbors of superoxide dismutase |
|  | Neighbors of proteasome endopeptidase complex |
|  | Neighbors of class nuclear receptor with C4 zinc fingers |
|  | Neighbors of non-coding RNA |
|  | Neighbors of STAT5 |
|  | Neighbors of PP2A |
|  | Neighbors of retinoid-X receptor subfamily |
|  | Neighbors of immunoglobulin |
|  | Neighbors of PP2B |
|  | Neighbors of AHR subfamily |
|  | Neighbors of ITG |
|  | Neighbors of endostatins |
|  | Neighbors of CD3 complex |
|  | Neighbors of TLR1/2 heterodimer |
|  | Neighbors of TORC1 |
|  | Neighbors of RA receptor |
|  | Neighbors of histone |
|  | Neighbors of Rho kinase |
|  | Neighbors of neuropeptides |
|  | Neighbors of pancreatic elastase |
|  | Neighbors of histone acetyltransferase |
|  | Neighbors of phosphoinositide phospholipase C |
|  | Neighbors of deacetylase |
|  | Neighbors of TLR4/Myd88 |
|  | comon ps\_pos, pprarg\_neg targets |
|  | Model of PPARG signaling in psoriasis |
|  | PPARG negative regulators and targets |
|  | Model of PPARG related pathways in psoriasis (short version) |
|  | New Pathway (5) |
|  | New Pathway (3) |
|  | Overview. Diseases of the respiratory (chapter 9) |
|  | 1\_Differentiation of psoriatic T cells |
|  | 2\_Interleukin-17 and interleukin-22 signaling in psoriasis |
|  | 1\_Th2 cell response in asthma |
|  | 2\_3\_Eosinophilia and neutrophilia in asthma: Neutrophil chemotaxis and activation |
|  | 2\_Paneth cell dysfunction in Crohn's disease |
|  | 1\_Defects in response to pathogens in the gut promotes inflammation in Crohn's disease |
|  | 4\_1\_Polymorphisms associated with inflammatory bowel diseases |
|  | drug-target moltransport |
|  | Model of PPARG signaling in psoriais (tested) |
|  | before laser treatment |
|  | Differentiation of psoriatic T cells |
|  | Interleukin-17 and interleukin-22 signaling in psoriasis |
|  | Anti-psoriatic drugs influence PPARG signaling |
|  | PPARG signaling after laser treatment |

---

|  |  |
| --- | --- |
| MedScan ID | 3605 |
|  | 25465 |

---

|  |  |
| --- | --- |
| LocusLink ID | 3605 |
|  | 16171 |
|  | 301289 |

---

|  |  |
| --- | --- |
| Alias | cytotoxic T-lymphocyte-associated protein 8 |
|  | LOC63189 |
|  | Cytotoxic T lymphocyte-associated antigen VIII |
|  | cytotoxic T-lymphocyte-associated serine esterase VIII |
|  | IL-17A |
|  | cytotoxic T lymphocyte antigen 8 |
|  | Interleukin IL17 |
|  | OTTHUMP00000016597 |
|  | LOC301289 |
|  | IL-17As |
|  | cytotoxic T-lymphocyte-associated serine esterase 8 |
|  | interleukin 17 (cytotoxic T-lymphocyte-associated serine esterase 8) |
|  | Cytotoxic T lymphocyte-associated antigen 8 |
|  | interleukin IL17\_HUMAN |
|  | interleukin IL-17As |
|  | LOC94918 |
|  | CTLA8 |
|  | interleukin IL-17A |
|  | IL17 |
|  | interleukin 17 |
|  | interleukin 17a |
|  | IL17\_HUMAN |
|  | cytotoxic T lymphocyte protein 8 |
|  | IL-17 |
|  | CTLA-8 |
|  | cytotoxic T-lymphocyte-associated antigen 8 |
|  | interleukin-17A |
|  | hypothetical protein LOC301289 |

---

|  |  |
| --- | --- |
| GO ID | 0005125 |
|  | 0006915 |
|  | 0008219 |
|  | 0007267 |
|  | 0071347 |
|  | 0019221 |
|  | 0050832 |
|  | 0072537 |
|  | 0097530 |
|  | 0006955 |
|  | 0006954 |
|  | 0097400 |
|  | 1900017 |
|  | 0032747 |
|  | 2000778 |
|  | 0045672 |
|  | 0045944 |
|  | 0009897 |
|  | 0005576 |
|  | 0005615 |
|  | 0071385 |
|  | 0010940 |
|  | 0005737 |
|  | 0010469 |
|  | 0043200 |
|  | 0005126 |
|  | 0007166 |
|  | 0006486 |

---

|  |  |
| --- | --- |
| KEGG ID | hsa:3605 |
|  | mmu:16171 |
|  | rno:301289 |

---

|  |  |
| --- | --- |
| Organism | Homo sapiens {Organism urn:agi-taxid:9606} |
|  | Mus musculus {Organism urn:agi-taxid:10090} |
|  | Rattus norvegicus {Organism urn:agi-taxid:10116} |
|  | Homo sapiens |
|  | Mus musculus |
|  | Rattus norvegicus |

---

|  |  |
| --- | --- |
| Mouse chromosome position | 1 |

---

|  |  |
| --- | --- |
| OMIM ID | 603149 |

---

|  |  |
| --- | --- |
| Rat chromosome position | 9q13 |

---

|  |  |
| --- | --- |
| Hugo ID | 5981 |
|  | HGNC:5981 |

---

|  |  |
| --- | --- |
| Human chromosome position | 6p12.2 |
|  | 6p12 |

---

|  |  |
| --- | --- |
| Swiss-Prot Accession | Q16552 |
|  | Q16552.1 |
|  | Q544E6 |
|  | Q62386 |
|  | Q62386.1 |
|  | G3V7M4 |
|  | Q61453.1 |
|  | Q5T2P0 |
|  | Q60971 |
|  | Q61453 |
|  | Q6NZ94 |

---

|  |  |
| --- | --- |
| PIR ID | JC4628 |

---

|  |  |
| --- | --- |
| GenBank ID | NC\_000006 |
|  | NM\_002190 |
|  | NP\_002181 |
|  | NG\_033021 |
|  | AL391221 |
|  | AY460616 |
|  | AAR23263 |
|  | AY630567 |
|  | AAV41220 |
|  | CH471081 |
|  | EAX04362 |
|  | CS399132 |
|  | CAL44889 |
|  | GM832009 |
|  | CAV28580 |
|  | HC025843 |
|  | CBH19852 |
|  | JA104579 |
|  | CCA63959 |
|  | JA381713 |
|  | CCB07363 |
|  | BC066251 |
|  | AAH66251 |
|  | BC066252 |
|  | AAH66252 |
|  | BC066253 |
|  | AAH66253 |
|  | BC067503 |
|  | AAH67503 |
|  | BC067504 |
|  | AAH67504 |
|  | BC067505 |
|  | AAH67505 |
|  | HM535222 |
|  | ADJ56412 |
|  | U32659 |
|  | AAC50341 |
|  | Z58820 |
|  | CAA91233 |
|  | Q16552 |
|  | NC\_000067 |
|  | NM\_010552 |
|  | NP\_034682 |
|  | AC159614 |
|  | CH466536 |
|  | EDL14378 |
|  | GM832042 |
|  | CAV28591 |
|  | HC025841 |
|  | CBH19851 |
|  | U35108 |
|  | AAA93253 |
|  | AK040420 |
|  | BAC30590 |
|  | BC119303 |
|  | AAI19304 |
|  | BC119309 |
|  | AAI19310 |
|  | U43088 |
|  | AAB05222 |
|  | Q62386 |
|  | NC\_005108 |
|  | NM\_001106897 |
|  | NP\_001100367 |
|  | AC\_000077 |
|  | AABR07067028 |
|  | AAHX01058667 |
|  | CH473987 |
|  | EDM18652 |
|  | Q61453 |
|  | NC\_018917 |
|  | AMYH02013730 |
|  | HQ447533 |
|  | ADQ32019 |
|  | AC\_000023 |
|  | AAHY01004446 |
|  | AC\_000138 |
|  | ABBA01050560 |
|  | CAI16678 |
|  | NT\_007592 |
|  | NW\_001838981 |
|  | NW\_923073 |
|  | AC\_000049 |
|  | Q6NZ94 |
|  | NT\_039169 |
|  | NW\_001030649 |
|  | Q544E6 |
|  | NW\_047813 |
|  | NW\_001084880 |

---

|  |  |
| --- | --- |
| Swiss-Prot ID | IL17\_HUMAN |
|  | IL17\_MOUSE |
|  | Q544E6\_MOUSE |
|  | Q5T2P0\_HUMAN |

---

|  |  |
| --- | --- |
| Cell Localization | Secreted |
|  | Extracellular |

---

|  |  |
| --- | --- |
| Ensembl ID | ENSG00000112115 |
|  | ENSP00000344192.1 |
|  | ENST00000340057.1 |
|  | ENSMUSG00000025929 |
|  | ENSMUSP00000027061.4 |
|  | ENSMUST00000027061.4 |
|  | ENSRNOG00000012467 |
|  | ENSRNOP00000016664.3 |
|  | ENSRNOT00000016664.4 |
|  | ENSP00000344192 |
|  | ENST00000340057 |
|  | ENSMUSP00000027061 |
|  | ENSMUST00000027061 |
|  | ENSRNOP00000016664 |
|  | ENSRNOT00000016664 |

---

|  |  |
| --- | --- |
| MGI ID | MGI:107364 |
|  | 107364 |

---

|  |  |
| --- | --- |
| RGD ID | 2888 |
|  | 1587366 |

---

|  |  |
| --- | --- |
| Unigene ID | Mm.5419 |
|  | Rn.218513 |
|  | Hs.41724 |

---

|  |  |
| --- | --- |
| Homologene ID | 1651 |

---

|  |  |
| --- | --- |
| Shape | Rhomb |

---

|  |  |
| --- | --- |
| IPI ID | IPI00002898 |
|  | IPI00124807 |
|  | IPI00371779 |

---
