# Supplementary material for: Analysis of PPARγ Signaling Activity in Psoriasis
Source: Int J Mol Sci. 2021 Aug 10;22(16):8603. doi: 10.3390/ijms22168603 (PMC8395241; doi:10.3390/ijms22168603)
Supplement: Supplementary file 1 [file ijms-22-08603-s001.zip › Supplemental materials_Analysis of PPARg signaling activity in psoriasis/Pathway models/Models images and html files/Anti-psoriatic drugs influence PPARG signaling/69201.html]

vitamin A


# Small Molecule vitamin A

|  |  |
| --- | --- |
| URN | urn:agi-cas:68-26-8 |
| Total Entities | 3 |
| Connectivity | 3443 |
| Name | vitamin A |
| Class | Endogenous compound |
| Molecular Weight | 286.451600 |
| XLogP | 5.700000 |

---

|  |  |
| --- | --- |
| ChildConcepts | 3,4-didehydroretinol |
|  | retinyl methyl ether |
|  | retinyl-phenyl-ether |

---

|  |  |
| --- | --- |
| Pathway | Negative Acute Phase Proteins Synthesis |
|  | Visual Cycle in Retinal Cones |
|  | Visual Cycle in Retinal Rods |
|  | Vitamin A (Retinol) Metabolism and Visual Cycle |
|  | Androgens in Sebocyte Maturation |
|  | Steroidogenesis Impairement in Polycystic Ovary Syndrome |
|  | Polycystic Ovary Syndrome |
|  | Rod Visual Cycle Impairment in Retinitis pigmentosa |
|  | Retinoic Acid Role in Endometriosis |
|  | Dysgenesis of the Anterior Segment of the Eye in Glaucoma |
|  | 2\_Progesterone resistance stimulates endometriotic cell survival |
|  | Anti-psoriatic drugs influence PPARG signaling |

---

|  |  |
| --- | --- |
| MedScan ID | 1237078 |

---

|  |  |
| --- | --- |
| Alias | evitol zambeletti |
|  | vitamins A(1) |
|  | chivibit a |
|  | EINECS 234-328-2 |
|  | acrisine |
|  | fletase |
|  | Avita |
|  | mulsal a |
|  | Vitamin A1 |
|  | anti infective vitamin |
|  | bio tan |
|  | alphaline |
|  | all transretinol |
|  | dagravit a |
|  | anavit |
|  | Antixerophthalmisches Vitamin |
|  | Apexol |
|  | quotivit |
|  | preparato a |
|  | alfasir |
|  | panvita |
|  | Dohyfral A |
|  | halivitane |
|  | all-trans-3,7-Dimethyl-9-(2,6,6-trimethyl-1-cyclohexenyl)-2,4,6,8-nonatetraen-1-ol |
|  | vidoma |
|  | a mulsin |
|  | A-Mulsal |
|  | afilina |
|  | avitaminum kolin |
|  | Alcovit A |
|  | vitamin A |
|  | [8,9,19-13C]retinol |
|  | Apostavit |
|  | Vitaminum A |
|  | Alcohol 9,13-dimethyl-7-(1,1,5-trimethyl-6-cyclohexen-5-yl)-7,9,11,13-nonatetraen-15-ol |
|  | A vitamin |
|  | alfa monovite |
|  | alfavene |
|  | Oleovitamin A |
|  | 1341-18-0 |
|  | vitalen a |
|  | Vitavel-A |
|  | acrisina |
|  | Codoil, rosinol, rosin oil |
|  | [1-3H]retinol |
|  | cytobiase |
|  | idratene |
|  | vaconex |
|  | avibon theraplix |
|  | trans-retinol |
|  | avitadit |
|  | retinyl alcohol |
|  | trans-vitamin A alcohol |
|  | (all-E)-3,7-Dimethyl-9-(2,6,6-trimethyl-1-cyclohexen-1-yl)-2,4,6,8-nonatetraen-1-ol |
|  | Afaxin |
|  | alfaergine |
|  | all-trans-Vitamin A alcohol |
|  | A-Vitan |
|  | all-trans-Vitamin A |
|  | actifral a |
|  | Avibon |
|  | vitama |
|  | Vogan-nu |
|  | Homagenets Aoral |
|  | [(3)H]retinol |
|  | t-retinol |
|  | Agiolan |
|  | alfasole |
|  | Dofsol |
|  | Anatola |
|  | Prepalin |
|  | axerodine |
|  | multamine |
|  | axerophthol palmitate |
|  | nio a let |
|  | halivitan |
|  | HSDB 815 |
|  | Alphasterol |
|  | Bentavit A |
|  | vitadone |
|  | Veroftal |
|  | Vitpex |
|  | all-trans-Retinyl alcohol |
|  | ido a |
|  | 3,7-Dimethyl-9-(2,6,6-trimethyl-1-cyclohexen-1-yl)-2,4,6,8-nonate-traen-1-ol |
|  | b-Retinol |
|  | All-trans retinol |
|  | hydrosol |
|  | Rovimix A 500 |
|  | Vogan |
|  | all-trans-Vitamin A1 |
|  | [12C]retinol |
|  | Anatola A |
|  | homagenets aorl |
|  | vitamins A |
|  | vitadral |
|  | amulsal |
|  | vitamins-A |
|  | avitana |
|  | a vitadit |
|  | avitane |
|  | avipel |
|  | all-trans vitamin A1 alcohol |
|  | 11103-57-4 |
|  | alfamonovit |
|  | Vitamin A alcohol |
|  | Alphalin |
|  | Vitamin A1 alcohol |
|  | avital |
|  | 5979-23-7 |
|  | [(2)H(4)]retinol |
|  | Testavol S |
|  | ido a 50 |
|  | 3,7-Dimethyl-9-(2,6,6-trimethyl-1-cyclchexen-1-yl)-2,4,6,8-nonatetraen-1-ol |
|  | difvitamin a |
|  | avimin |
|  | 13123-33-6 |
|  | prepaline |
|  | Sehkraft A |
|  | axerodina |
|  | HI-A-Vita |
|  | avite |
|  | [H3]-retinol |
|  | Epiteliol |
|  | Axerol |
|  | Avita cream |
|  | arcavit A |
|  | avogine |
|  | adatone |
|  | Aquasynth |
|  | a vi pel |
|  | Hydrovit A |
|  | beta-Retinol |
|  | vitasan a |
|  | viatate |
|  | meditalfa |
|  | 1406-67-3 |
|  | [11,12(n)-3H]retinol |
|  | Retrovitamin A |
|  | avitina |
|  | avogina |
|  | CCRIS 5444 |
|  | a vit |
|  | 68-26-8 |
|  | Axerophtholum |
|  | Aoral |
|  | elageno a |
|  | Plivit A |
|  | Vogan-neu |
|  | alfatar |
|  | avitabiol |
|  | 3,7 dimethyl 9 (2,6,6 trimethyl 1 cyclohexen 1 yl) 2,4,6,8 nonatetraen 1 ol |
|  | alfavitina |
|  | avipur |
|  | viadenin |
|  | davitamon a |
|  | NSC 122759 |
|  | alfavitine |
|  | lord factor |
|  | alfamine |
|  | retinol alcohol |
|  | Vitamin A Oil |
|  | Vitamin A cryst |
|  | Antixerophthalmic vitamin |
|  | 3,7 dimethyl 9 (2,6,6 trimethyl 1 cyclohexenyl) 2,4,6,8 nonatetraen 1 ol |
|  | ro a vit |
|  | alfaergin |
|  | aterapion |
|  | primavit |
|  | amulsine |
|  | Retinol |
|  | Axerophthol |
|  | 17104-91-5 |
|  | EINECS 200-683-7 |
|  | afaxine |
|  | Ophthalamin |
|  | alfavena |
|  | All-Trans-Retinol(Vitamin A) |
|  | Disatabs Tabs |
|  | [13C]retinol |
|  | Avitol |
|  | Biosterol |
|  | vitalfa |
|  | Avita gel |
|  | wandervit a |
|  | amulvit |
|  | oleovit a |
|  | Wachstumsvitamin |
|  | asteril |
|  | envit a |
|  | amulsin |
|  | alfene |
|  | 53637-36-8 |
|  | vitaplex a |
|  | 21 CFR 182,5930 |
|  | alfa sir |
|  | [15-(14)C]retinol |
|  | [15-3H]retinol |
|  | axerophthylium |
|  | Chocola A |
|  | Vaflol |
|  | Testavol |
|  | inovitan a |
|  | avitil |
|  | Vitamin-A |
|  | alfamin |
|  | Vafol |
|  | alfasterolo |
|  | bentavite a |
|  | Agoncal |
|  | Retinolum |
|  | BRN 0403040 |
|  | Myvpack |
|  | [(14)C]retinol |
|  | xerophthol |
|  | all-trans-3,7-Dimethyl-9-(2,6,6-trimethyl-1-cyclohexen-1-yl)-2,4,6,8-nonatetraen-1-ol |
|  | gadeol |
|  | gadol |
|  | avitan |
|  | a mulsine |
|  | avoleum |
|  | Retinolo |
|  | avimine |
|  | vitapur a |
|  | Atars |
|  | ucemine a |
|  | biotan |
|  | Vitamine A |

---

|  |  |
| --- | --- |
| CAS ID | 68-26-8 |
|  | 13123-33-6 |
|  | 17104-91-5 |
|  | 5979-23-7 |
|  | 11103-57-4 |
|  | 1341-18-0 |
|  | 1406-67-3 |
|  | 53637-36-8 |

---

|  |  |
| --- | --- |
| Reaxys ID | 1913942 |
|  | 1913943 |
|  | 2054549 |
|  | 2054550 |
|  | 2054551 |
|  | 2054552 |
|  | 2054553 |
|  | 2418094 |
|  | 2506671 |
|  | 2506672 |
|  | 403040 |
|  | 4686508 |
|  | 4843007 |
|  | 4843008 |
|  | 5083756 |
|  | 5272383 |
|  | 6061208 |
|  | 6061209 |
|  | 8443692 |

---

|  |  |
| --- | --- |
| ChEBI ID | 17336 |

---

|  |  |
| --- | --- |
| HMDB ID | HMDB00305 |

---

|  |  |
| --- | --- |
| KEGG ID | C00473 |

---

|  |  |
| --- | --- |
| InChIKey | FPIPGXGPPPQFEQ-OVSJKPMPSA-N |

---

|  |  |
| --- | --- |
| Molecular Formula | C20H30O |

---

|  |  |
| --- | --- |
| PubChem SID | 134971209 |
|  | 134989732 |

---

|  |  |
| --- | --- |
| PubChem CID | 445354 |

---

|  |  |
| --- | --- |
| IUPAC Name | (2E,4E,6E,8E)-3,7-dimethyl-9-(2,6,6-trimethylcyclohexen-1-yl)nona-2,4,6,8-tetraen-1-ol |

---

|  |  |
| --- | --- |
| Rotatable Bond Count | 5 |

---
