# Supplementary material for: Analysis of PPARγ Signaling Activity in Psoriasis
Source: Int J Mol Sci. 2021 Aug 10;22(16):8603. doi: 10.3390/ijms22168603 (PMC8395241; doi:10.3390/ijms22168603)
Supplement: Supplementary file 1 [file ijms-22-08603-s001.zip › Supplemental materials_Analysis of PPARg signaling activity in psoriasis/Pathway models/Models images and html files/Anti-psoriatic drugs influence PPARG signaling/81725.html]

metformin


# Small Molecule metformin

|  |  |
| --- | --- |
| URN | urn:agi-cas:657-24-9 |
| Total Entities | 10 |
| Connectivity | 5017 |
| Name | metformin |
| Molecular Weight | 165.624580 |

---

|  |  |
| --- | --- |
| ChildConcepts | chlorhexidine |
|  | phenformin |
|  | guanylurea |
|  | proguanil |
|  | chlorproguanil |
|  | buformin |
|  | metformin glycinate |
|  | 1-ethyl-3-(3-dimethylaminopropyl) carbodiimide |
|  | 1-phenyl-3-trimethylaminopropyl carbodiimide |
|  | biguanidine |

---

|  |  |
| --- | --- |
| Pathway | Longevity Related Drugs |
|  | Hypoxia Inhibits mTOR Signaling |
|  | AMPK Related Catabolism Deceleration in Glucose Insufficiency |
|  | Anti-psoriatic drugs influence PPARG signaling |

---

|  |  |
| --- | --- |
| MedScan ID | 1249112 |

---

|  |  |
| --- | --- |
| Alias | metiguamide |
|  | Metiguanide monohydrochloride |
|  | 1,1-Dimethylbiguanide HCl |
|  | N,N-dimethyl-Imidodicarbonimidic diamide monohydrochloride |
|  | Diabex |
|  | euform retard |
|  | N1,N1-Dimethylbiguanide |
|  | n,n dimethylbiguanide retard |
|  | lyomet xr |
|  | Metformin-HCl |
|  | Metformina |
|  | Dianben |
|  | EINECS 211-517-8 |
|  | Fornidd |
|  | metaformin |
|  | Metforminum |
|  | Diamin |
|  | Glucophage |
|  | N,N-dimethyl-Imidodicarbonimidic diamide |
|  | n,n dimethylbiguanidine |
|  | Glupermin |
|  | Metolmin |
|  | vimetrol |
|  | Walaphage |
|  | [14C]metformin |
|  | Dimethylbiguanide hydrochloride |
|  | Diaformin |
|  | 1115-70-4 |
|  | N,N-Dimethyldiguanide |
|  | N,N-Dimethyldicarbonimido/ic diamide/imido |
|  | Siofor |
|  | apophage |
|  | n,n dimethylguanylguanidine |
|  | Glucophage-Mite |
|  | Diaphage |
|  | N,N-Dimethylbiguanide |
|  | Glifage |
|  | Mescorit |
|  | Metomin |
|  | glycomet |
|  | Diaberit |
|  | NSC 91485 |
|  | 1,1-Dimethylbiguanide |
|  | dimethyl-biguanide |
|  | 1,1-dimethyl-Biguanide HCl |
|  | Melbin |
|  | n,n-dimethylimidocarbonimidic diamide |
|  | Geamet |
|  | 657-24-9 |
|  | Flumamine |
|  | Glucophage 850 |
|  | N1,N1-Dimethylbiguanide hydrochloride |
|  | AI3-51264 |
|  | N,N-Dimethylbiguanide hydrochloride |
|  | espa-formin |
|  | metformine hcl |
|  | Glumetza |
|  | gludepatic |
|  | Gluconil |
|  | hipoglucin |
|  | Denkaform |
|  | N,N-Dimethylbiguanide HCl |
|  | (N,N-dimethylcarbamimidoyl)aminoformamidine HCl |
|  | Dabex |
|  | n' dimethylguanylguanide |
|  | glumeformin |
|  | Diformin |
|  | reglus-500 |
|  | glumet |
|  | Meglucon |
|  | lyomet (drug) |
|  | Glyformin |
|  | Metbay |
|  | Metforal |
|  | metphormin |
|  | Glibomet |
|  | Diabetmin |
|  | EINECS 214-230-6 |
|  | eraphage |
|  | glucostop |
|  | Metformin |
|  | Novo-Metformin |
|  | Apo-Metformin |
|  | Islotin |
|  | gliformin |
|  | maformin |
|  | Risidon |
|  | Dimethylguanylguanidine |
|  | LA 6023 |
|  | dybis |
|  | glucofage |
|  | Glyciphage |
|  | merckformin |
|  | (N,N-dimethylcarbamimidoyl)aminoformamidine hydrochloride |
|  | diaformina |
|  | glucophage sr |
|  | Gluformin |
|  | juformin |
|  | mellittin |
|  | Thiabet |
|  | Fortamet |
|  | (N,N-dimethylcarbamimidoyl)aminoformamidine |
|  | glymet |
|  | glucotika |
|  | glucoformin |
|  | N1,N1-Dimethylbiguanide HCl |
|  | Glucomet |
|  | glucophage xr |
|  | methformin |
|  | metformax |
|  | n',n' dimethyldiguanide |
|  | Fluamine |
|  | DMBG hydrochloride |
|  | Glucofago |
|  | Haurymellin hydrochloride |
|  | Dimethylbiguanidine |
|  | Haurymelin |
|  | deson |
|  | glibudon |
|  | glucomin |
|  | Metformine |
|  | Benofomin |
|  | Metformin hydrochloride |
|  | Glucophage Retard |
|  | Dimethylbiguanide |
|  | diabetformin |
|  | glufor |
|  | Riomet |
|  | glucophage xr extended release |
|  | melformin |
|  | Glucophage Forte |
|  | Dimethyldiguanide |
|  | D 15095 |
|  | diabetase |
|  | LA 6063 |
|  | diabetase s |
|  | Glycoran |
|  | biguanide metformin |
|  | 1,1-dimethyl-Biguanide hydrochloride |
|  | Diformin Retard |
|  | 1,1-dimethyl-Biguanide monohydrochloride |
|  | Obimet |
|  | lyomet sr |
|  | dimefor |
|  | DMBG HCl |
|  | Dimethylbiguanide HCl |
|  | Glucophage la 6023 |
|  | metfogamma |
|  | novomet |
|  | DMGG hydrochloride |
|  | diaformina lp |
|  | Metiguanide |
|  | n,n dimethyl biguanidine |
|  | 1,1-dimethyl-biguanide |
|  | Glucoform |
|  | (N,N-dimethylcarbamimidoyl)iminomethanediamine |
|  | LA-6063 |
|  | haurymellin |
|  | Diabefagos |
|  | n' dimethylguanylguanidine |
|  | diafat |
|  | glustress |
|  | Islotin retard |
|  | DMGG HCl |
|  | Haurymellin HCl |
|  | Glucomine |
|  | diametin |
|  | 1,1-Dimethylbiguanide hydrochloride |
|  | glucaminol |
|  | Glucohexal |
|  | glukophage |
|  | Diabetosan |
|  | Diabetex |
|  | Diabesin |
|  | Glucoliz |
|  | glafornil |
|  | HSDB 7080 |
|  | Miformin |
|  | Meguan |
|  | n,n-dimethylimidodicarbonimidic diamide |
|  | Glueophoge |
|  | glucoless |
|  | Siamformet |
|  | diabetmin retard |
|  | Biocos |
|  | i-max |
|  | Dextin |
|  | Gliguanid |
|  | n,n-dimethylimidodicarbonimidic diamide monohydrochloride |
|  | glupa |
|  | metfoliquid geriasan |
|  | Metformin monohydrochloride |

---

|  |  |
| --- | --- |
| CAS ID | 657-24-9 |
|  | 1115-70-4 |
|  | 144377-16-2 |
|  | 15537-72-1 |
|  | 56258-19-6 |

---

|  |  |
| --- | --- |
| Reaxys ID | 16657563 |
|  | 18242704 |
|  | 19872484 |
|  | 20891186 |
|  | 27075003 |
|  | 3626131 |
|  | 4858755 |
|  | 5790437 |
|  | 606492 |
|  | 6702901 |
|  | 6712036 |
|  | 8075255 |

---

|  |  |
| --- | --- |
| ChEBI ID | 6801 |

---

|  |  |
| --- | --- |
| PharmaPendium ID | Metformin Hydrochloride |

---

|  |  |
| --- | --- |
| HMDB ID | HMDB01921 |

---

|  |  |
| --- | --- |
| KEGG ID | C07151 |

---

|  |  |
| --- | --- |
| InChIKey | XZWYZXLIPXDOLR-UHFFFAOYSA-N |
|  | OETHQSJEHLVLGH-UHFFFAOYSA-N |

---

|  |  |
| --- | --- |
| Molecular Formula | C4H11N5 |
|  | C4H12ClN5 |

---

|  |  |
| --- | --- |
| PubChem SID | 134977327 |
|  | 134979449 |

---

|  |  |
| --- | --- |
| PubChem CID | 4091 |
|  | 14219 |

---

|  |  |
| --- | --- |
| XLogP-AA | -1.3 |

---

|  |  |
| --- | --- |
| IUPAC Name | 3-(diaminomethylene)-1,1-dimethyl-guanidine |
|  | 3-(diaminomethylene)-1,1-dimethyl-guanidine;hydrochloride |

---

|  |  |
| --- | --- |
| Rotatable Bond Count | 2 |

---
