# Supplementary material for: Analysis of PPARγ Signaling Activity in Psoriasis
Source: Int J Mol Sci. 2021 Aug 10;22(16):8603. doi: 10.3390/ijms22168603 (PMC8395241; doi:10.3390/ijms22168603)
Supplement: Supplementary file 1 [file ijms-22-08603-s001.zip › Supplemental materials_Analysis of PPARg signaling activity in psoriasis/Pathway models/Models images and html files/Anti-psoriatic drugs influence PPARG signaling/9120.html]

PPARG


# Protein PPARG

|  |  |
| --- | --- |
| URN | urn:agi-llid:5468 |
| Total Entities | 0 |
| Connectivity | 13252 |
| Name | PPARG |
| Description | peroxisome proliferator activated receptor gamma |
| Notes | This gene encodes a member of the peroxisome proliferator-activated receptor (PPAR) subfamily of nuclear receptors. PPARs form heterodimers with retinoid X receptors (RXRs) and these heterodimers regulate transcription of various genes. Three subtypes of PPARs are known: PPAR-alpha, PPAR-delta, and PPAR-gamma. The protein encoded by this gene is PPAR-gamma and is a regulator of adipocyte differentiation. Additionally, PPAR-gamma has been implicated in the pathology of numerous diseases including obesity, diabetes, atherosclerosis and cancer. Alternatively spliced transcript variants that encode different isoforms have been described. [provided by RefSeq, Jul 2008] |
| Primary Cell Localization | Nucleus |
| Class | Transcription factor |
| ObjectType | Protein |

---

|  |  |
| --- | --- |
| Pathway | Proteins Involved in Stem Cell Exhaustion in Aging |
|  | Oxidative Stress in Amyotrophic Lateral Sclerosis |
|  | Estrogen Deficiency in Female Obesity |
|  | Androgen Deficiency in Male Obesity |
|  | mTOR Signaling |
|  | A-cell (X-like): Ghrelin and Nesfatin-1 Synthesis |
|  | Thyroid Hormones Common Genomic Effects in Hyperthyroidism |
|  | Androgens in Adipocyte Activation |
|  | Lipids Enhance Apoptotic Cell Engulfment and Reduce Inflammation |
|  | Mast-Cell Activation via IgE Signaling |
|  | Neutrophil Recruitment and Priming |
|  | Gastric and Pancreatic Lipase Function |
|  | Adipokines Production by Adipocyte |
|  | Peroxisome Protein Import and Peroxisome Division |
|  | Proteins Involved in Pathogenesis of Arrhythmogenic Right Ventricular Cardiomyopathy |
|  | Proteins Involved in Atherosclerosis |
|  | Low-Density Lipoproteins and Chemokines in Atherosclerosis |
|  | Proteins Involved in Dilated Cardiomyopathy |
|  | Proteins Involved in Arterial Hypertension |
|  | Proteins Involved in Pulmonary Hypertension |
|  | BMP/TGF-beta Signaling Impairment in Pulmonary Hypertension |
|  | Proteins Involved in Myocarditis |
|  | Sebocyte Proliferation in Acne Vulgaris |
|  | Hyperseborrhea in Acne Vulgaris |
|  | Androgens in Sebocyte Maturation |
|  | Proteins Involved in Melanoma |
|  | Proteins Involved in Diabetes Mellitus Type 1 |
|  | Proteins Involved in Diabetes Mellitus Type 2 |
|  | Familial Partial Lipodystrophy Type 4 Progression (Hypothesis) |
|  | Familial Partial Lipodystrophy Type 3 Progression (Hypothesis) |
|  | Familial Partial Lipodystrophy Type 2 Progression (Hypothesis) |
|  | Berardinelli-Seip Syndrome Progression (Hypothesis) |
|  | Proteins Involved in Diabetic Nephropathy |
|  | Proteins Involved in Diabetic Neuropathy |
|  | Proteins Involved in Insulin Resistance |
|  | Adiponectin Synthesis Declines in Insulin Resistance |
|  | Proteins Involved in non-Alcoholic Fatty Liver Disease |
|  | Gluconeogenesis Impairment in non-Alcoholic Fatty Liver Disease |
|  | Lipid Metabolism Impairement in non-Alcoholic Fatty Liver Disease |
|  | beta-Cell Recovery in Diabetes Mellitus |
|  | Thyroid Hormones in Adipose Tissue Metabolism |
|  | Adipocyte Hypertrophy and Hyperplasia |
|  | Adipokines Production by Adipocyte Impaired in Obesity |
|  | Proteins Involved in Obesity |
|  | Proteins Involved in Colorectal Neoplasms |
|  | Metastatic Colorectal Cancer |
|  | Proteins Involved in Inflammatory Bowel Diseases |
|  | Proteins Involved in Helicobacter Infections |
|  | Epithelial Cell in the Innate Immune Response in Ulcerative Colitis |
|  | Leukocyte Migration toward the Endothelial Cell in Ulcerative Colitis |
|  | Proteins Involved in Ulcerative Colitis |
|  | Mast-Cell Activation in Asthma |
|  | Proteins Involved in Osteoarthritis |
|  | Proteins Involved in Polycystic Ovary Syndrome |
|  | Proteins Involved in Psoriatic Arthritis |
|  | Proteins Involved in Rheumatoid Arthritis |
|  | Proteins Involved in Periodontitis |
|  | PPARGC1A Repression in Huntington Disease |
|  | Proteins Involved in Huntington Disease |
|  | Proteins Involved in Lipodystrophy, Familial Partial |
|  | Lipodystrophy, Familial Partial |
|  | Proteins Involved in Osteopetrosis |
|  | Proteins Involved in Alzheimer's Disease |
|  | Proteins Involved in Glioma |
|  | Proteins Involved in Neuroblastoma |
|  | Proteins with Altered Expression in Amyotrophic Lateral Sclerosis |
|  | Proteins Involved in Endometriosis |
|  | Proteins Involved in Cholesteatoma |
|  | Fatty Acid Synthase (FASN) Signaling |
|  | Proteins Involved in Prostate Cancer |
|  | Proteins Involved in Chronic Obstructive Pulmonary Disease |
|  | Elevated Receptors -> Expression Targets in Adipose Tissue |
|  | CSF2 -> STAT Expression Targets |
|  | EGF -> CTNN Expression Targets |
|  | EGF -> AP-1/ATF Expression Targets |
|  | EGF -> CREB/CREBBP/ELK/SRF/MYC Expression Targets |
|  | EGF -> STAT Expression Targets |
|  | VEGFA Dependent Angiogenesis in Cancer |
|  | Cancer Cells Inhibit Adipocyte Differentiation |
|  | Insulin -> STAT Expression Targets |
|  | Insulin -> CEBPA/CTNNB/FOXA/FOXO Expression Targets |
|  | Insulin -> MEF/MYOD Expression Targets |
|  | Insulin -> ELK/SRF/HIF1A/MYC/SREBF Expression Targets |
|  | IL13 Expression Targets |
|  | IL11 Expression Targets |
|  | IL4 Expression Targets |
|  | Leptin -> STAT Expression Targets |
|  | LDLR -> Expression Targets in Lymphoid System and Blood |
|  | OR1A1 -> GNAS Signaling |
|  | OSM/OSMR Expression Targets |
|  | PRL/GHR -> STAT Expression Targets |
|  | PRL/GHR -> NF/kB/ELK/SRF/MYC Expression Targets |
|  | Lysophosphatidic Acid/LPARs Signaling |
|  | FOXO1 Signaling |
|  | TGFB1-ACVRL1 Expression Targets |
|  | TNF -> STAT Expression Targets |
|  | New Pathway (1) |
|  | PPAR Psoriasis |
|  | prarg negative regulators, ps-positive |
|  | pprarg neg, uknown targets, ps-positive |
|  | prarg neg,ukn expres targets, ps-positive |
|  | PPARG inhibits phenotypes assotiated with psoriasis |
|  | PPARG expressed in cells assotiated with psoriasis |
|  | efects selected |
|  | comon ps\_pos, pprarg\_neg targets |
|  | Model of PPARG signaling in psoriasis |
|  | PPARG negative regulators and targets |
|  | Model of PPARG related pathways in psoriasis (short version) |
|  | New Pathway (5) |
|  | PPARG-CCK4\_MME |
|  | PPARs and anxiety common targets |
|  | Amygdaloid expressed genes |
|  | Figure 3\_Mixed network of dexamethasone targets |
|  | Figure 3\_Mixed network of dexamethasone targets |
|  | 3\_Increased proliferation of sebocytes in acne vulgaris |
|  | 2\_Hyperseborrhea in acne vulgaris |
|  | 3\_Increased proliferation of pulmonary artery smooth muscle cells (PASMCs) in familial forms of pulmonary hypertension |
|  | 3\_Leukocyte migration toward the endothelial cells in the intestine microvasculature |
|  | 1\_Intestine epithelial cell dysfunction in ulcerative colitis |
|  | 4\_1\_Polymorphisms associated with inflammatory bowel diseases |
|  | 1\_Triglyceride storage in NAFLD |
|  | 3\_2\_Beta-cell proliferation and compensation: neogenesis/dedifferentiation |
|  | 4\_3\_Insulin resistance: FFA-related insulin resistance |
|  | 2\_2\_Effects of thyroid hormone deficiency: cell-specific effects of thyroid hormone action |
|  | drug-target pairs direct regulation |
|  | Model of PPARG signaling in psoriais (tested) |
|  | before laser treatment |
|  | mapk1 |
|  | Anti-psoriatic drugs influence PPARG signaling |
|  | PPARG signaling after laser treatment |

---

|  |  |
| --- | --- |
| Group | Genes with Mutations Associated with Atherosclerosis |
|  | Proteins Involved in Graves Ophthalmopathy |
|  | Genes with Mutations Associated with Obesity |
|  | Genes Associated with Ulcerative Colitis |
|  | Genes with Mutations Associated with Familial Partial Lipodystrophy |

---

|  |  |
| --- | --- |
| MedScan ID | 5468 |

---

|  |  |
| --- | --- |
| LocusLink ID | 5468 |
|  | 19016 |
|  | 25664 |

---

|  |  |
| --- | --- |
| Alias | peroxisomal proliferator activated receptor gamma 2 |
|  | OTTHUMP00000185030 |
|  | PPARc |
|  | PPAR-gamma |
|  | Peroxisome proliferator activated receptor gamma |
|  | PPARG |
|  | HUMPPARG |
|  | PPARG I |
|  | PPAR-C |
|  | peroxisome proliferative activated receptor gamma isoform I |
|  | peroxisomal proliferator (PP)-activator receptor gamma |
|  | peroxisomal proliferator activated receptor-c |
|  | peroxisome proliferators-activated receptor gamma |
|  | peroxisomal proliferator-activator receptor gamma |
|  | nuclear receptor subfamily 1 group C member 3 |
|  | OTTHUMP00000185032 |
|  | peroxisome proliferator-activated receptor gamma 1 |
|  | peroxisome proliferator activated receptor gamma 2 |
|  | PPAR-gamma2 |
|  | peroxisome proliferator (PP)-activator receptor gamma |
|  | OTTHUMP00000185036 |
|  | peroxisome proliferative activated receptor gamma isoform II |
|  | PPARG2 |
|  | NR1C3 |
|  | peroxisome proliferator (PP)-activated receptor gamma |
|  | peroxisome proliferative activated receptor gamma isoform 1 |
|  | OTTHUMP00000185033 |
|  | CIMT1 |
|  | PPARG1 |
|  | peroxisome proliferator-activator receptor gamma |
|  | peroxisome proliferative activated receptor gamma |
|  | peroxisomal proliferator (PP)-activated receptor gamma |
|  | peroxisome proliferators-activated receptor gamma 1 |
|  | peroxisome proliferative activated receptor gamma isoform 2 |
|  | OTTHUMP00000185037 |
|  | peroxisome proliferator-activated nuclear receptor gamma variant 1 |
|  | peroxisomal proliferator-activated receptor gamma 1 |
|  | peroxisomal proliferator activated receptor gamma |
|  | peroxisome proliferator activated receptor gamma 4 |
|  | PPAR-G |
|  | GLM1 |
|  | PPARgamma |
|  | peroxisome proliferator-activated receptor gamma |
|  | PPARgamma2 |
|  | peroxisome proliferator activator receptor, gamma |
|  | PPAR gamma |

---

|  |  |
| --- | --- |
| GO ID | 0050692 |
|  | 0003677 |
|  | 0003700 |
|  | 0000981 |
|  | 0001227 |
|  | 0070888 |
|  | 0050693 |
|  | 0000977 |
|  | 0001103 |
|  | 0033613 |
|  | 0051393 |
|  | 0050544 |
|  | 0003682 |
|  | 0003690 |
|  | 0008144 |
|  | 0019899 |
|  | 0030331 |
|  | 0005504 |
|  | 0008289 |
|  | 0004879 |
|  | 0030374 |
|  | 0042277 |
|  | 0004955 |
|  | 0008022 |
|  | 0046982 |
|  | 0019903 |
|  | 0043621 |
|  | 0046965 |
|  | 0043565 |
|  | 0038023 |
|  | 0003707 |
|  | 0008134 |
|  | 0044212 |
|  | 0000976 |
|  | 0008270 |
|  | 0007186 |
|  | 0006919 |
|  | 0031100 |
|  | 0030154 |
|  | 0045165 |
|  | 0048469 |
|  | 0071455 |
|  | 0032869 |
|  | 0071404 |
|  | 0071380 |
|  | 0071300 |
|  | 0071306 |
|  | 0030855 |
|  | 0006631 |
|  | 0019395 |
|  | 0042593 |
|  | 0007507 |
|  | 0009755 |
|  | 0045087 |
|  | 0055088 |
|  | 0006629 |
|  | 0042953 |
|  | 0015909 |
|  | 0045713 |
|  | 0010742 |
|  | 0030224 |
|  | 0007275 |
|  | 0002674 |
|  | 0016525 |
|  | 0043537 |
|  | 0030308 |
|  | 0010887 |
|  | 0032966 |
|  | 0060965 |
|  | 0060336 |
|  | 0010745 |
|  | 2000230 |
|  | 0010871 |
|  | 0010891 |
|  | 0048662 |
|  | 0051974 |
|  | 0000122 |
|  | 0045892 |
|  | 1905563 |
|  | 1904706 |
|  | 0035357 |
|  | 0001890 |
|  | 0043388 |
|  | 0051091 |
|  | 0045600 |
|  | 0046321 |
|  | 0048714 |
|  | 0060100 |
|  | 0045944 |
|  | 0045893 |
|  | 1905461 |
|  | 0061614 |
|  | 0008217 |
|  | 0060694 |
|  | 0042752 |
|  | 0019216 |
|  | 0006357 |
|  | 0060850 |
|  | 0031000 |
|  | 0009409 |
|  | 0043627 |
|  | 0035902 |
|  | 0033993 |
|  | 0009612 |
|  | 1901558 |
|  | 0007584 |
|  | 0032526 |
|  | 0042594 |
|  | 0033189 |
|  | 0048511 |
|  | 0007165 |
|  | 0043401 |
|  | 0006367 |
|  | 0050872 |
|  | 0090575 |
|  | 0005829 |
|  | 0043231 |
|  | 0005654 |
|  | 0005634 |
|  | 0048471 |
|  | 0032991 |
|  | 0001228 |
|  | 0001012 |
|  | 0050699 |
|  | 0046872 |
|  | 0050873 |
|  | 0071285 |
|  | 0071407 |
|  | 0002024 |
|  | 0045444 |
|  | 0006954 |
|  | 0008285 |
|  | 1900077 |
|  | 0001818 |
|  | 1903979 |
|  | 0150079 |
|  | 0090278 |
|  | 0043065 |
|  | 0045598 |
|  | 0010468 |
|  | 0006355 |
|  | 0002021 |
|  | 0032094 |
|  | 0009416 |
|  | 0005737 |
|  | 0071379 |
|  | 0010629 |
|  | 0010875 |
|  | 0032385 |
|  | 0031394 |
|  | 0050714 |
|  | 1903076 |
|  | 0042493 |
|  | 0014070 |
|  | 0010033 |
|  | 0045923 |
|  | 0071396 |
|  | 0050728 |
|  | 0098531 |
|  | 0006351 |
|  | 0031667 |
|  | 0001076 |
|  | 0001078 |
|  | 0001046 |
|  | 0030522 |
|  | 0010467 |
|  | 0036270 |
|  | 0055098 |
|  | 0005794 |
|  | 0006366 |
|  | 0006917 |
|  | 0032717 |
|  | 0010843 |
|  | 0016566 |
|  | 0016563 |
|  | 0016564 |
|  | 0010553 |
|  | 0010552 |
|  | 0034339 |
|  | 0004872 |
|  | 0017053 |

---

|  |  |
| --- | --- |
| KEGG ID | hsa:5468 |
|  | mmu:19016 |
|  | rno:25664 |

---

|  |  |
| --- | --- |
| Organism | Homo sapiens {Organism urn:agi-taxid:9606} |
|  | Mus musculus {Organism urn:agi-taxid:10090} |
|  | Rattus norvegicus {Organism urn:agi-taxid:10116} |
|  | Homo sapiens |
|  | Mus musculus |
|  | Rattus norvegicus |

---

|  |  |
| --- | --- |
| Mouse chromosome position | 6 53.41 cM |
|  | 6 52.7 cM |

---

|  |  |
| --- | --- |
| OMIM ID | 601487 |
|  | 609338 |
|  | 125853 |
|  | 604367 |
|  | 601665 |
|  | 137800 |
|  | 151660 |

---

|  |  |
| --- | --- |
| Rat chromosome position | 4q42 |

---

|  |  |
| --- | --- |
| Hugo ID | 9236 |
|  | HGNC:9236 |

---

|  |  |
| --- | --- |
| Human chromosome position | 3p25.2 |
|  | 3p25 |

---

|  |  |
| --- | --- |
| Swiss-Prot Accession | E9PFX5 |
|  | Q4W448 |
|  | D2KUA6 |
|  | P37231 |
|  | P37231.3 |
|  | M1VPI1 |
|  | P37238 |
|  | Q6GU14 |
|  | P37238.3 |
|  | O88275 |
|  | O88275.2 |
|  | A8K3G6 |
|  | B5BUA1 |
|  | O00684 |
|  | O00710 |
|  | O14515 |
|  | Q0QJH8 |
|  | Q15178 |
|  | Q15179 |
|  | Q15180 |
|  | Q15832 |
|  | Q86U60 |
|  | Q96J12 |
|  | Q9QWG0 |
|  | Q9R197 |
|  | Q4FJR2 |
|  | Q4W4C6 |
|  | Q4W4C7 |
|  | Q53EW1 |
|  | Q6L9M1 |
|  | Q86WD1 |
|  | Q9UEF6 |
|  | Q6TQE4 |

---

|  |  |
| --- | --- |
| PIR ID | JC4859 |
|  | PC4290 |
|  | PC4429 |
|  | A54101 |

---

|  |  |
| --- | --- |
| GenBank ID | NC\_000003 |
|  | NM\_001354666 |
|  | NP\_001341595 |
|  | NM\_138712 |
|  | NP\_619726 |
|  | NM\_001354669 |
|  | NP\_001341598 |
|  | NM\_005037 |
|  | NP\_005028 |
|  | NM\_138711 |
|  | NP\_619725 |
|  | NM\_001354667 |
|  | NP\_001341596 |
|  | NM\_001354670 |
|  | NP\_001341599 |
|  | NM\_001330615 |
|  | NP\_001317544 |
|  | NM\_001354668 |
|  | NP\_001341597 |
|  | NM\_015869 |
|  | NP\_056953 |
|  | NG\_011749 |
|  | AB005520 |
|  | BAA23353 |
|  | AB005526 |
|  | BAA23354 |
|  | AC090947 |
|  | AC091492 |
|  | AC093174 |
|  | AF012873 |
|  | AF012874 |
|  | AF310249 |
|  | AAK17925 |
|  | AF548352 |
|  | AY157024 |
|  | AAN38992 |
|  | CH471055 |
|  | EAW64123 |
|  | EAW64124 |
|  | EAW64125 |
|  | HI519538 |
|  | CBX54350 |
|  | KF457590 |
|  | KY547832 |
|  | ARH59648 |
|  | KY547833 |
|  | ARH59649 |
|  | KY547834 |
|  | ARH59650 |
|  | KY547835 |
|  | ARH59651 |
|  | KY547836 |
|  | ARH59652 |
|  | AB097931 |
|  | BAD20647 |
|  | AB107271 |
|  | BAD34540 |
|  | AB307692 |
|  | BAH02283 |
|  | AB451337 |
|  | BAG70151 |
|  | AB451486 |
|  | BAG70300 |
|  | AB472042 |
|  | BAI63629 |
|  | AB565476 |
|  | BAM71699 |
|  | AJ563369 |
|  | CAD91388 |
|  | AJ563370 |
|  | CAD91389 |
|  | AJ698135 |
|  | CAG29019 |
|  | AK027107 |
|  | AK123253 |
|  | AK223528 |
|  | BAD97248 |
|  | AK290581 |
|  | BAF83270 |
|  | AL523434 |
|  | BC006811 |
|  | AAH06811 |
|  | BG323933 |
|  | BI820841 |
|  | BM923992 |
|  | BQ003677 |
|  | BQ925271 |
|  | BT007281 |
|  | AAP35945 |
|  | D83233 |
|  | BAA18949 |
|  | DQ356894 |
|  | ABC97372 |
|  | HQ692866 |
|  | ADZ17377 |
|  | KU178267 |
|  | ALQ33725 |
|  | KU178268 |
|  | ALQ33726 |
|  | LS999978 |
|  | VAX75249 |
|  | LS999979 |
|  | VAX75250 |
|  | LS999980 |
|  | VAX75251 |
|  | U63415 |
|  | AAB04028 |
|  | U79012 |
|  | AAC51248 |
|  | X90563 |
|  | CAA62152 |
|  | CAA62153 |
|  | P37231 |
|  | NC\_000072 |
|  | XM\_006505743 |
|  | XP\_006505806 |
|  | NM\_001127330 |
|  | NP\_001120802 |
|  | NM\_001308352 |
|  | NP\_001295281 |
|  | XM\_006505737 |
|  | XP\_006505800 |
|  | XM\_006505739 |
|  | XP\_006505802 |
|  | XM\_017321455 |
|  | XP\_017176944 |
|  | XM\_017321456 |
|  | XP\_017176945 |
|  | XM\_006505738 |
|  | XP\_006505801 |
|  | NM\_001308354 |
|  | NP\_001295283 |
|  | XM\_011241252 |
|  | XP\_011239554 |
|  | XR\_001785108 |
|  | NM\_011146 |
|  | NP\_035276 |
|  | AB256529 |
|  | BAF80899 |
|  | AB256530 |
|  | BAF80900 |
|  | AC153828 |
|  | AC171970 |
|  | AH012542 |
|  | AH012543 |
|  | AH012840 |
|  | AH013272 |
|  | AH013273 |
|  | AH013274 |
|  | AAQ89994 |
|  | AY236530 |
|  | AY236531 |
|  | AY243584 |
|  | AY389534 |
|  | AY389535 |
|  | AY389536 |
|  | CH466523 |
|  | EDK99525 |
|  | EF062477 |
|  | ABK39949 |
|  | EF062478 |
|  | EF062479 |
|  | ABK39950 |
|  | AB644275 |
|  | BAM95277 |
|  | AY208183 |
|  | AAO45097 |
|  | AY208184 |
|  | AAO45098 |
|  | AY243585 |
|  | AAP42200 |
|  | BC021798 |
|  | AAH21798 |
|  | CJ043379 |
|  | CN701591 |
|  | CT010340 |
|  | CAJ18548 |
|  | EF062476 |
|  | ABK39948 |
|  | GQ868647 |
|  | ACX47078 |
|  | GQ868648 |
|  | ACX47079 |
|  | GQ868649 |
|  | ACX47080 |
|  | GQ868650 |
|  | ACX47081 |
|  | U01664 |
|  | AAA62110 |
|  | U01841 |
|  | AAC52134 |
|  | U09138 |
|  | AAA62277 |
|  | U10374 |
|  | AAA19971 |
|  | P37238 |
|  | NC\_005103 |
|  | XM\_006237009 |
|  | XP\_006237071 |
|  | NM\_001145367 |
|  | NP\_001138839 |
|  | NM\_001145366 |
|  | NP\_001138838 |
|  | NM\_013124 |
|  | NP\_037256 |
|  | AC\_000072 |
|  | AAHX01032447 |
|  | AAHX01032448 |
|  | AAHX01032449 |
|  | AAHX01032450 |
|  | AAHX01032451 |
|  | AC094445 |
|  | AC136055 |
|  | CH473964 |
|  | EDM02154 |
|  | EDM02155 |
|  | EDM02156 |
|  | AB011365 |
|  | BAA32540 |
|  | AB019561 |
|  | BAA36485 |
|  | AF156665 |
|  | AAD40118 |
|  | AF156666 |
|  | AAD40119 |
|  | AF246457 |
|  | AAF63385 |
|  | AF246458 |
|  | AAF63386 |
|  | Y12882 |
|  | CAA73382 |
|  | O88275 |
|  | AY243582 |
|  | AY243583 |
|  | XP\_011532144 |
|  | XP\_011532145 |
|  | XP\_011532146 |
|  | XP\_024309374 |
|  | XP\_024309373 |
|  | XP\_024309372 |
|  | AY389545 |
|  | AY389543 |
|  | AY389541 |
|  | AY389540 |
|  | AY389529 |
|  | AY389538 |
|  | AY389539 |
|  | AY389537 |
|  | XM\_011533842 |
|  | XM\_011533843 |
|  | XM\_011533844 |
|  | XM\_024453606 |
|  | XM\_024453604 |
|  | XM\_024453605 |
|  | DQ891108 |
|  | AY389528 |
|  | XM\_006713208 |
|  | DQ894289 |
|  | XP\_011532142 |
|  | XP\_011532143 |
|  | AC\_000028 |
|  | XP\_006713271 |
|  | ABM82034 |
|  | XP\_006505808 |
|  | XP\_006505807 |
|  | XP\_006505804 |
|  | XP\_006505803 |
|  | XP\_006505805 |
|  | NC\_018914 |
|  | AY389544 |
|  | AY389542 |
|  | AAQ89993 |
|  | AAQ89992 |
|  | AY389532 |
|  | ABM85215 |
|  | AY389533 |
|  | AY389530 |
|  | AY389531 |
|  | AMYH02006292 |
|  | AMYH02006290 |
|  | AMYH02006291 |
|  | XM\_011241253 |
|  | AY236532 |
|  | AY236534 |
|  | AY236533 |
|  | AY236536 |
|  | AY236535 |
|  | AY236537 |
|  | XM\_006505742 |
|  | XM\_006505741 |
|  | XM\_006505740 |
|  | XP\_011239555 |
|  | XM\_006505745 |
|  | XM\_006505744 |
|  | AC\_000135 |
|  | ABBA01025204 |
|  | XM\_011533841 |
|  | XM\_011533840 |
|  | AAHY01056756 |
|  | AAHY01056758 |
|  | AAHY01056757 |
|  | AABR06032979 |
|  | AABR06032980 |
|  | AABR06032981 |
|  | NT\_022517 |
|  | NW\_921651 |
|  | NW\_001838877 |
|  | AC\_000046 |
|  | CR599329 |
|  | CR605352 |
|  | CR609113 |
|  | CR609454 |
|  | CR622226 |
|  | Q0QJH8 |
|  | Q4W448 |
|  | Q4W4C6 |
|  | Q4W4C7 |
|  | Q53EW1 |
|  | Q6L9M1 |
|  | Q86WD1 |
|  | Q9UEF6 |
|  | NT\_039353 |
|  | NW\_001030811 |
|  | Q4FJR2 |
|  | Q6GU14 |
|  | Q6TQE4 |
|  | NW\_047696 |
|  | NW\_001084832 |

---

|  |  |
| --- | --- |
| Swiss-Prot ID | PPARG\_HUMAN |
|  | PPARG\_MOUSE |
|  | PPARG\_RAT |
|  | Q6GU14\_MOUSE |
|  | Q4FJR2\_MOUSE |
|  | D2KUA6\_HUMAN |

---

|  |  |
| --- | --- |
| Cell Localization | Nucleus |
|  | Cytoplasm |

---

|  |  |
| --- | --- |
| Ensembl ID | ENSG00000132170 |
|  | ENSP00000380196.2 |
|  | ENST00000397000.6 |
|  | ENSP00000380205.3 |
|  | ENST00000397010.7 |
|  | ENSP00000499004.1 |
|  | ENST00000651826.1 |
|  | ENSP00000287820.6 |
|  | ENST00000287820.10 |
|  | ENSP00000380207.2 |
|  | ENST00000397012.7 |
|  | ENSP00000498354.1 |
|  | ENST00000650761.1 |
|  | ENSMUSG00000000440 |
|  | ENSMUSP00000131962.1 |
|  | ENSMUST00000171644.7 |
|  | ENSMUSP00000145525.1 |
|  | ENSMUST00000203732.2 |
|  | ENSMUSP00000000450.3 |
|  | ENSMUST00000000450.4 |
|  | ENSRNOG00000008839 |
|  | ENSRNOP00000045012.3 |
|  | ENSRNOT00000051858.5 |
|  | ENSRNOP00000073235.1 |
|  | ENSRNOT00000082969.1 |
|  | ENSRNOP00000012137.3 |
|  | ENSRNOT00000012137.5 |
|  | ENSP00000380196.1 |
|  | ENSP00000312472.6 |
|  | ENST00000397000.5 |
|  | ENSP00000380205.2 |
|  | ENST00000397015.6 |
|  | ENST00000309576.10 |
|  | ENST00000643197.1 |
|  | ENSP00000380210.2 |
|  | ENSP00000495840.1 |
|  | ENST00000397010.6 |
|  | ENST00000397012.6 |
|  | ENSRNOP00000045012 |
|  | ENST00000287820 |
|  | ENSMUSP00000000450 |
|  | ENSP00000380196 |
|  | ENST00000397000 |
|  | ENSP00000312472 |
|  | ENSRNOT00000051858 |
|  | ENSMUST00000203732 |
|  | ENSRNOT00000012137 |
|  | ENSRNOT00000082969 |
|  | ENSP00000380205 |
|  | ENSMUST00000000450 |
|  | ENST00000397015 |
|  | ENSP00000287820 |
|  | ENST00000397012 |
|  | ENST00000397010 |
|  | ENSP00000380207 |
|  | ENST00000309576 |
|  | ENSRNOP00000073235 |
|  | ENSMUSP00000131962 |
|  | ENSMUST00000171644 |
|  | ENSRNOP00000012137 |
|  | ENSMUSP00000145525 |
|  | ENSP00000380210 |

---

|  |  |
| --- | --- |
| MGI ID | MGI:97747 |
|  | 97747 |

---

|  |  |
| --- | --- |
| RGD ID | 3371 |

---

|  |  |
| --- | --- |
| Unigene ID | Rn.23443 |
|  | Mm.3020 |
|  | Hs.162646 |
|  | Hs.655798 |

---

|  |  |
| --- | --- |
| Homologene ID | 7899 |

---

|  |  |
| --- | --- |
| Shape | Stick-vertex |

---

|  |  |
| --- | --- |
| IPI ID | IPI00020897 |
|  | IPI00853091 |
|  | IPI00926009 |
|  | IPI00207539 |
|  | IPI00231890 |
|  | IPI00125193 |
|  | IPI00266075 |
|  | IPI00913931 |
|  | IPI00797909 |
|  | IPI00852773 |
|  | IPI00791321 |
|  | IPI00853034 |
|  | IPI00853274 |
|  | IPI00896034 |

---
