# Supplementary material for: Analysis of PPARγ Signaling Activity in Psoriasis
Source: Int J Mol Sci. 2021 Aug 10;22(16):8603. doi: 10.3390/ijms22168603 (PMC8395241; doi:10.3390/ijms22168603)
Supplement: Supplementary file 1 [file ijms-22-08603-s001.zip › Supplemental materials_Analysis of PPARg signaling activity in psoriasis/Pathway models/Models images and html files/Anti-psoriatic drugs influence PPARG signaling/92660.html]

trichostatin A


# Small Molecule trichostatin A

|  |  |
| --- | --- |
| URN | urn:agi-cas:58880-19-6 |
| Total Entities | 1 |
| Connectivity | 3169 |
| Name | trichostatin A |
| Molecular Weight | 302.373000 |
| XLogP | 2.355000 |
| ObjectType | Small Molecule |

---

|  |  |
| --- | --- |
| ChildConcepts | trichostatin C |

---

|  |  |
| --- | --- |
| Pathway | Histone Acetylation |
|  | Possible Interplay between SOX11, CCND1 and EZH2 in Mantle Cell Lymphoma |
|  | Anti-psoriatic drugs influence PPARG signaling |

---

|  |  |
| --- | --- |
| MedScan ID | 1270319 |

---

|  |  |
| --- | --- |
| Alias | GR-309 |
|  | 4,6-Dimethyl-7-[p-dimethylaminophenyl]-7-oxahepta-2,4-dienohydroxamic Acid |
|  | (R-(E,E))-7-(4-(Dimethylamino)phenyl)-N-hydroxy-4,6-dimethyl-7-oxo-2,4-heptadienamide |
|  | (2E,4E,6R)-7-(4-(dimethylamino)phenyl)-N-hydroxy-4,6-dimethyl-7-oxo-2,4-Heptadienamide |
|  | 7-(4-(dimethylamino)phenyl)-N-hydroxy- 4,6-dimethyl-7-oxo-2,4-heptadienamide |
|  | TSA antibioitc |
|  | 7-(4-dimethylaminophenyl)-N-hydroxy-4,6-dimethyl-7-oxo-hepta-2,4-dienamide |
|  | Trichostatin A from Streptomyces sp. |
|  | A 300 |
|  | [R-(E,E)]-7-[4-(Dimethylamino)phenyl]-N-hydroxy-4,6-dimethyl-7-oxo-2,4-heptadienamide |
|  | trichostatin A |
|  | 58880-19-6 |
|  | 4,6-Dimethyl-7-(p-dimethylaminophenyl)-7-oxahepta-2,4-dienohydroxamic Acid |
|  | tricostatin A |

---

|  |  |
| --- | --- |
| CAS ID | 58880-19-6 |

---

|  |  |
| --- | --- |
| Reaxys ID | 2391788 |
|  | 5291761 |
|  | 5291762 |

---

|  |  |
| --- | --- |
| ChEBI ID | 46024 |

---

|  |  |
| --- | --- |
| InChIKey | RTKIYFITIVXBLE-QEQCGCAPSA-N |
|  | RTKIYFITIVXBLE-WKWSCTOISA-N |

---

|  |  |
| --- | --- |
| Molecular Formula | C17H22N2O3 |

---

|  |  |
| --- | --- |
| PubChem SID | 135060341 |

---

|  |  |
| --- | --- |
| PubChem CID | 444732 |

---

|  |  |
| --- | --- |
| XLogP-AA | 2.7 |

---

|  |  |
| --- | --- |
| IUPAC Name | (2E,4E,6R)-7-[4-(dimethylamino)phenyl]-7-keto-4,6-dimethyl-hepta-2,4-dienehydroxamic acid |

---

|  |  |
| --- | --- |
| Rotatable Bond Count | 6 |

---
