# Supplementary material for: Analysis of PPARγ Signaling Activity in Psoriasis
Source: Int J Mol Sci. 2021 Aug 10;22(16):8603. doi: 10.3390/ijms22168603 (PMC8395241; doi:10.3390/ijms22168603)
Supplement: Supplementary file 1 [file ijms-22-08603-s001.zip › Supplemental materials_Analysis of PPARg signaling activity in psoriasis/Pathway models/Models images and html files/Anti-psoriatic drugs influence PPARG signaling/94228.html]

vitamin D


# Small Molecule vitamin D

|  |  |
| --- | --- |
| URN | urn:agi-smol:vitamin%20D |
| Total Entities | 9 |
| Connectivity | 5503 |
| Name | vitamin D |
| Class | Endogenous compound |

---

|  |  |
| --- | --- |
| ChildConcepts | calciferol derivative |
|  | colecalciferol derivative |
|  | Ro23-7498 |
|  | 9,10 secocholesta 5,7,10(19) trien 23 yne 3,25 diol |
|  | Lunacalcipol |
|  | vitamin D analog |
|  | Ro23-7553 |
|  | Ro-23-8525 |
|  | 25-hydroxyvitamin D |

---

|  |  |
| --- | --- |
| Pathway | Vitamin D Activates Transcription |
|  | Vitamine D Deficite and Dentin Formation |
|  | Vitamin D and Folate in Multiple Sclerosis |
|  | Vitamins Insufficiency Causes Homocysteine High Level Synthesis |
|  | Vitamin D Represses Transcription |
|  | non-Genomic Rapid Actions of Vitamin D in Vitamin D Biology |
|  | Thymic Follicular Hyperplasia |
|  | Folate Cycle and Homocysteine Overproduction |
|  | hemophylia |
|  | Anti-psoriatic drugs influence PPARG signaling |

---

|  |  |
| --- | --- |
| MedScan ID | 1274900 |

---

|  |  |
| --- | --- |
| Alias | vitamin D |

---
