# Supplementary material for: Analysis of PPARγ Signaling Activity in Psoriasis
Source: Int J Mol Sci. 2021 Aug 10;22(16):8603. doi: 10.3390/ijms22168603 (PMC8395241; doi:10.3390/ijms22168603)
Supplement: Supplementary file 1 [file ijms-22-08603-s001.zip › Supplemental materials_Analysis of PPARg signaling activity in psoriasis/Pathway models/Models images and html files/Differentiation of psoriatic T cells/1012568724.html]

STAT4 --+> IFNG


# PromoterBinding STAT4 --+> IFNG

|  |  |
| --- | --- |
| URN | urn:agi-promoterbinding:in-out:urn:agi-llid:6775:out:urn:agi-llid:3458:eff=positive |
| Connectivity | 2 |
| References | 35 |
| Effect | positive |
| Original # of References | 15 |

---
