# Supplementary material for: Analysis of PPARγ Signaling Activity in Psoriasis
Source: Int J Mol Sci. 2021 Aug 10;22(16):8603. doi: 10.3390/ijms22168603 (PMC8395241; doi:10.3390/ijms22168603)
Supplement: Supplementary file 1 [file ijms-22-08603-s001.zip › Supplemental materials_Analysis of PPARg signaling activity in psoriasis/Pathway models/Models images and html files/Differentiation of psoriatic T cells/1012568725.html]

STAT1 --+> TBX21


# PromoterBinding STAT1 --+> TBX21

|  |  |
| --- | --- |
| URN | urn:agi-promoterbinding:in-out:urn:agi-llid:6772:out:urn:agi-llid:29939:eff=positive |
| Connectivity | 2 |
| References | 69 |
| Effect | positive |
| ObjectType | PromoterBinding |
| RelationArity | 2 |
| RelationNumberOfReferences | 36 |
| RelationSymbolicName | STAT1}OID --+> TBX21}OID |
| RelationConfidenceLevel | 3 |
| RelationParticipantTypes | Protein -> Protein |
| Original # of References | 3 |

---
